# Effect of Shenling Baizhu powder on immunity to diarrheal disease: A systematic review and meta-analysis

**DOI:** 10.3389/fphar.2022.938932

**Published:** 2022-09-14

**Authors:** Qian Chen, Zheng Xiao, Qing-Ying He, Rui-Rong Zhang, Shu-Xian Chen, Jia-Wei Dong, Hua Zhang, Xiao-Fan Chen

**Affiliations:** ^1^ Evidence-Based Medicine Research Centre, Jiangxi University of Chinese Medicine, Nanchang, Jiangxi, China; ^2^ Department of General Practice, Affiliated Hospital of Zunyi Medical University, Zunyi, Guizhou, China; ^3^ Department of Food Nutrition and Safety, College of Pharmacy, Jiangxi University of Chinese Medicine, Nanchang, Jiangxi, China

**Keywords:** Shenling Baizhu powder, diarrhea, immunity, systematic review, meta-analysis

## Abstract

**Background:** Diarrhea is one of the leading causes of death worldwide and is associated with immune dysfunction. The modulatory effects of Shenling Baizhu powder (SLBZS) on immune function in diarrheal disease have been validated in various animal models. However, the results of these studies have not been systematically evaluated. This study aimed to evaluate the preclinical data on SLBZS for the treatment of diarrhea from an immunological perspective.

**Methods:** PubMed, Embase, Cochrane Library, CNKI, Wanfang Database, VIP, and Chinese Medicine Database were searched for all animal trials on SLBZS for the treatment of diarrhea published up to April 2022. Standardized mean differences (SMD) were used as effect sizes in the meta-analysis of continuous variables, including immune organs, immune cells, and immune cytokines. Subgroup analysis was performed according to animal species and disease models. The GRADE was used to assess the quality of evidence.

**Results:** A total of 26 studies were included. Meta-analysis showed that compared to those in the model group, SLBZS significantly increased body weight [SMD = 1.54, 95% confidence interval (CI) (1.06, 2.02)], spleen mass [SMD = 1.42, 95% CI (0.98, 1.87)], thymus mass [SMD = 1.11, 95% CI (0.69, 1.53)], macrophage phagocytic capacity (SMD = 1.07, 95% CI [0.59, 1.54]), sIgA [SMD = 1.04, 95% CI (0.33, 1.74)], RBC-C3b-RR [SMD = 1.16, 95% CI (0.65, 1.67)], IL-2 [SMD = 1.52, 95% CI (0.89, 2.14)] and decreased diarrhea scores [SMD = −1.40, 95% CI (−2.03, −0.87)], RBC-IC-RR [SMD = −1.40, 95% CI (−1.94, −0.87)], and IL-8 [SMD = −2.80, 95% CI (−3.54, −2.07)]. Subgroup analysis showed that SLBZS regulated TNF-α, IL-1β, and IL-10 in rats and mice, and improved IL-6 and IL-10 in different diseases, with differences between subgroups (*p* < 0.05). Owing to heterogeneity, the reliability of the results remains to be verified. The quality of evidence was “very low”.

**Conclusion:** SLBZS improve diarrhea symptoms by enhancing immune function. It has curative effects with differences between different species and diseases, however, because the reporting in the original studies was too unclear to be assessed, the analysis was inconclusive. For higher quality evidences, future research should pay attention to the scientific rigor of the experimental design and the completeness of the reported results.

## 1 Introduction

**FIGURE 1:**
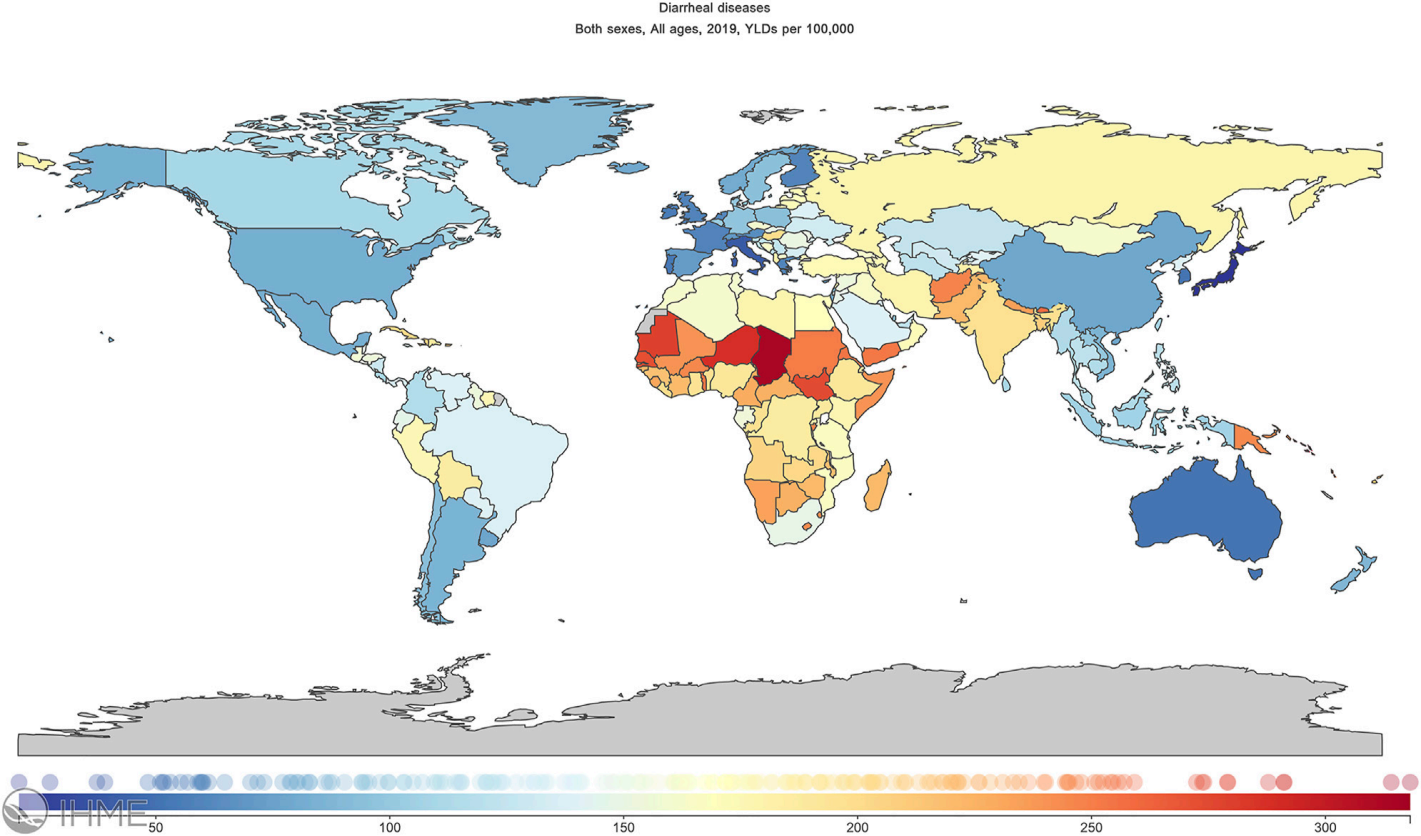
Global distribution map of years lived with disability relative to diarrhoeal disease.

According to the Global Burden of Disease, diarrhea is among the major public health problems worldwide and the eighth leading cause of death globally. In 2019, 1.5 million people, predominantly children and those over 70 years old, died from diarrhea (Collaborators, 2018; Collaborators, 2020). The Republic of Chad in Africa has the highest number of years lived with disability (314.06 per 100,000; Figure 1, https://ghdx.healthdata.org/), posing a substantial economic burden (Niyibitegeka et al., 2021).

Gastrointestinal (GI) disorders, including ulcerative colitis (UC), Crohn’s disease (CD), functional diarrhea (FD), acute colitis, and irritable bowel syndrome (IBS), present diarrhea and abdominal pain as their main clinical manifestations. The GI tract is home to 70% of the body’s lymphocytes and is the largest immune organ in the body, and its function is influenced by various lymphocytes and related factors (Takiishi et al., 2017).

The immune system consists of lymphoid organs, lymphoid tissues, and immune cells. The thymus and spleen are important lymphoid organs that reflect the overall immune function of the host (Lewis et al., 2019; Thapa and Farber, 2019; Wang et al., 2019). At the immune cell level, erythrocyte immunity was associated with a variety of digestive disorders, and C3b receptors on the surface of the erythrocyte membrane are an element of the body’s defense barrier that enhances immune function (Han, 2006). Macrophages, on the other hand, are responsible for adaptive immunity by regulating tissue homeostasis and inflammatory responses (Bronte and Pittet, 2013; Lewis et al., 2019). At the level of immune cytokines, secretory immunoglobulin A is the most abundant immunoglobulin secreted in the body and is the primary defense of the intestinal mucosa against the adhesion and colonization of pathogens. When immune organs, cells, and cytokines are dysfunctional, the immune system becomes disturbed and activates inflammatory responses in the GI (Chassaing et al., 2014), including abnormal expression of pro-inflammatory [interleukin (IL)-1β, IL-6, IL-8, and tumor necrosis factor-alpha (TNF-α)] and anti-inflammatory (IL-2, IL-4, and IL-10) cytokines, increased intestinal permeability, abnormal increase in body fluids at the point of entry into the intestinal lumen, increased fecal content, continuous stimulation of smooth muscles of the gut by active substances in the intestinal tract, and increased GI peristalsis, leading to diarrheal symptoms in patients (Lu and Zhou, 2022). Antispasmodics and anti-diarrheal agents are currently the main treatment for diarrhea (Wei et al., 2020; Li et al., 2021; Zhu and Zhao, 2021). These drugs provide rapid relief of symptoms; however, they have many side effects and a tendency to cause relapse.

In recent years, functional foods have become a priority in the management of diarrhea owing to their efficacy, safety, and sustainability (Tompkins et al., 2010). Shenling Baizhu powder (SLBZS) has been used for thousands of years as an edible plant product in China (Liu et al., 2022). SLBZS is composed of the following ten botanical drugs: *Panax ginseng* C.A.Mey (*Araliaceae*; ginseng radix), *Poria cocos* (*Polyporaceae*; Poria), *Atractylodes macrocephala* Koidz (*Asteraceae*; atractylodis macrocephalae rhizoma), *Lablab purpureus* subsp. Purpureus (*Fabaceae*; semen lablab album), *Dioscorea polystachya* Turcz (*Dioscoreaceae*; dioscoreae rhizoma), *Wurfbainia villosa* (Lour.) Skornick. & A.D.Poulsen (*Zingiberaceae*; amomi fructus), *Platycodon grandiflorus* (Jacq.) A. DC (*Campanulaceae*; platycodonis radix), *Coix lacryma-jobi* var. ma-yuen (Rom.Caill.) Stapf (*Poaceae*; coicis semen), *Glycyrrhiza glabra* L. (*Fabaceae*; radix et rhizoma glycyrrhizae), and *Nelumbo nucifera* Gaertn (Nelumbonaceae; lotus seed) (Supplementary Data Sheet S1). Its safety has been confirmed by several clinical studies (Mu and Xiao, 2016; Chen et al., 2019; Zhao and Cao, 2019). Modern pharmacological studies have demonstrated that the compounds β-carotene, β-sitosterol, ginsenoside Rh2, kaempferol, lignan, stigmasterol, and quercetin in SLBZS can improve the symptoms of diarrhea (Fiedor and Burda, 2014; Yu et al., 2016; Jakobsen et al., 2017; Shang et al., 2018); however, the exact mechanism is unclear.

This study aimed to systematically evaluate preclinical data on SLBZS for the treatment of diarrhea from an immunological perspective. Our findings can provide a scientific basis for health maintenance.

## 2 Materials and methods

The study was registered in the International Platform of Registered Systematic Review and Meta-Analysis Protocols (INPLASY^®^) under the registration number INPLASY202240132 and DOI number 10.37766/inplasy2022.4.0132. This systematic review was reported following the Preferred Reporting Items for Systematic Reviews and Meta-Analyses (PRISMA) guidelines (Supplementary Data Sheet S2).

### 2.1 Literature search strategy

Seven databases were searched, including PubMed, Embase, Cochrane Library, China National Knowledge Infrastructure (CNKI), Wanfang Database, VIP, and Chinese Medicine Database. The search dates were from database creation to April 2022. Search terms included “diarrhea,” “irritable bowel syndrome,” “ulcerative colitis,” “functional gastrointestinal disease,” “functional diarrhea,” “Crohn’s disease,” “inflammatory bowel disease,” “IBD,” “Shenling Baizhu powder,” “animals,” “rats,” and “mice.” The search strategies are listed in Supplementary Table S1.

### 2.2 Inclusion criteria

#### 2.2.1 Animals

All rodent animal models of diarrhea were included, with no restrictions on genus or animal modeling methods.

#### 2.2.2 Interventions

The experimental group was treated with SLBZS (unlimited dosage, frequency, and duration of treatment), whereas the control group was treated with saline or distilled water.

#### 2.2.3 Outcome indicators

This study focused on animal studies addressing immune modulation by SLBZS for the treatment of diarrhea; therefore, improvements in diarrhea symptoms, immune organs, and immune cells were used as the primary outcomes and derived inflammatory factors as the secondary outcomes.

The primary outcomes of the study were as follows: 1) overall condition: body weight and diarrhea score; 2) immune organ level: spleen weight and thymus weight; and 3) immune cell level: macrophage phagocytosis, red blood cell immune complex ring rate (RBC-IC-RR), red blood cell C3b receptor ring rate (RBC-C3b-RR), and sIgA.

The secondary outcomes were the following: pro-inflammatory (TNF-α, IL-1β, IL-8, and IL-6) and anti-inflammatory (IL-2, IL-4, and IL-10) factors.

#### 2.2.4 Type of study

All animal trials with randomization method were included, no restrictions on the language of publication.

### 2.3 Exclusion criteria

The exclusion criteria were as follows: 1) studies that did not focus on diarrhea as the main symptom; 2) the experimental group was not treated with SLBZS alone; studies with missing outcome indicators and missing and incomplete data; clinical studies, *in vitro* experiments, review literature, and farm animal husbandry studies; and duplicate publications.

### 2.4 Data extraction and literature quality evaluation

Data extraction was performed by four researchers and an expert from a screening and extraction team. Two researchers (R-RZ and J-WD) screened the literature according to the inclusion and exclusion criteria and selected the studies to be included in the initial analysis. Two other researchers (QC and QH) extracted the following information from the studies: 1) basic information: title, author, and year; 2) subjects: animal species, body weight, disease model, and sample size; 3) interventions: drug, dose, and duration; 4) outcome indicators; and 5) SLBZS: composition and proportion of each component. Any disagreement during this process was resolved by the expert.

The quality of the included studies was reviewed using the SYRCLE risk of bias tool. The tool was used to assess recommended items in 10 domains from the following six areas: selection bias, implementation bias, measurement bias, data integrity bias, reporting bias, and other bias. For each domain, studies were rated “Y,” indicating a low risk of bias; “N,” indicating a high risk of bias; or “U,” indicating insufficient detail in reporting.

### 2.5 Statistical analysis

Review Manager 5.3 and Stata 17 were used to perform the meta-analysis. Standardized mean differences (SMD) were used to take into account variability in methods of animal experimentation, animal species, and study units. When *I* ^2^ ≤ 50% and *p* > 0.1, a good homogeneity between studies was judged, and a fixed-effects model was used. When *I* ^2^ > 50% and *p* ≤ 0.1, sensitivity and subgroup analyses were conducted to explore the sources of heterogeneity, and a random-effects model was used for the results that remained highly heterogeneous. When heterogeneity was not resolved, descriptive analysis was considered. The Egger’s test was used to assess publication bias; when publication bias was present, the stability of the results was assessed using the trim-and-fill analysis.

### 2.6 Grading the quality of evidence

The quality of each piece of evidence was assessed using the Grading of Recommendations Assessment, Development, and Evaluation (GRADE) approach (Guyatt et al., 2011), which includes the following five dimensions: risk of bias, inconsistency, indirectness, imprecision, and publication bias. The quality of the studies was classified as “very low,” “low,” “medium,” and “high.” The risk of bias was assessed based on SYRCLE risk results. Inconsistency was considered based on the size and direction of each study, *p*-value, and *I* ^2^ value of the heterogeneity test; downgrading was considered when the heterogeneity was high and could not be explained. Indirectness was determined based on species, animal model matching, and baseline animal attributes. Imprecision was evaluated based on whether the sample reached the optimal information sample size and the width of the confidence interval (CI). Publication bias was graded as “detected” or “undetected” according to statistical methods, such as funnel plots and Egger’s test.

## 3 Results

### 3.1 Study selection

**FIGURE 2:**
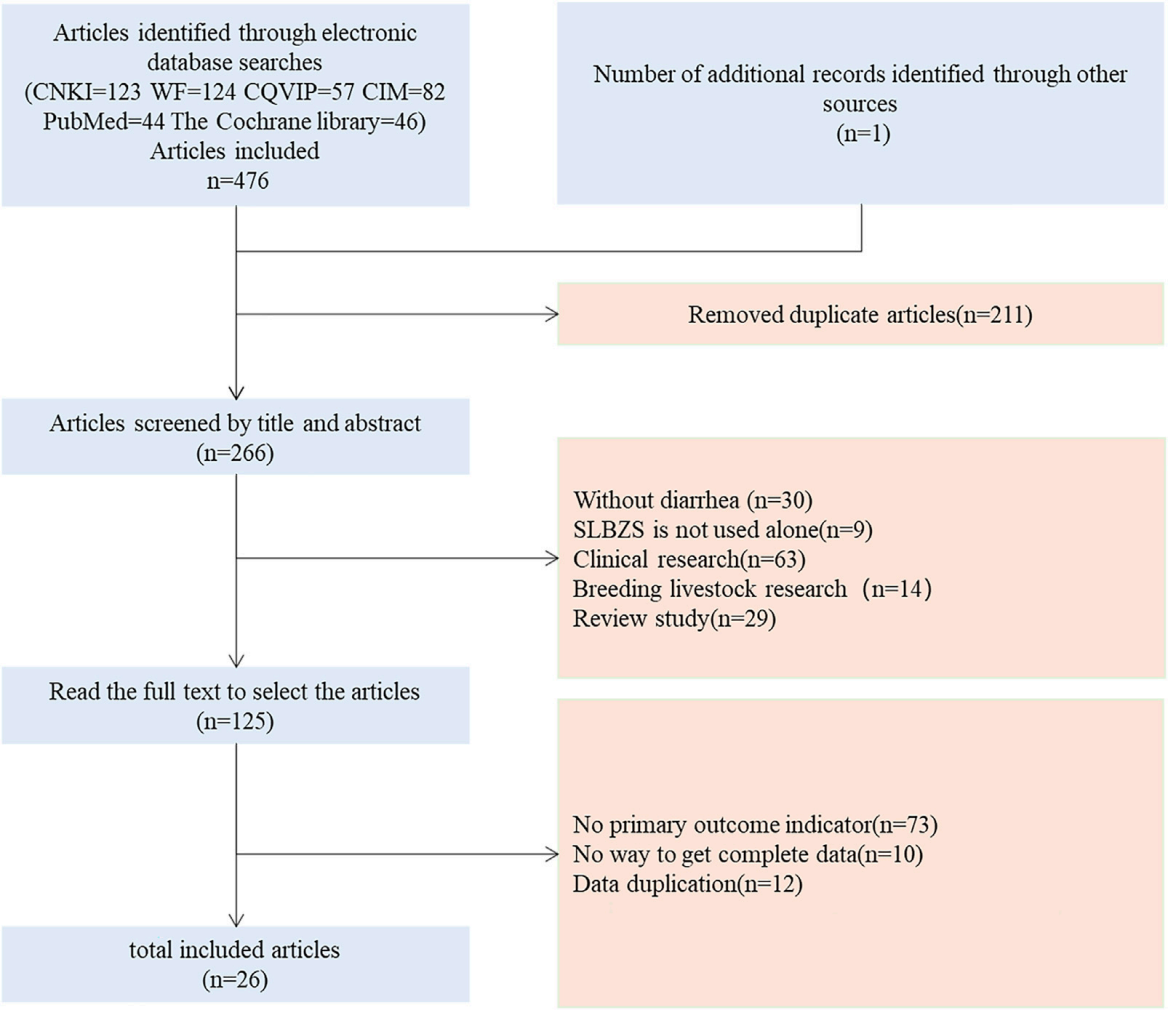
Literature screening flow chart.

According to the search strategy, 477 relevant articles were obtained. After duplicate articles (*n* = 211) were excluded, 30 studies not related to diarrhea, 9 studies not reporting on SLBZS alone, 63 clinical studies, 14 studies on farm animal husbandry, and 29 reviews were excluded by screening the titles and abstracts. After reading the full text, 73 studies without relevant outcome indicators, 10 studies for which accurate data were not available, and 12 studies with similar or duplicate data were excluded; finally, 26 studies were included in the analysis (Figure 2).

### 3.2 Basic characteristics and quality assessment of the included studies

The 26 studies analyzed 685 animals in total (Cheng et al., 2004; Fan et al., 2004; Zhang et al., 2006; Chen, 2007; Du, 2007; Han et al., 2008; Jiang, 2010; Liu, 2010; Zhanag, 2010; Ding et al., 2011; Li et al., 2014; Li et al., 2015a; Li et al., 2015b; Jia et al., 2016; Li et al., 2016; Bi et al., 2017; LI et al., 2017; Yu 2017; Shi and Yu, 2018; Yu et al., 2018; You et al., 2019; Zhang et al., 2019; Zhou et al., 2019; Li et al., 2020; Sun et al., 2020; Tong, 2021), and all experiments were conducted in China and published in Chinese. In terms of animal type, 13 studies were performed on rats (Fan et al., 2004; Du, 2007; Ding et al., 2011; Li et al., 2015b; Jia et al., 2016; Li et al., 2016; Bi et al., 2017; LI et al., 2017; Yu 2017; Yu et al., 2018; Zhou et al., 2019; Li et al., 2020; Tong, 2021), and 13 on mice (Cheng et al., 2004; Zhang et al., 2006; Chen, 2007; Han et al., 2008; Jiang, 2010; Liu, 2010; Zhanag, 2010; Li et al., 2014; Li et al., 2015a; Shi and Yu, 2018; You et al., 2019; Zhang et al., 2019; Sun et al., 2020). Regarding diarrhea models, 11 studies constructed a UC model (Fan et al., 2004; Li et al., 2014; Li et al., 2015b; Jia et al., 2016; Bi et al., 2017; LI et al., 2017; Yu et al., 2018; Zhang et al., 2019; Zhou et al., 2019; Li et al., 2020; Tong, 2021); nine studies (Chen, 2007; Du, 2007; Han et al., 2008; Jiang, 2010; Liu, 2010; Zhanag, 2010; Li et al., 2015a; Shi and Yu, 2018; Sun et al., 2020) a spleen deficiency diarrhea model (SDDM); and chronic diarrhea (Cheng et al., 2004), delayed diarrhea (Ding et al., 2011), FD (Li et al., 2016), IBS (You et al., 2019), CD (Yu 2017), and colitis (Zhang et al., 2006) models were reported in only one article respectively. Treatment duration ranged from 7 to 21 days, with <14 days in 11 studies (Cheng et al., 2004; Zhang et al., 2006; Chen, 2007; Han et al., 2008; Jiang, 2010; Liu, 2010; Zhanag, 2010; Ding et al., 2011; Li et al., 2014; Li et al., 2015a; Sun et al., 2020) and ≥14 days in 15 studies (Fan et al., 2004; Du, 2007; Li et al., 2015b; Jia et al., 2016; Li et al., 2016; Bi et al., 2017; LI et al., 2017; Yu 2017; Shi and Yu, 2018; Yu et al., 2018; You et al., 2019; Zhang et al., 2019; Zhou et al., 2019; Li et al., 2020; Tong, 2021). In terms of dose, only 10 studies (Ding et al., 2011; Li et al., 2014; Jia et al., 2016; Li et al., 2016; Bi et al., 2017; LI et al., 2017; Yu et al., 2018; You et al., 2019; Zhang et al., 2019; Tong, 2021) had different dose interventions. There were no differences in the animal type, model construction, duration, and dose between the experimental and control groups within the same study. The baseline characteristics of each study are shown in Table 1. The composition of SLBZS, including the specific weights and proportion of each component, is shown in Supplementary Table S2. None of these studies performed a chemical analysis of SLBZS.

**TABLE 1:** Characteristics of the included studies.

Studies	Animals	Weight(g)	Animal model	Modeling method	Sample size	Interventions (SLBZS)	Intervention (model)	Time	Outcome
BDY2017	Wistar rats	180 ± 20	UC	Lard (1. 6 g/kg, qod) + standing in 2 cm deep water (qd, 8 h/d) + fasting for 1 day (qod), 21d	8/8	△24 g/kg; □12 g/kg; ◇6 g/kg	DW	Qd, 21d	(9) (11)
CH2007	Kunming mice	20 ± 2	SDDM	100% Senna leaf decoction,0.5 ml, bid, 20d	10/10	0.12 g/ml; 0.4–0.6 ml	NS	Qd, 10d	(1)(3)(4)(6)(7)(8)
CCH2004	Kunming mice	20 ± 1	Chronic Diarrhoea	100% raw rhubarb decoction, 0.5 ml, bid, 8d	10/10	2.34 g/kg	NS	qd, 7d	(3)(4)(5)
DJL2011	SD rats	160 ± 20	Delayed Diarrhoea	Caudal vein injection CPT-11,150 mg/kg/d, 2d	8/8	△8.2 g/kg; □5.58 g/kg; ◇3.51 g/kg	DW	qd, 10d	(2)(12)(14)(15)
DYW2007	Wistar rats	240 ± 30	SDDM	100% raw rhubarb decoction, 5 ml/100 g, 7d	8/8	1.5 ml/100 g; Dc 0.108 g/ml	DW	qd, 14d	(8)(10)
FH2004	SD rats	300 ± 50	UC	0.1% 2,4-dinitrochlorobenzene 0.25 ml Enema +8% acetic acid enema 2 ml, 20 s	9/10	0.9879 g/ml 3 ml	DW	qd, 15d	(12)(13)(14)(15)
HHR2008	Kunming mice	20 ± 2	SDDM	100% raw rhubarb decoction, 0.4 ml/20 g, qd, 8d	15/15	4 ml/20 g; Dc 0.176 g/ml	NS	qd, 7d	(1)(3)(4)(5)(6)(7)
JYX2016	Wistar rats	160 ± 20	UC	Lard (2 ml, qd) + standing in 2 cm deep water (qd, 8 h/d) + fasting for 1 day (qod), 21 d	10/10	△24 g/kg; □12 g/kg; ◇6 g/kg	DW	qd, 21d	(9)(11)
JMR2010	Kunming mice	20 ± 3	SDDM	100% Senna leaf decoction, 0.5 ml, bid, 20d	10/10	0.12 g/m 0.5 ml	NS	bid, 10d	(1)(6)(7)
LJ2016	Wistar rats	85–95	FD	High lactose feed + forced standing 10 h, 14d	15/15	△3.212 g/kg; □1.606 g/kg; ◇0.803 g/kg	NS	tid, 14d	-15
LWX2015	Kunming mice	20 ± 2	SDDM	100% Senna leaf decoction, 0.5 ml, 10d	10/10	0.02 ml/g	DW	qd, 7d	(2)(5)
LXB2014	Kunming mice	20 ± 2	UC	TNBS/ethanol solution Enema (3 mg/ml, 40% ethanol) 0.1 ml, 1 min, qd	10/10	△560 g/kg; □280 g/kg; ◇140 g/kg	NS	qd, 10d	(9)(14)(15)
LY2017	SD rats	220 ± 10	UC	50%TNBS/ethanol solution (100 mg/kg), 0.8 ml, qd	14/14	△3 g/kg; □1.5 g/kg; ◇0.75 g/kg	NS	qd, 15d	(1)(12)(14)(15)
LZH2015	Wistar rats	200 ± 20	UC	Lard (4 ml, qod) + Ice water gavage (2 ml, qod), 20d; 5%TNBS + 50%ethanol solution, 0.8 ml	12/12	12 g/kg	NS	qd, 14d	(12)(13)(15)
LZH2020	Wistar rats	200 ± 20	UC	Lard (4 ml, qod) + Ice water gavage (2 ml,qod),20d; 5%TNBS+50%ethanol solution, 0.8 ml	12/12	15.6 g/kg	NS	qd, 14d	(12)(13)(15)
LHW2010	Kunming mice	20 ± 3	SDDM	100% Senna leaf decoction 0.5 ml, bid, 20d	10/10	0.12 g/m 0.5 ml	NS	bid, 10d	(3)(4)(5)
SY2018	Kunming mice	20 ± 2	SDDM	Kale and lard (1 ml/100 g, qod) + forced swimming, 14 d	10/10	1.2 g/100 g	NS	bid, 14d	(1)(3)(4)(8)(10)(15)
SSZ2020	C57BL/6 Mice	18 ± 5	SDDM	100% Senna leaf decoction,0.2 ml/10 g, 14d	10/10	0.2 ml/10 g; Dc 0.45 g/ml	DW	qd, 7d	(9)(15)
TJS2021	SD rats	225 ± 15	UC	5%TNBS 100 mg/kg + 50% ethanol 0.25 ml, once	12/12	△2.4 g/ml; □1.2 g/ml; Dc 10 ml/kg	DW	qd, 21d	(12)(14)
YY2019	BALB/C Mice	20 ± 2	IBD	5% DSS solution free drinking, 7d	12/12	△12 g/kg; □6 g/kg; ◇3 g/kg	—	qd, 15d	(13)(14)
YHQ2017	Wistar rats	180 ± 20	CD	TNBS/ethanol solution (120 mg/kg), 1 min	10/10	12 g/kg	NS	qd, 21d	(9)(11)(14)
YHQ2018	Wistar rats	180 ± 20	UC	Disease + Symptoms	10/10	△24 g/kg; □12 g/kg; ◇6 g/kg	NS	qd, 21d	(12)(14)
ZJJ2019	C57BL/6 Mice	20 ± 2	UC	3% DSS solution free drinking, 7d	10/10	△31.2 g/kg; □15.6 g/kg; ◇7.8 g/kg	NS	qd, 14d	-9
ZJL2010	Kunming mice	20 ± 3	SDDM	100% Senna leaf decoction 0.5 ml, bid, 20d	10/10	0.12 g/ml	NS	bid, 10d	(1)(10)
ZYF2006	BALB/C Mice	21 ± 1	colitis	3% DSS solution free drinking, 7d	10/10	31 g/kg	—	qd, 10d	(11)(14)
ZH2019	SD rats	180 ± 20	UC	DSS solution free drinking (40 g/L)	8/8	24 g/kg	NS	qd, 21d	(1)(12)(14)(15)

Shenling Baizhu powder (SLBZS); ulcerative colitis (UC); spleen deficiency diarrhea model (SDDM); functional diarrhea (FD); inflammatory bowel disease (IBD); drug concentration (Dc); normal saline (NS); distilled water (DW); qd △SLBZS high -dose group; □SLBZS middle-dose group; ◇SLBZS low -dose group; body weight (1); diarrhoea score (2); spleen mass index (3); thymus index (4); phagocytosis of macrophages (5); red blood cell C3b receptor ring rate (6); red blood cell immune complex ring rate (7); secretory immunoglobulin A (8); interleukin (IL)-1β (9); IL-2 (10); IL-4 (11); IL-6 (12); IL-8 (13); IL-10 (14); tumour necrosis factor-α (15).

**FIGURE 3:**
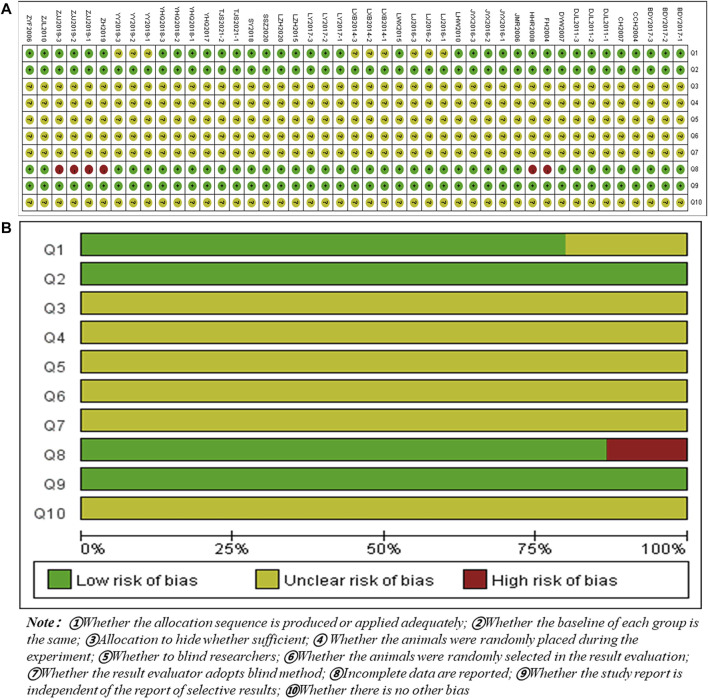
**(A)** Risk of bias summary. **(B)** Risk of bias graph.

Overall, the risk of bias assessment was mostly “unclear,” indicating that most items were under-reported and could lead to an unknown risk of bias. In terms of selective bias (Q1–Q3), 26 papers mentioned random grouping, and only three (Li et al., 2014; Li et al., 2016; You et al., 2019) did not mention the grouping method and could not be identified. All were reported at baseline, and no mention was made of allocation concealment. Implementation bias (Q4–Q5) was not explicitly reported in all studies. All studies did not describe the random selection of animals in the outcome evaluation and whether the evaluators used blinding (Q6–Q7). In four studies (Han et al., 2008; Jia et al., 2016; Zhang et al., 2019; Zhou et al., 2019), incomplete data were not adequately addressed, and the cause of death of animals and how they were handled were not mentioned, resulting in a high risk of bias (Q8). In terms of reporting bias, all studies were rated as low risk (Q9). In addition, it was unclear whether other biases existed (Q10) (Figure 3).

### 3.3 Statistical results

#### 3.3.1 Meta-analysis results

##### 3.3.1.1 Body weight

**FIGURE 4:**
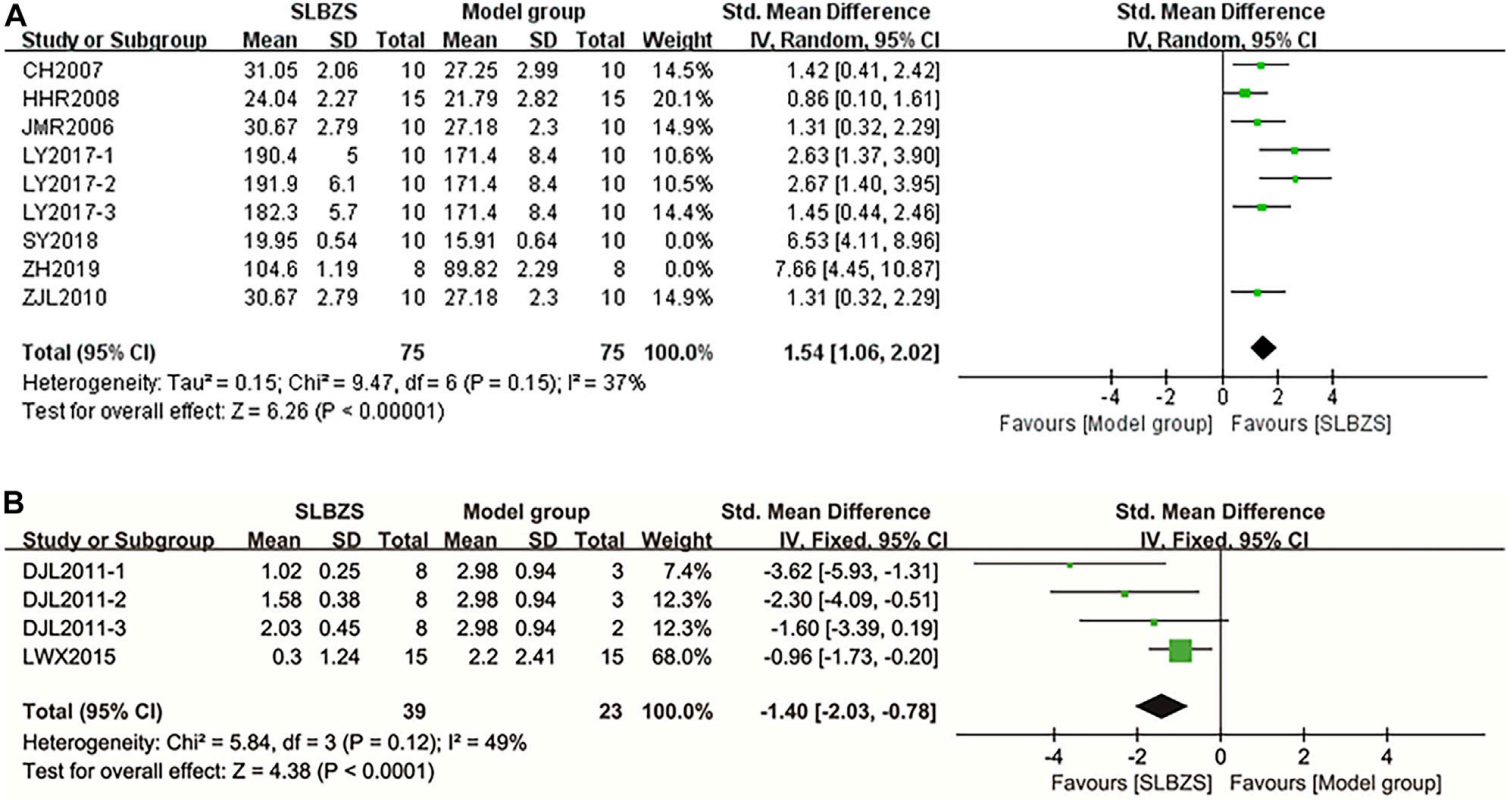
**(A)** The results of body weight. **(B)** The results of diarrhea scores.

Body weight was measured in seven studies (Chen, 2007; Han et al., 2008; Jiang, 2010; Zhanag, 2010; LI et al., 2017; Shi and Yu, 2018; Zhou et al., 2019). There was a significant heterogeneity within groups (*I* ^2^ = 78%). Since ZH2019 reported values for weight change, the original study SY2018 simply described the data for weight and did not perform a statistical analysis of the data, both studies were excluded. Sensitivity analysis showed a significant increase in body weight in the SLBZS group compared to that in the control group [SMD = 1.54, 95% CI (1.06, 2.02), *p* < 0.00001, *I* ^2^ = 37%] (Figure 4A).

##### 3.3.1.2 Diarrhea scores

Diarrhea scores were assessed in two studies (Ding et al., 2011; Li et al., 2015a). The results of the meta-analysis showed a significant improvement in diarrhea symptoms in the SLBZS group compared to those in the control group [SMD = −1.40, 95% CI (−2.03, −0.78)] (Figure 4B).

##### 3.3.1.3 Immune organs

###### 3.3.1.3.1 Spleen/thymus index

**FIGURE 5:**
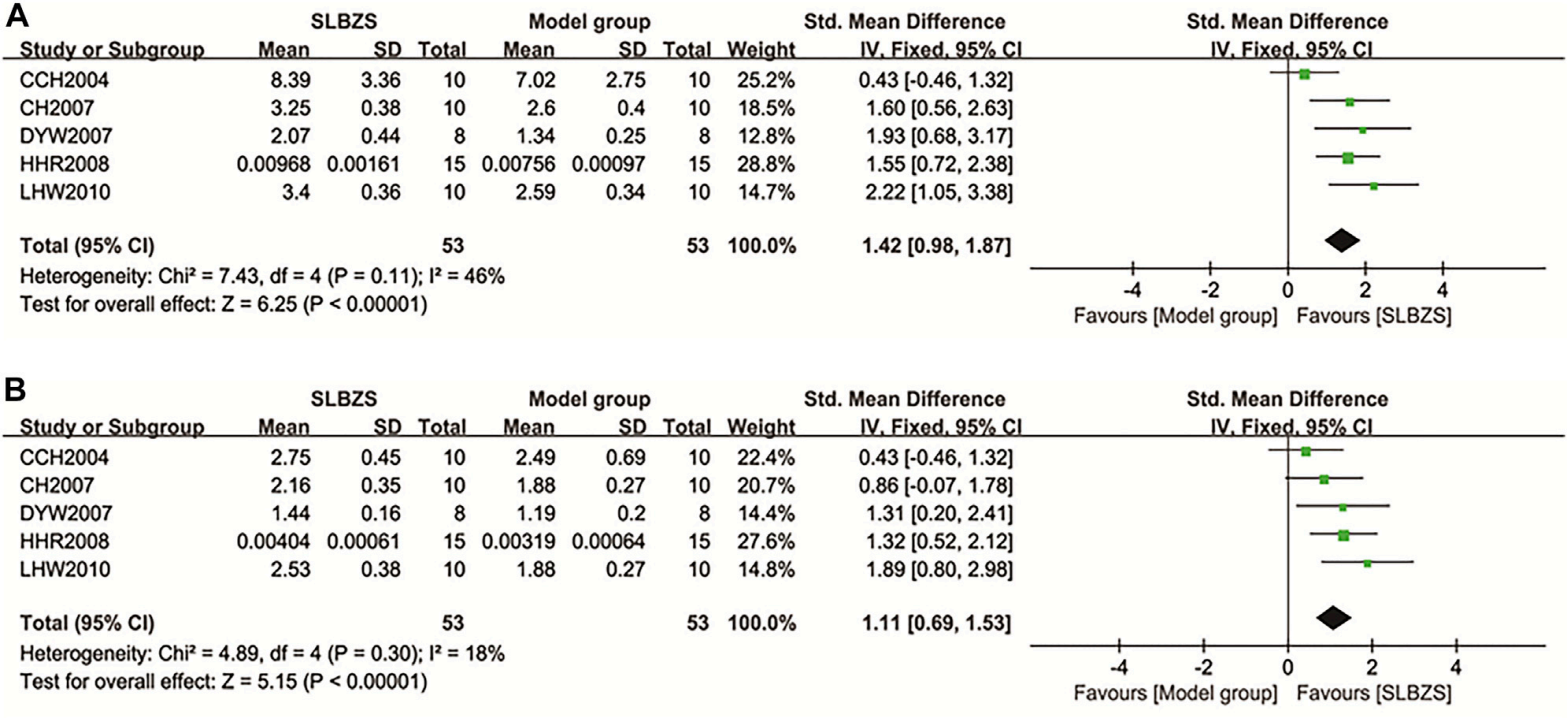
**(A)** The results of spleen index. **(B)** The results of thymus index.

Spleen/thymus indexes were reported in five studies (Cheng et al., 2004; Chen, 2007; Han et al., 2008; Liu, 2010; Shi and Yu, 2018), none of which had significant heterogeneity within groups (*I* ^2^ < 50%, *p* > 0.1) using a fixed-effects model. The SLBZS group was effective in increasing spleen [SMD = 1.42, 95% CI (0.98, 1.87), *p* < 0.00001] and thymus indexes [SMD = 1.11, 95% CI (0.69, 1.53), *p* < 0.00001] compared to the control group (Figures 5A,B).

##### 3.3.1.4 Immune cells

###### 3.3.1.4.1 Macrophage phagocytic capacity

**FIGURE 6:**
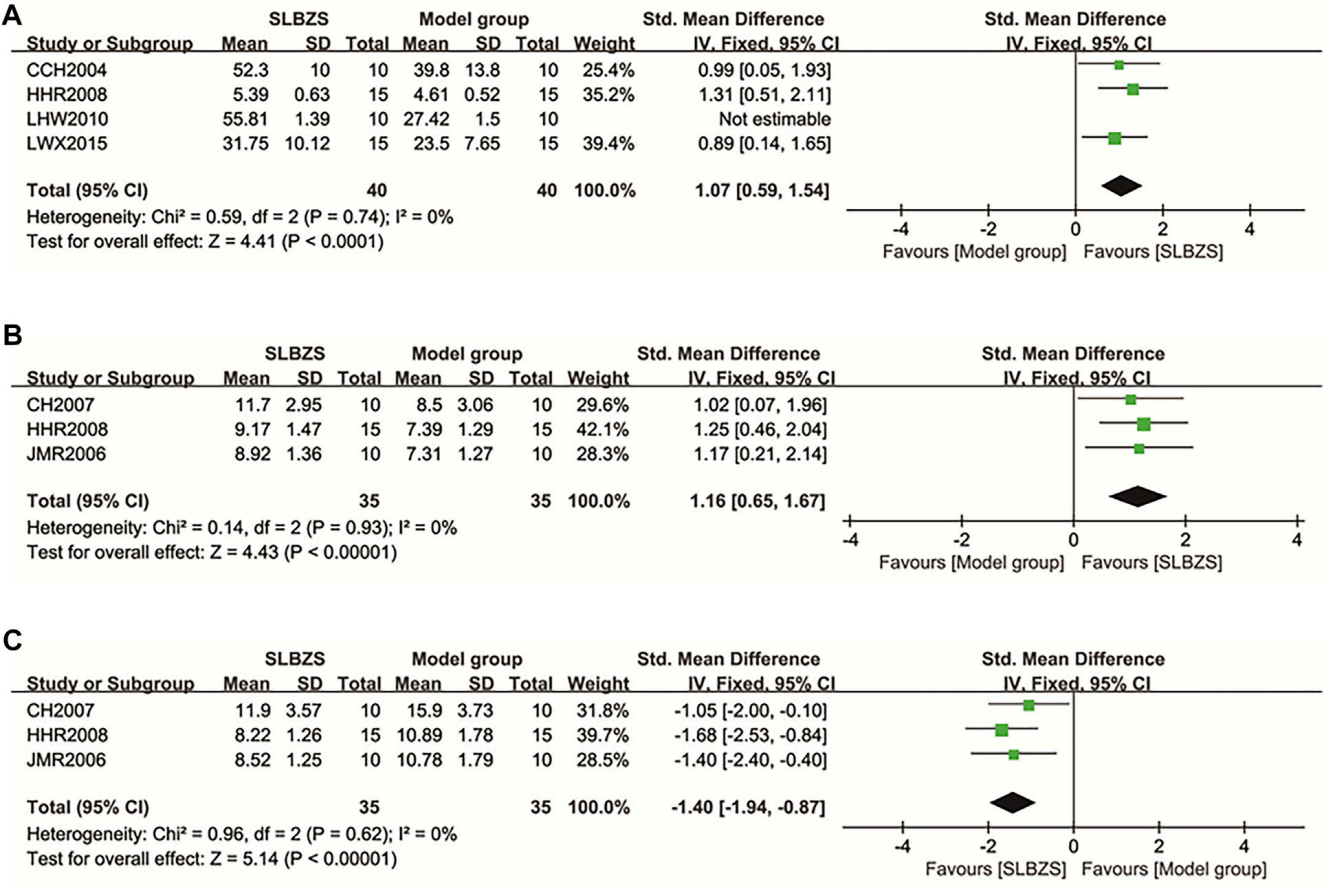
**(A)**The results of macrophage phagocytic capacity. **(B)** The results of RBC-C3b-RR. **(C)** The results of RBC-IC-RR.

Four studies (Cheng et al., 2004; Han et al., 2008; Liu, 2010; Li et al., 2015a) reported on macrophage phagocytic capacity. There was a large within-group heterogeneity (*I* ^2^ = 89%), and sensitivity analyses were performed. The counting method for LHW2010 differed from those of other studies, and *I* ^*2*^ = 0% after its exclusion. The SLBZS group showed increased macrophage phagocytosis compared to the control group [SMD = 1.07, 95% CI (0.59, 1.54), *p* < 0.0001, *I* ^2^ = 0%] (Figure 6A).

###### 3.3.1.4.2 Red blood cell C3b receptor ring rate and red blood cell immune complex ring rate

Three studies (Chen, 2007; Han et al., 2008; Jiang, 2010) examined RBC-C3b-RR and RBC-IC-RR. There was no significant heterogeneity within groups (*I* ^2^ = 0%, *p* > 0.1) using a fixed-effects model. The SLBZS group resulted in an effective increase in RBC-C3b-RR [SMD = 1.16, 95% CI (0.65, 1.67) *p* < 0.00001] levels and a decrease in RBC-IC-RR [SMD = −1.40, 95% CI (−1.94, −0.87), *p* < 0.00001] levels compared to the control group (Figures 6B,C).

##### 3.3.1.5 Immunocytokines

###### 3.3.1.5.1 Secretory immunoglobulin

**FIGURE 7:**
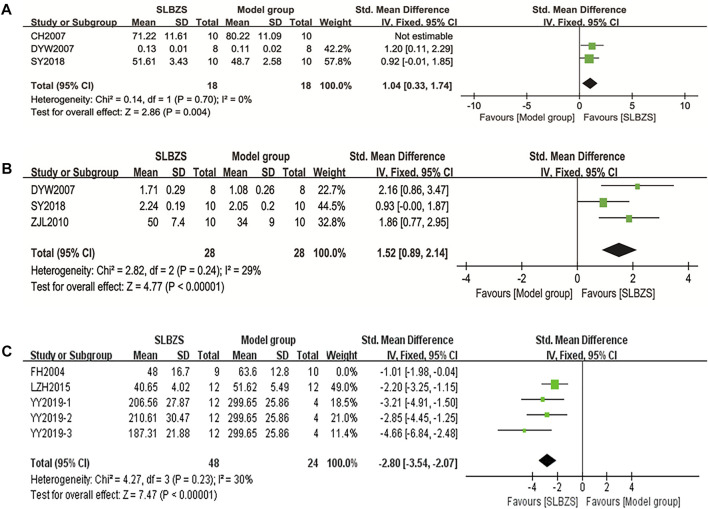
**(A)** The results of sIgA. **(B)** The results of IL-2. **(C)** The results of IL-8.

Three studies (Chen, 2007; Du, 2007; Shi and Yu, 2018) analyzed sIgA, with a significant heterogeneity within groups (*I* ^2^ = 79%). Sensitivity analysis found that CH2007 measured sIgA extracted from mucus rather than mucosal tissues; when this study was excluded, *I* ^2^ = 0%. Using a fixed-effects model, the SLBZS group led to an effective increase in sIgA levels [SMD = 1.04, 95% CI (0.33, 1.74), *p* = 0.004] (Figure 7A).

###### 3.3.1.5.2 Interleukin-2

Three studies (Du, 2007; Zhanag, 2010; Shi and Yu, 2018) evaluated IL-2, with no significant heterogeneity within groups (*I* ^2^ = 29%, *p* = 0.24). IL-2 levels were increased in the SLBZS group compared to those in the control group [SMD = 1.52, 95% CI (0.89, 2.14), *p* < 0.00001] (Figure 7B).

###### 3.3.1.5.3 Interleukin-8

Four studies (Fan et al., 2004; Li et al., 2015b; You et al., 2019; Li et al., 2020) reported on IL-8, and the results showed some heterogeneity within groups (I^2^ = 68%, *p* = 0.01). Sensitivity analysis found that FH2014 measurement of IL-8 was performed using colonic tissue, whereas the other studies used serum and were therefore excluded. The SLBZS group was able to reduce IL-8 levels compared to the control group [SMD = −2.80, 95% CI (−3.54, −2.07)], *p* < 0.00001, *I* ^2^ = 30%) (Figure 7C).

#### 3.3.2 Results of descriptive analysis

Meta-analysis was performed on all outcome indicators, and the results showed a large heterogeneity in the five indicators of immune cytokines (TNF-α, IL-1β, IL-6, IL-4, and IL-10). To explore the source of heterogeneity, subgroup analysis, and sensitivity analysis were performed (Supplementary Figures S1–S16). The results showed significant differences between groups in animal species and disease models (*p* < 0.05) and no significant differences in dose and intervention period, suggesting that the heterogeneity may originate from animal species and different diseases. After subgroup and sensitivity analyses, the issue of heterogeneity was not addressed. Hence, we performed a descriptive analysis of the five indicators.

##### 3.3.2.1 Tumor necrosis factor alpha

Ten studies (Fan et al., 2004; Ding et al., 2011; Li et al., 2014; Li et al., 2015b; Li et al., 2016; LI et al., 2017; Shi and Yu, 2018; Zhou et al., 2019; Li et al., 2020; Sun et al., 2020) evaluated TNF-α. TNF-α levels were significantly lower in the SLBZS group than in the model group [SMD = −3.96, 95% CI (−5.32, −2.61), *I* ^2^ = 91%] Seven studies used rats and three studies used mice. SLBZS was effective in both rats and mice. In terms of disease models, six studies reported UC, two SDDM, one FD and one delayed diarrhoea, efficacy was observed in all disease models. Four studies investigated dose-effect relationships and reported a better efficacy of SLBZS at higher doses (*p* < 0.05); however, owing to sample size limitations, we can only discuss trends in the studies and cannot do a simple combination of effect sizes. Notably, SY2018 obtained contrasting results compared to other studies, with SLBZS elevating the expression of TNF-α.

##### 3.3.2.2 Interleukin-1β

Seven studies (Li et al., 2014; Jia et al., 2016; Bi et al., 2017; Yu 2017; Zhang et al., 2019; Li et al., 2020; Sun et al., 2020) analyzed IL-1β. Overall, SLBZS was effective in downregulating IL-1β levels compared to those in the model group [SMD = −3.19, 95% CI (−4.31, −2.06), *I* ^2^ = 81%]. The overall efficacy in rats (Jia et al., 2016; Bi et al., 2017; Yu 2017; Li et al., 2020) and mice were observed, and four studies reported dose-effect relationships, with higher doses of SLBZS demonstrating better efficacy in reducing IL-1β levels in all studies (*p* < 0.05).

##### 3.3.2.3 Interleukin-6

IL-6 was reported in eight studies (Fan et al., 2004; Ding et al., 2011; Li et al., 2015b; LI et al., 2017; Yu et al., 2018; Zhou et al., 2019; Li et al., 2020; Tong, 2021), all using rats. The results showed that IL-6 levels were significantly lower in the SLBZS group than in the model group [SMD = −4.35, 95% CI (−5.40, −3.29), *I* ^2^ = 75%]. The efficacy of SLBZS was significant for delayed diarrhea. In terms of dose, better efficacy was reported with higher doses of SLBZS (*p* < 0.05).

##### 3.3.2.4 Interleukin-4

Four studies (Zhang et al., 2006; Jia et al., 2016; Bi et al., 2017; Yu 2017) reported on IL-4. IL-4 levels were significantly higher in the SLBZS group than in the model group [SMD = 3.43, 95% CI (1.26, 5.60), I^2^ = 91%]. However, ZYF2006 obtained a different result compared to the other three studies: SLBZS reduced IL-4 expression and was the only one of all studies reporting IL-4 using mice. Two studies reported dose-effect relationships, with higher doses of SLZBS leading to better regulation of IL-4 expression (*p* < 0.05).

##### 3.3.2.5 Interleukin-10

IL-10 was investigated in 10 studies (Fan et al., 2004; Zhang et al., 2006; Ding et al., 2011; Li et al., 2014; LI et al., 2017; Yu 2017; Yu et al., 2018; You et al., 2019; Zhou et al., 2019; Tong, 2021). Overall, IL-10 levels were significantly higher in the SLBZS group than in the model group [SMD = 3.77, 95% CI (2.98, 4.56), *I* ^2^ = 71%]. Efficacy was observed in both rats and mice. In disease models, SLBZS reduced the inflammatory response in UC, IBD, CD, delayed diarrhea, and colitis, with different effect sizes for each disease. Six studies reported a dose-effect relationship, with high doses having the best efficacy (*p* < 0.05).

### 3.4 Publication bias

**FIGURE 8:**
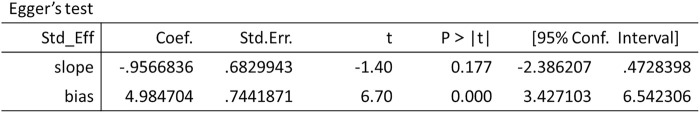
Publication bias analysis.

As more than 10 studies reported on IL-10, we used Egger’s test to objectively identify publication bias in these studies. The results showed the presence of publication bias (*p* < 0.01) (Figure 8). The results of the trim-and-fill analysis showed that after eight iterations, the linear method estimated the number of missing studies to be seven (diff = 0). After the inclusion of the missing studies, the results were tested for heterogeneity (*Q* = 132.316, *p* < 0.001). The combined result for the resulting effect indicator was 22.591 [95% CI (9.383, 54.391)]. There was no reversal of results after the inclusion of the seven missing studies, indicating that the results were relatively stable (Supplementary Figure S17).

### 3.5 Quality of evidence

We evaluated the quality of body weight, diarrhea scores, spleen/thymus index, macrophage phagocytic capacity, RBC-C3b-RR, RBC-IC-RR, sIgA, IL-2, and IL-8. The quality of evidence as assessed by GRADE was graded as “very low,” as it was downgraded for risk of bias, indirectness, and imprecision. The findings are summarized in Supplementary Table S3, which describes the complete assessment with footnotes explaining each judgment.

## 4 Discussion

### 4.1 Summary of the evidence

We included 26 studies and conducted a systematic review and meta-analysis of 15 outcomes. Our results showed that SLBZS was effective in improving diarrheal symptoms, increasing spleen, and thymus mass, enhancing macrophage phagocytosis and RBC-C3b-RR, reducing RBC-IC-RR and the production of pro-inflammatory factors (IL-1β, IL-6, IL-8, and TNF-α), increasing the levels of anti-inflammatory factors (IL-2, IL-4, and IL-10), and significantly enhancing the organism’s immunity. However, all results had “very low” quality.

The results of the subgroup analysis showed that in terms of animal species, SLBZS was effective in both rats and mice, and there were differences between groups, it may be attributed to the fact that rats are more similar to primates in terms of rectal temperature and normal leukocyte content than mice (Gross, 2009). In a comparison of different disease models, SLBZS has improved effect on all diarrhea diseases, among which the effect size of delayed diarrhea caused by chemotherapy drug irinotecan (CPT-11) was the highest. When CPT-11 was administered, the body’s immunity was reduced, and the levels of inflammatory cytokines increased, leading to diarrhea (Kon et al., 2018). SLBZS regulated the production of inflammatory factors TNF-α, IL-6, and IL-10 and enhanced the body’s immunity, which may be the reason for its higher efficacy against delayed diarrhea. Although some original studies suggested that higher doses of SLBZS were more effective in improving immune status in diarrheal disease, the results of our meta-analysis suggested no significant differences between doses; moreover, owing to heterogeneity, the reliability of the results remains to be verified.

### 4.2 Mechanism by which Shenling Baizhu powder modulates immunity to improve diarrhea

The immune system is a key factor in preventing pathogens from colonizing the host and causing disease (Perez-Lopez et al., 2016). Diarrheal diseases are closely associated with dysfunctional immune responses (Ungaro et al., 2017). Reduced mass of the immune organs, spleen, and thymus; decreased phagocytosis of macrophages; and reduced erythrocyte immune function and inflammatory responses often accompany diarrhea, and modulation of these indicators can be effective in improving diarrheal symptoms (Han et al., 2008; Ding et al., 2011).

**FIGURE 9:**
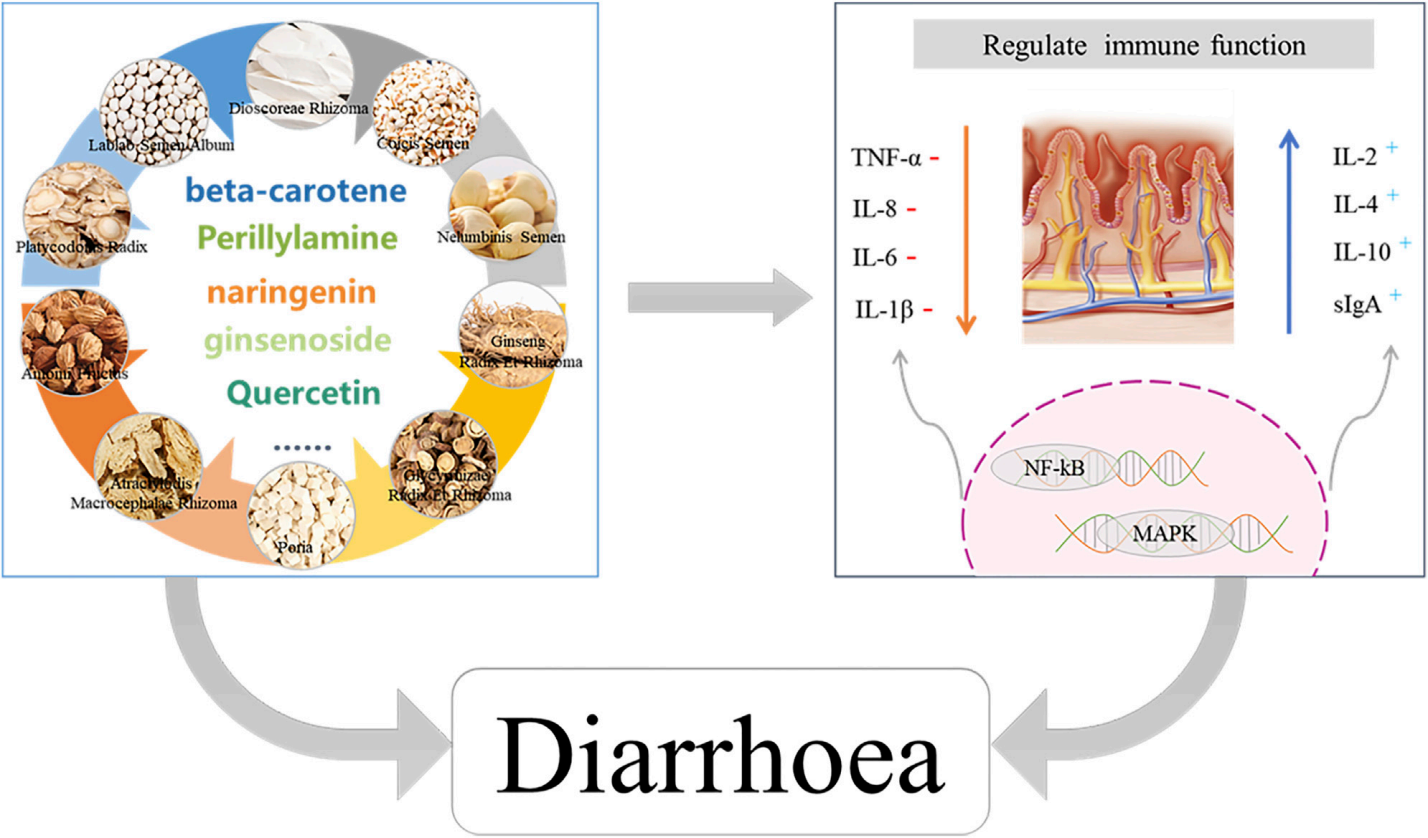
Mechanism of SLBZS in improving diarrhoea.

As a product consisting of a variety of plants with health benefits, SLBZS regulates immunity and improves the inflammatory response (Xiao et al., 2021). SLBZS contains ginsenosides, quercetin, lignan, β-sitosterol, β-carotene, kaempferol, origanoside D, rutin, atractylenolide III, poric acid, psoralen, iso-psoralen, iso-prenyl, and lobsteryl acetate (Lu et al.) (Supplementary Table S4). Luteolin in *Platycodonis radix* slows down the senescence of the spleen and thymus and acts directly on macrophages to reduce the expression of pro-inflammatory cytokines (Zhang et al., 2014), thereby enhancing immune action and playing an important role in maintaining the normal immune function of the body (Chen et al., 2006; Zhou et al., 2017). β-sitosterol in *Amomi fructus* extract increases the sIgA content of intestinal tissues, reduces the inflammatory response, and promotes immune function (Cheng et al., 2020). *A. fructus* also reduces IL-2 and TNF-α expression, possibly through the inhibition of the activation of the transcription factor nuclear factor-kappa B by its active ingredient, borneol acetate (Zhang et al., 2017). β-carotene in *White Lablab* modulates macrophage polarization and activates the JAK2/STAT3 and JNK/p38 MAPK signaling pathways to attenuate lipopolysaccharide-induced intestinal inflammation in rats (Lee et al., 2020; Yang et al., 2021). Ginsenosides, a compound in ginseng, exhibit anti-inflammatory effects by reducing the production of pro-inflammatory factors IL-1β, IL-6, IL-8, and TNF-α and increasing the levels of anti-inflammatory factors (Yao et al., 2019). The compound kaempferol found in *ginseng* and *Glycyrrhizae Radix et Rhizoma* also has good anti-inflammatory effects (Kitamura et al., 2018). Quercetin, a compound in *Semen Nelumbinis*, has been shown to exert anti-diarrheal effects in an animal study by improving histopathological changes in the small and large intestines through reduction of IL-1β, IL-6, and TNF- *a* levels in the small intestine (Zhang et al., 2008) (Figure 9). Overall, SLBZS has a complex and diverse composition, and there are many unexplored active ingredients that deserve in-depth study.

### 4.3 Limitations of the included studies

Owing to the limitations of the original studies, this systematic review, and meta-analysis had some limitations. First, there was selection bias; the original studies were confusing in their choice of indicator measures, and the consistency of outcome units was poor, reducing the mergeability of the findings. Second, as the included studies did not mention details of blinding and randomization, there was a potential risk of implementation bias. Third, some of the studies were derived from a series of reports by the same research group, and data were repeatedly reported, resulting in reporting bias and affecting the extrapolation of results. Additionally, all studies were conducted in China and published in Chinese, potentially causing language bias. Fourth, there was publication bias: Egger’s test found bias in the included studies, and the results remained unchanged after trim-and-fill analysis, suggesting that the quality of the original studies needs to be further checked at the source. Lastly, there were differences in the experimental design of the original studies. Firstly, although the included studies on SLBZS for diarrheal disease used rodents; there are genetic differences between rats and mice. Secondly, there are also no uniform standards for disease modeling methods, leading to differences in disease models. Thirdly, the samples selected for indicator testing differ (animal tissues, serum, plasma.), and there are differences in the selection of test kits and the assessment methods of the same indicators. The existence of these differences leads to a significant heterogeneity in the combined effect sizes. In addition to the lack of studies using exactly the same species of animals, disease models, and observed indicators, along with a grossly inadequate sample size for meta-analysis. These biases resulted in greater heterogeneity and bias in the combined results.

### 4.4 Implications for follow-up studies

SLBZS has been widely used clinically, however, the exact mechanism of its action remains unclear. This study, the first meta-analysis in this field, synthesizes current evidence from animal experiments related to the improvement of diarrhea by SLBZS through immunomodulation. Based on the deficiencies observed during our study, the following recommendations are made for future research. First, the importance of animal studies as a basis for preclinical applications cannot be overstated. The selection of animal models with a good match to the human body, as well as the standardization of disease modeling and evaluation methods, should receive more focus. Second, relevant manufacturers should standardize the production of SLBZS and promote the standardization of decoction method, treatment duration and dose in experiments. Third, follow-up studies should pay attention to the selection of disease endpoints and outcomes in accordance with accepted guidelines and expert consensus and to the standardized use of assays and reporting criteria. Lastly, the methodologies for animal studies and clinical trials are vastly different. The heterogeneity in most clinical data can be well addressed by sensitivity analysis or subgroup analysis, whereas that in animal studies cannot. Therefore, we strongly recommend that methodological specifications for animal studies are strengthened in the future and that evidence-based assessment models applicable to the characteristics of animal studies are created to facilitate the integration of preclinical data.

## 5 Conclusion

SLBZS can improve diarrhea with various causes by modulating immune and cell functions and reducing inflammation. It has immunomodulatory effects in rats and mice, and has inflammation-modulating effects in diarrheal diseases such as UC, CD, FD, IBS, and delayed diarrhea. The amount of effect varies between animal species and diseases. However, as the included studies did not mention details of blinding and randomization, their results could not be accurately evaluated, thus requiring higher-quality studies for verification.

Therefore, we strongly suggest that the methodological design of animal research be strengthened in future studies. Additionally, the selection of animal species, disease models, and dose and duration of drug intervention should be carried out in strict accordance with established guidelines and expert consensus. High-quality research design and more uniform and detailed reporting of results are required to confirm the evidence. Moreover, an evidence-based assessment system appropriate to the characteristics of animal studies should be established to facilitate the integration of preclinical data.

## Data Availability

The original contributions presented in the study are included in the article, and further inquiries can be directed to the corresponding authors. Requests to access these datasets should be directed to xiaofan122306@163.com.

## Abbreviations

CD, Crohn’s disease; CI, confidence interval; FD, functional diarrhea; GI, gastrointestinal; GRADE, Grading of Recommendations Assessment, Development, and Evaluation; IBD, irritable bowel syndrome; IL, interleukin; PRISMA, Preferred Reporting Items for Systematic Reviews and Meta-Analyses; CNKI, China National Knowledge Infrastructure; sIgA, secretory immunoglobulin; SLBZS, Shenling Baizhu powder; SMD, standardized mean differences; TNF-α, tumor necrosis factor alpha; UC, ulcerative colitis; RBC-C3b-RR, red blood cell c3b receptor ring rate; RBC-IC-RR, red blood cell immune complex ring rate.
