# Supplementary material for: Effect of Shenling Baizhu powder on immunity to diarrheal disease: A systematic review and meta-analysis
Source: Front Pharmacol. 2022 Sep 14;13:938932. doi: 10.3389/fphar.2022.938932 (PMC9516002; doi:10.3389/fphar.2022.938932)
Supplement: Supplementary file 2 [file DataSheet4.pdf]

Supplementary Figure

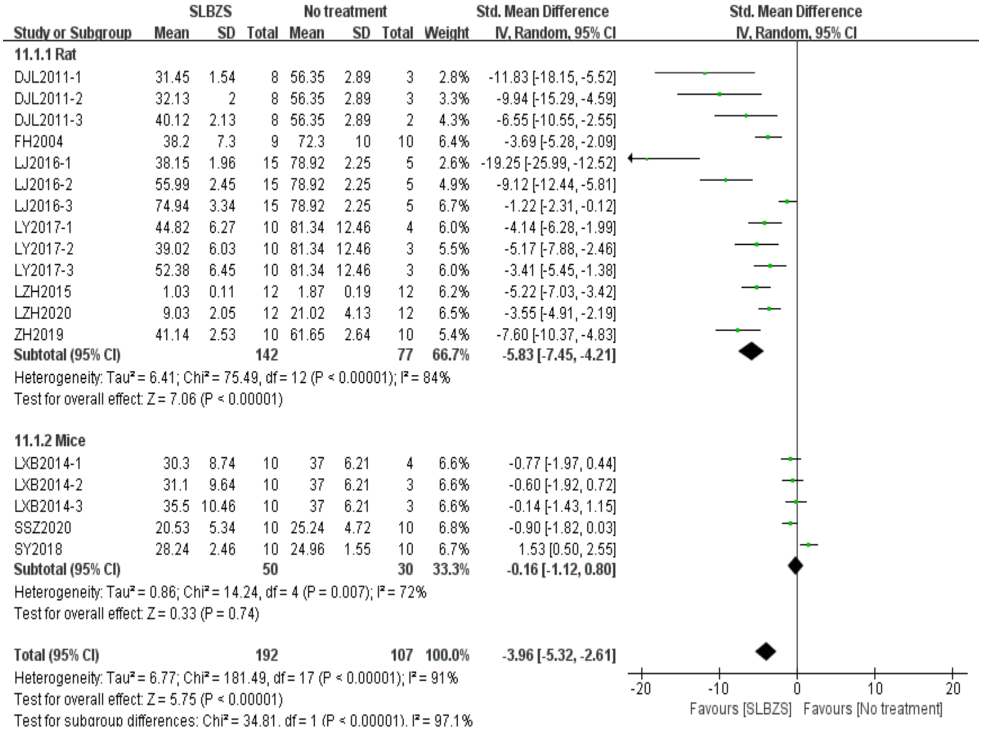

Supplementary figure S1 Subgroup analysis of TNF-α by animal species

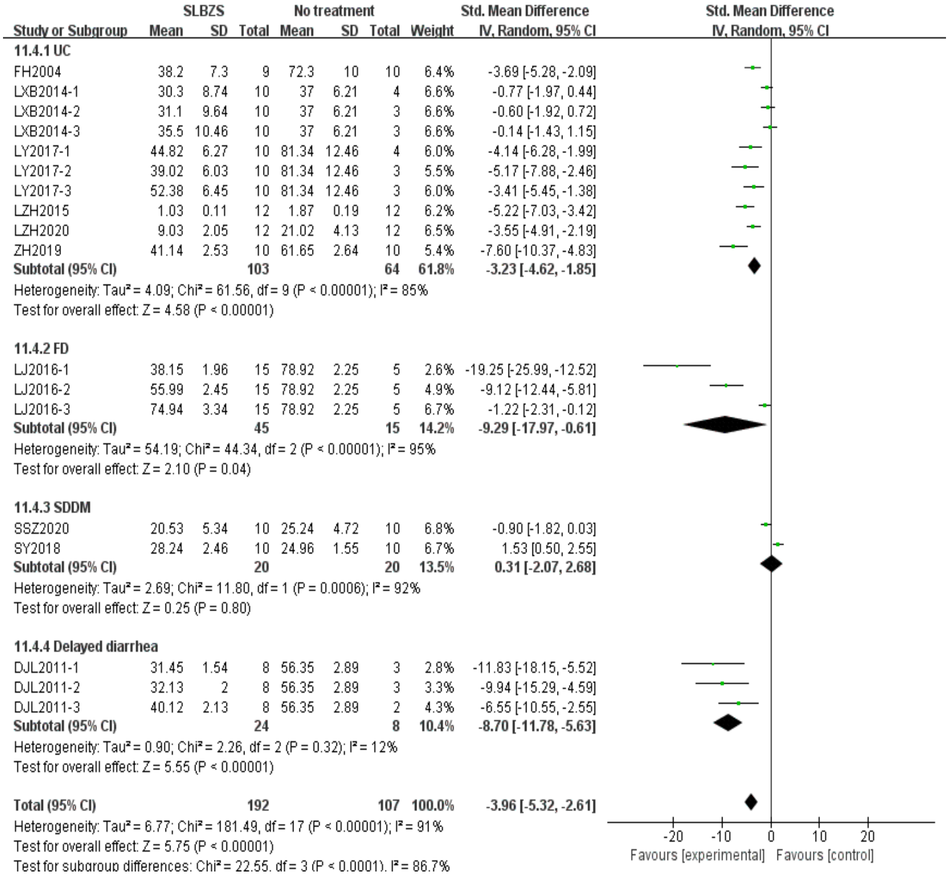

Supplementary figure S2 Subgroup analysis of TNF-α by disease model

Study or Subgroup	SLBZS Mean	SLBZS SD	SLBZS Total	No treatment Mean	No treatment SD	No treatment Total	Weight	Std. Mean Difference IV, Random, 95% CI
**11.2.1 <14d**								
DJL2011-1	31.45	1.54	8	56.35	2.89	3	2.8%	-11.83 [-18.15, -5.52]
DJL2011-2	32.13	2	8	56.35	2.89	3	3.3%	-9.94 [-15.29, -4.59]
DJL2011-3	40.12	2.13	8	56.35	2.89	2	4.3%	-6.55 [-10.55, -2.55]
LXB2014-1	30.3	8.74	10	37	6.21	4	6.6%	-0.77 [-1.97, 0.44]
LXB2014-2	31.1	9.64	10	37	6.21	3	6.6%	-0.60 [-1.92, 0.72]
LXB2014-3	35.5	10.46	10	37	6.21	3	6.6%	-0.14 [-1.43, 1.15]
SSZ2020	20.53	5.34	10	25.24	4.72	10	6.8%	-0.90 [-1.82, 0.03]
**Subtotal (95% CI)**			**64**			**28**	**37.0%**	**-2.28 [-3.85, -0.71]**
Heterogeneity: Tau² = 2.85; Chi² = 31.65, df = 6 (P < 0.0001); I² = 81%								
Test for overall effect: Z = 2.84 (P = 0.004)								
**11.2.2 ≥14d**								
FH2004	38.2	7.3	9	72.3	10	10	6.4%	-3.69 [-5.28, -2.09]
LJ2016-1	38.15	1.96	15	78.92	2.25	5	2.6%	-19.25 [-25.99, -12.52]
LJ2016-2	55.99	2.45	15	78.92	2.25	5	4.9%	-9.12 [-12.44, -5.81]
LJ2016-3	74.94	3.34	15	78.92	2.25	5	6.7%	-1.22 [-2.31, -0.12]
LY2017-1	44.82	6.27	10	81.34	12.46	4	6.0%	-4.14 [-6.28, -1.99]
LY2017-2	39.02	6.03	10	81.34	12.46	3	5.5%	-5.17 [-7.88, -2.46]
LY2017-3	52.38	6.45	10	81.34	12.46	3	6.0%	-3.41 [-5.45, -1.38]
LZH2015	1.03	0.11	12	1.87	0.19	12	6.2%	-5.22 [-7.03, -3.42]
LZH2020	9.03	2.05	12	21.02	4.13	12	6.5%	-3.55 [-4.91, -2.19]
SY2018	28.24	2.46	10	24.96	1.55	10	6.7%	1.53 [0.50, 2.55]
ZH2019	41.14	2.53	10	61.65	2.64	10	5.4%	-7.60 [-10.37, -4.83]
**Subtotal (95% CI)**			**128**			**79**	**63.0%**	**-4.73 [-6.75, -2.71]**
Heterogeneity: Tau² = 10.07; Chi² = 136.61, df = 10 (P < 0.00001); I² = 93%								
Test for overall effect: Z = 4.59 (P < 0.00001)								
**Total (95% CI)**			**192**			**107**	**100.0%**	**-3.96 [-5.32, -2.61]**
Heterogeneity: Tau² = 6.77; Chi² = 181.49, df = 17 (P < 0.00001); I² = 91%								
Test for overall effect: Z = 5.75 (P < 0.00001)								
Test for subgroup differences: Chi² = 3.53, df = 1 (P = 0.06), I² = 71.6%								

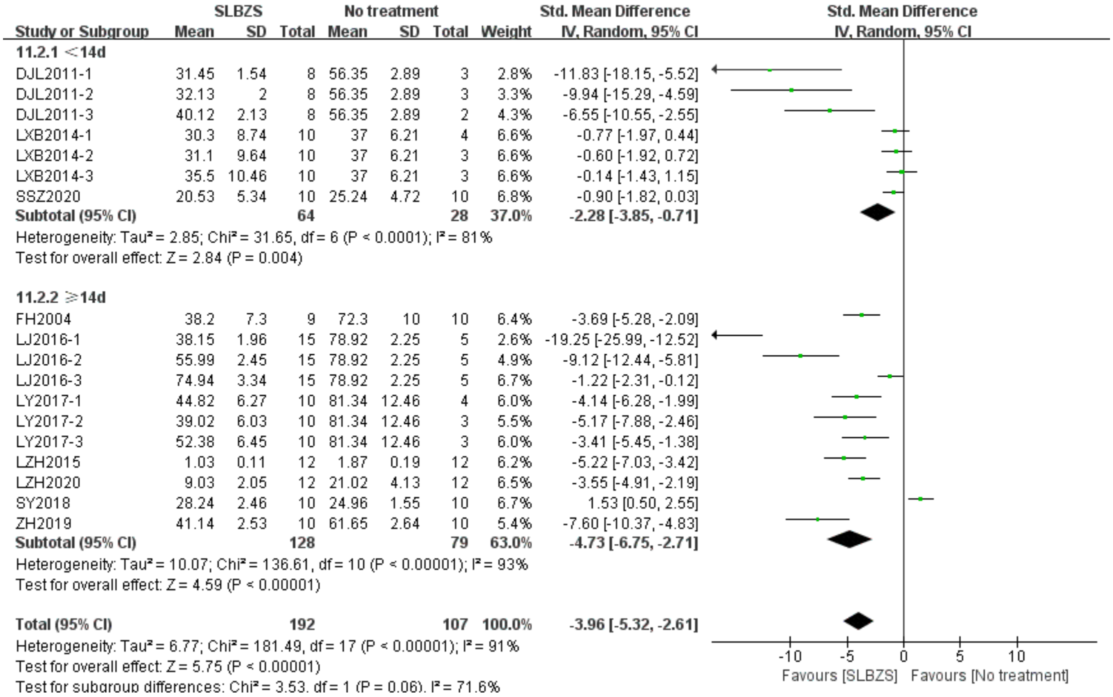

Supplementary figure S3 Subgroup analysis of TNF-α by different duration

Study or Subgroup	SLBZS Mean	SLBZS SD	SLBZS Total	No treatment Mean	No treatment SD	No treatment Total	Weight	Std. Mean Difference IV, Random, 95% CI
**11.3.1 ≤10g/kg**								
DJL2011-1	31.45	1.54	8	56.35	2.89	3	2.8%	-11.83 [-18.15, -5.52]
DJL2011-2	32.13	2	8	56.35	2.89	3	3.3%	-9.94 [-15.29, -4.59]
DJL2011-3	40.12	2.13	8	56.35	2.89	2	4.3%	-6.55 [-10.55, -2.55]
FH2004	38.2	7.3	9	72.3	10	10	6.4%	-3.69 [-5.28, -2.09]
LJ2016-1	38.15	1.96	15	78.92	2.25	5	2.6%	-19.25 [-25.99, -12.52]
LJ2016-2	55.99	2.45	15	78.92	2.25	5	4.9%	-9.12 [-12.44, -5.81]
LJ2016-3	74.94	3.34	15	78.92	2.25	5	6.7%	-1.22 [-2.31, -0.12]
LY2017-1	44.82	6.27	10	81.34	12.46	4	6.0%	-4.14 [-6.28, -1.99]
LY2017-2	39.02	6.03	10	81.34	12.46	3	5.5%	-5.17 [-7.88, -2.46]
LY2017-3	52.38	6.45	10	81.34	12.46	3	6.0%	-3.41 [-5.45, -1.38]
SSZ2020	20.53	5.34	10	25.24	4.72	10	6.8%	-0.90 [-1.82, 0.03]
**Subtotal (95% CI)**			**118**			**53**	**55.3%**	**-5.53 [-7.45, -3.61]**
Heterogeneity: Tau² = 7.78; Chi² = 84.41, df = 10 (P < 0.00001); I² = 88%								
Test for overall effect: Z = 5.65 (P < 0.00001)								
**11.3.2 10~20g/kg**								
LXB2014-3	35.5	10.46	10	37	6.21	3	6.6%	-0.14 [-1.43, 1.15]
LZH2015	1.03	0.11	12	1.87	0.19	12	6.2%	-5.22 [-7.03, -3.42]
LZH2020	9.03	2.05	12	21.02	4.13	12	6.5%	-3.55 [-4.91, -2.19]
SY2018	28.24	2.46	10	24.96	1.55	10	6.7%	1.53 [0.50, 2.55]
**Subtotal (95% CI)**			**44**			**37**	**26.1%**	**-1.79 [-4.75, 1.17]**
Heterogeneity: Tau² = 8.61; Chi² = 59.63, df = 3 (P < 0.00001); I² = 95%								
Test for overall effect: Z = 1.19 (P = 0.23)								
**11.3.3 >20g/kg**								
LXB2014-1	30.3	8.74	10	37	6.21	4	6.6%	-0.77 [-1.97, 0.44]
LXB2014-2	31.1	9.64	10	37	6.21	3	6.6%	-0.60 [-1.92, 0.72]
ZH2019	41.14	2.53	10	61.65	2.64	10	5.4%	-7.60 [-10.37, -4.83]
**Subtotal (95% CI)**			**30**			**17**	**18.6%**	**-2.68 [-5.73, 0.37]**
Heterogeneity: Tau² = 6.38; Chi² = 21.67, df = 2 (P < 0.0001); I² = 91%								
Test for overall effect: Z = 1.72 (P = 0.08)								
**Total (95% CI)**			**192**			**107**	**100.0%**	**-3.96 [-5.32, -2.61]**
Heterogeneity: Tau² = 6.77; Chi² = 181.49, df = 17 (P < 0.00001); I² = 91%								
Test for overall effect: Z = 5.75 (P < 0.00001)								
Test for subgroup differences: Chi² = 5.30, df = 2 (P = 0.07), I² = 62.2%								

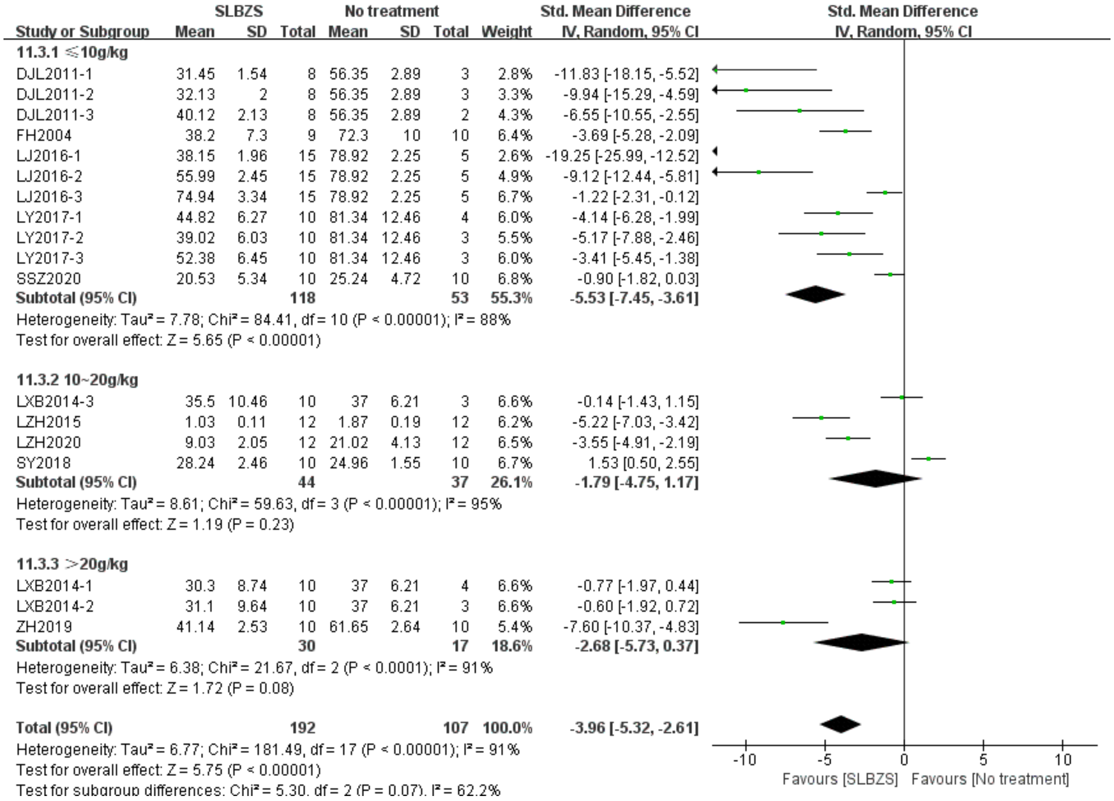

Supplementary figure S4 Subgroup analysis of TNF-α by different doses

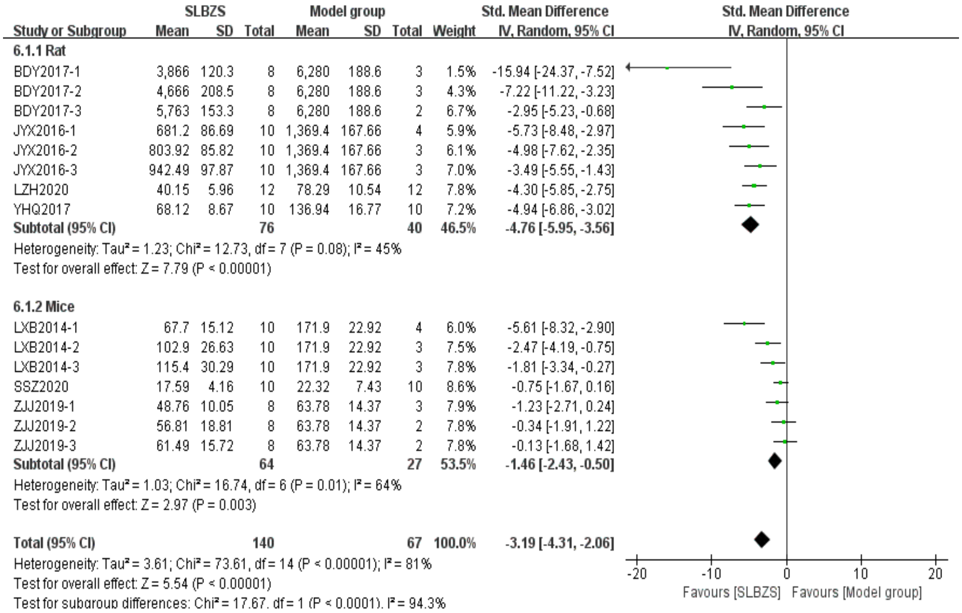

Supplementary figure S5 Subgroup analysis of IL-1β by animal species

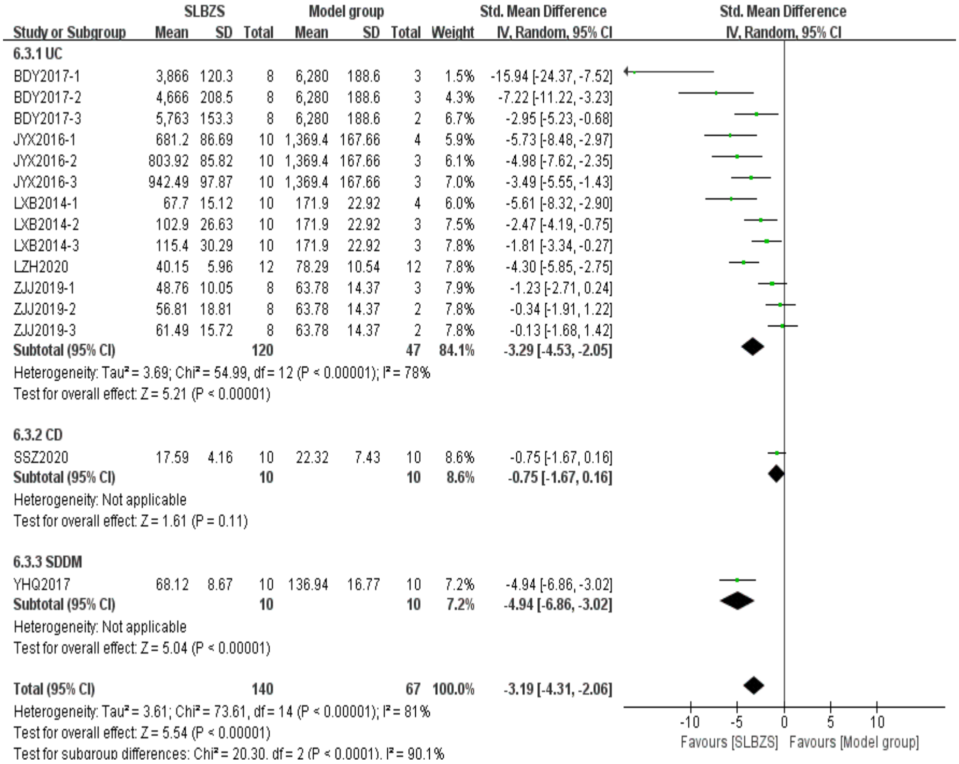

Supplementary figure S6 Subgroup analysis of IL-1β by disease model

Study or Subgroup	SLBZS Mean	SLBZS SD	SLBZS Total	No treatment Mean	No treatment SD	No treatment Total	Weight	Std. Mean Difference IV, Random, 95% CI
**6.2.1 <14d**								
BDY2017-1	3,866	120.3	8	6,280	188.6	3	1.5%	-15.94 [-24.37, -7.52]
BDY2017-2	4,666	208.5	8	6,280	188.6	3	4.3%	-7.22 [-11.22, -3.23]
BDY2017-3	5,763	153.3	8	6,280	188.6	2	6.7%	-2.95 [-5.23, -0.68]
JYX2016-1	681.2	86.69	10	1,369.4	167.66	4	5.9%	-5.73 [-8.48, -2.97]
JYX2016-2	803.92	85.82	10	1,369.4	167.66	3	6.1%	-4.98 [-7.62, -2.35]
JYX2016-3	942.49	97.87	10	1,369.4	167.66	3	7.0%	-3.49 [-5.55, -1.43]
LZH2020	40.15	5.96	12	78.29	10.54	12	7.8%	-4.30 [-5.85, -2.75]
YHQ2017	68.12	8.67	10	136.94	16.77	10	7.2%	-4.94 [-6.86, -3.02]
ZJJ2019-1	48.76	10.05	8	63.78	14.37	3	7.9%	-1.23 [-2.71, 0.24]
ZJJ2019-2	56.81	18.81	8	63.78	14.37	2	7.8%	-0.34 [-1.91, 1.22]
ZJJ2019-3	61.49	15.72	8	63.78	14.37	2	7.8%	-0.13 [-1.68, 1.42]
**Subtotal (95% CI)**			**100**			**47**	**70.0%**	**-3.61 [-5.12, -2.10]**
Heterogeneity: Tau² = 4.85; Chi² = 54.94, df = 10 (P < 0.00001); I² = 82%								
Test for overall effect: Z = 4.69 (P < 0.00001)								
**6.2.2 ≥14d**								
LXB2014-1	67.7	15.12	10	171.9	22.92	4	6.0%	-5.61 [-8.32, -2.90]
LXB2014-2	102.9	26.63	10	171.9	22.92	3	7.5%	-2.47 [-4.19, -0.75]
LXB2014-3	115.4	30.29	10	171.9	22.92	3	7.8%	-1.81 [-3.34, -0.27]
SSZ2020	17.59	4.16	10	22.32	7.43	10	8.6%	-0.75 [-1.67, 0.16]
**Subtotal (95% CI)**			**40**			**20**	**30.0%**	**-2.31 [-3.94, -0.68]**
Heterogeneity: Tau² = 2.01; Chi² = 12.73, df = 3 (P = 0.005); I² = 76%								
Test for overall effect: Z = 2.78 (P = 0.005)								
**Total (95% CI)**			**140**			**67**	**100.0%**	**-3.19 [-4.31, -2.06]**
Heterogeneity: Tau² = 3.61; Chi² = 73.61, df = 14 (P < 0.00001); I² = 81%								
Test for overall effect: Z = 5.54 (P < 0.00001)								
Test for subgroup differences: Chi² = 1.31, df = 1 (P = 0.25), I² = 23.8%								

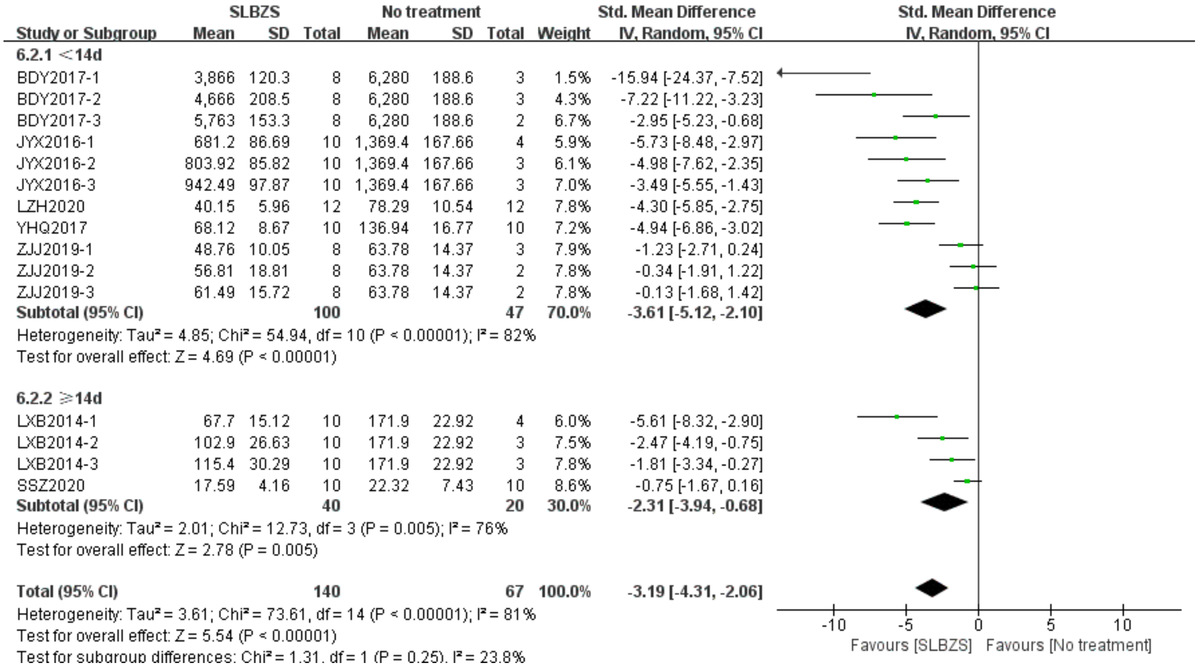

Supplementary figure S7 Subgroup analysis of IL-1β by in different duration

Study or Subgroup	SLBZS Mean	SLBZS SD	SLBZS Total	No treatment Mean	No treatment SD	No treatment Total	Weight	Std. Mean Difference IV, Random, 95% CI
**6.4.1 <10g/kg**								
BDY2017-3	5,763	153.3	8	6,280	188.6	2	6.7%	-2.95 [-5.23, -0.68]
JYX2016-3	942.49	97.87	10	1,369.4	167.66	3	7.0%	-3.49 [-5.55, -1.43]
SSZ2020	17.59	4.16	10	22.32	7.43	10	8.6%	-0.75 [-1.67, 0.16]
ZJJ2019-3	61.49	15.72	8	63.78	14.37	2	7.8%	-0.13 [-1.68, 1.42]
**Subtotal (95% CI)**			**36**			**17**	**30.1%**	**-1.61 [-3.05, -0.16]**
Heterogeneity: Tau² = 1.45; Chi² = 9.75, df = 3 (P = 0.02); I² = 69%								
Test for overall effect: Z = 2.17 (P = 0.03)								
**6.4.2 10~20g/kg**								
BDY2017-2	4,666	208.5	8	6,280	188.6	3	4.3%	-7.22 [-11.22, -3.23]
JYX2016-2	803.92	85.82	10	1,369.4	167.66	3	6.1%	-4.98 [-7.62, -2.35]
LXB2014-3	115.4	30.29	10	171.9	22.92	3	7.8%	-1.81 [-3.34, -0.27]
LZH2020	40.15	5.96	12	78.29	10.54	12	7.8%	-4.30 [-5.85, -2.75]
YHQ2017	68.12	8.67	10	136.94	16.77	10	7.2%	-4.94 [-6.86, -3.02]
ZJJ2019-2	56.81	18.81	8	63.78	14.37	2	7.8%	-0.34 [-1.91, 1.22]
**Subtotal (95% CI)**			**58**			**33**	**41.0%**	**-3.62 [-5.46, -1.77]**
Heterogeneity: Tau² = 4.09; Chi² = 26.52, df = 5 (P < 0.0001); I² = 81%								
Test for overall effect: Z = 3.83 (P = 0.0001)								
**6.4.3 ≥20g/kg**								
BDY2017-1	3,866	120.3	8	6,280	188.6	3	1.5%	-15.94 [-24.37, -7.52]
JYX2016-1	681.2	86.69	10	1,369.4	167.66	4	5.9%	-5.73 [-8.48, -2.97]
LXB2014-1	67.7	15.12	10	171.9	22.92	4	6.0%	-5.61 [-8.32, -2.90]
LXB2014-2	102.9	26.63	10	171.9	22.92	3	7.5%	-2.47 [-4.19, -0.75]
ZJJ2019-1	48.76	10.05	8	63.78	14.37	3	7.9%	-1.23 [-2.71, 0.24]
**Subtotal (95% CI)**			**46**			**17**	**28.9%**	**-4.47 [-7.08, -1.87]**
Heterogeneity: Tau² = 6.38; Chi² = 22.24, df = 4 (P = 0.0002); I² = 82%								
Test for overall effect: Z = 3.36 (P = 0.0008)								
**Total (95% CI)**			**140**			**67**	**100.0%**	**-3.19 [-4.31, -2.06]**
Heterogeneity: Tau² = 3.61; Chi² = 73.61, df = 14 (P < 0.00001); I² = 81%								
Test for overall effect: Z = 5.54 (P < 0.00001)								
Test for subgroup differences: Chi² = 4.91, df = 2 (P = 0.09), I² = 59.3%								

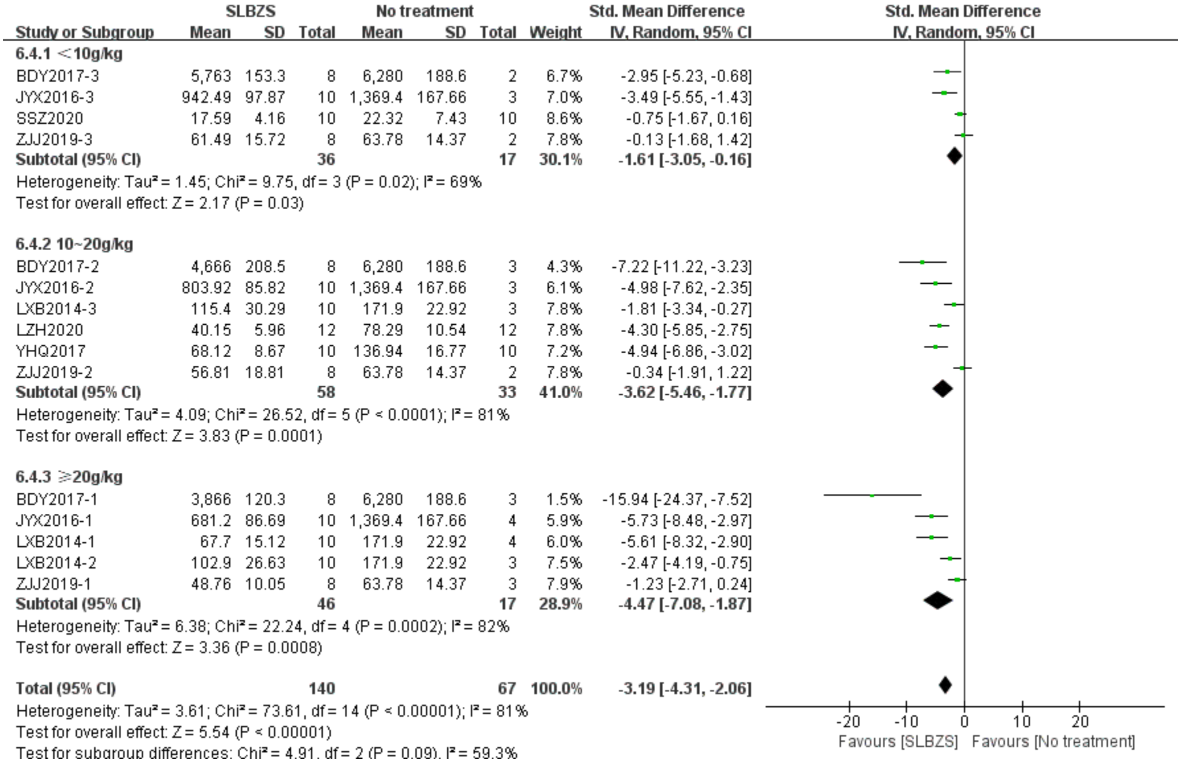

Supplementary figure S8 Subgroup analysis of IL-1β by different doses

Study or Subgroup	SLBZS Mean	SLBZS SD	SLBZS Total	No treatment Mean	No treatment SD	No treatment Total	Weight	Std. Mean Difference IV, Random, 95% CI
**9.2.1 <14d**								
DJL2011-1	800.56	38.23	8	1,459.92	74.92	3	2.1%	-12.35 [-18.92, -5.77]
DJL2011-2	900.23	45.34	8	1,459.92	74.92	3	3.0%	-9.59 [-14.77, -4.42]
DJL2011-3	1,104.52	56.12	8	1,459.92	74.92	2	5.0%	-5.46 [-8.90, -2.02]
**Subtotal (95% CI)**			**24**			**8**	**10.0%**	**-8.42 [-12.46, -4.37]**
Heterogeneity: Tau² = 6.50; Chi² = 4.06, df = 2 (P = 0.13); I² = 51%								
Test for overall effect: Z = 4.08 (P < 0.0001)								
**9.2.2 ≥14d**								
FH2004	136.8	12.2	9	186.8	15.3	10	8.7%	-3.43 [-4.95, -1.91]
LY2017-1	126.35	11.32	10	219.21	18.6	4	5.6%	-6.43 [-9.47, -3.39]
LY2017-2	121.12	11.07	10	219.21	18.6	3	4.8%	-7.14 [-10.68, -3.61]
LY2017-3	134.14	13.61	10	219.21	18.6	3	6.1%	-5.40 [-8.21, -2.60]
LZH2015	29.01	3.28	12	39.64	4.23	12	9.4%	-2.71 [-3.87, -1.55]
LZH2020	18.24	2.48	12	32.26	5.36	12	9.2%	-3.24 [-4.52, -1.96]
TJS2021-1	128.37	12.74	12	235.64	16.46	6	5.9%	-7.29 [-10.16, -4.42]
TJS2021-2	170.03	11.18	12	235.64	16.46	6	7.6%	-4.78 [-6.81, -2.76]
YHQ2018-1	88.35	7.05	10	131.97	6.29	4	6.0%	-5.95 [-8.79, -3.10]
YHQ2018-2	106.73	12.16	10	131.97	6.29	3	8.5%	-2.07 [-3.68, -0.47]
YHQ2018-3	115.58	7.94	10	131.97	6.29	3	8.5%	-1.99 [-3.57, -0.41]
ZH2019	3,623.75	95.16	10	3,777.5	97.06	10	9.6%	-1.53 [-2.56, -0.51]
**Subtotal (95% CI)**			**127**			**76**	**90.0%**	**-3.84 [-4.84, -2.85]**
Heterogeneity: Tau² = 2.01; Chi² = 40.07, df = 11 (P < 0.0001); I² = 73%								
Test for overall effect: Z = 7.56 (P < 0.00001)								
**Total (95% CI)**			**151**			**84**	**100.0%**	**-4.35 [-5.40, -3.29]**
Heterogeneity: Tau² = 2.74; Chi² = 55.31, df = 14 (P < 0.00001); I² = 75%								
Test for overall effect: Z = 8.07 (P < 0.00001)								
Test for subgroup differences: Chi² = 4.64, df = 1 (P = 0.03), I² = 78.4%								

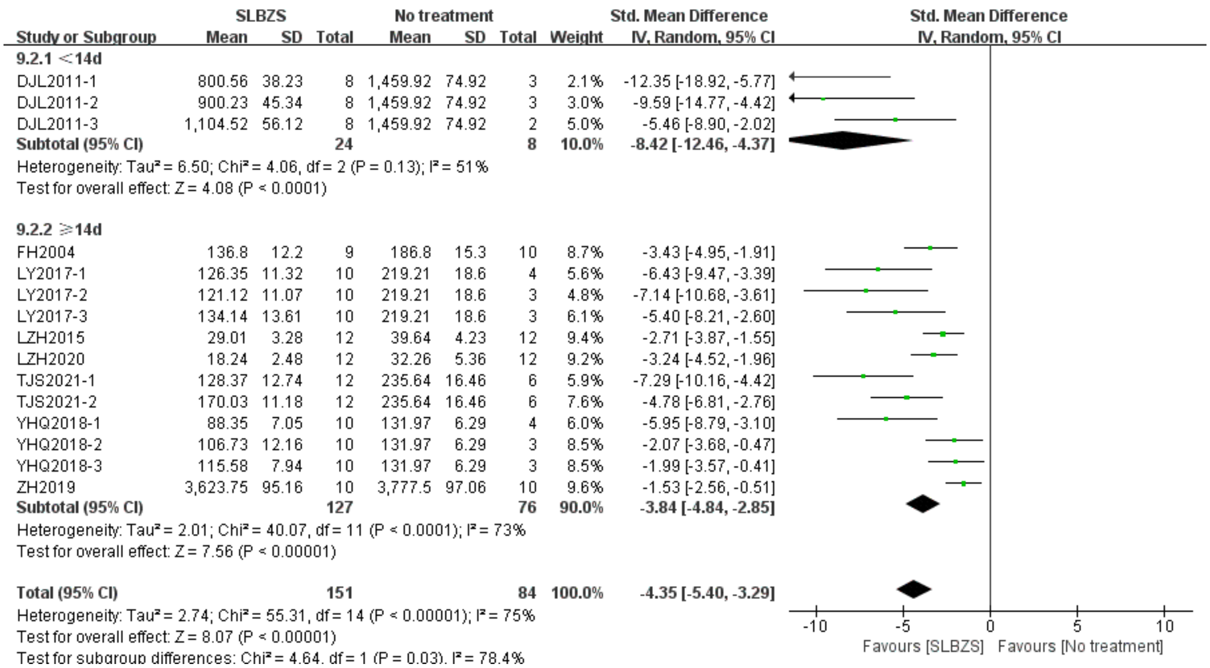

Supplementary figure S9 Subgroup analysis of IL-6 by disease model

Study or Subgroup	SLBZS Mean	SLBZS SD	SLBZS Total	Model group Mean	Model group SD	Model group Total	Weight	Std. Mean Difference IV, Random, 95% CI
**9.4.1 UC**								
FH2004	136.8	12.2	9	186.8	15.3	10	8.7%	-3.43 [-4.95, -1.91]
LY2017-1	126.35	11.32	10	219.21	18.6	4	5.6%	-6.43 [-9.47, -3.39]
LY2017-2	121.12	11.07	10	219.21	18.6	3	4.8%	-7.14 [-10.68, -3.61]
LY2017-3	134.14	13.61	10	219.21	18.6	3	6.1%	-5.40 [-8.21, -2.60]
LZH2015	29.01	3.28	12	39.64	4.23	12	9.4%	-2.71 [-3.87, -1.55]
LZH2020	18.24	2.48	12	32.26	5.36	12	9.2%	-3.24 [-4.52, -1.96]
TJS2021-1	128.37	12.74	12	235.64	16.46	6	5.9%	-7.29 [-10.16, -4.42]
TJS2021-2	170.03	11.18	12	235.64	16.46	6	7.6%	-4.78 [-6.81, -2.76]
YHQ2018-1	88.35	7.05	10	131.97	6.29	4	6.0%	-5.95 [-8.79, -3.10]
YHQ2018-2	106.73	12.16	10	131.97	6.29	3	8.5%	-2.07 [-3.68, -0.47]
YHQ2018-3	115.58	7.94	10	131.97	6.29	3	8.5%	-1.99 [-3.57, -0.41]
ZH2019	3,623.75	95.16	10	3,777.5	97.06	10	9.6%	-1.53 [-2.56, -0.51]
**Subtotal (95% CI)**			**127**			**76**	**90.0%**	**-3.84 [-4.84, -2.85]**
Heterogeneity: Tau² = 2.01; Chi² = 40.07, df = 11 (P < 0.0001); I² = 73%								
Test for overall effect: Z = 7.56 (P < 0.00001)								
**9.4.2 Delayed Diarrhea**								
DJL2011-1	800.56	38.23	8	1,459.92	74.92	3	2.1%	-12.35 [-18.92, -5.77]
DJL2011-2	900.23	45.34	8	1,459.92	74.92	3	3.0%	-9.59 [-14.77, -4.42]
DJL2011-3	1,104.52	56.12	8	1,459.92	74.92	2	5.0%	-5.46 [-8.90, -2.02]
**Subtotal (95% CI)**			**24**			**8**	**10.0%**	**-8.42 [-12.46, -4.37]**
Heterogeneity: Tau² = 6.50; Chi² = 4.06, df = 2 (P = 0.13); I² = 51%								
Test for overall effect: Z = 4.08 (P < 0.0001)								
**Total (95% CI)**			**151**			**84**	**100.0%**	**-4.35 [-5.40, -3.29]**
Heterogeneity: Tau² = 2.74; Chi² = 55.31, df = 14 (P < 0.00001); I² = 75%								
Test for overall effect: Z = 8.07 (P < 0.00001)								
Test for subgroup differences: Chi² = 4.64, df = 1 (P = 0.03), I² = 78.4%								

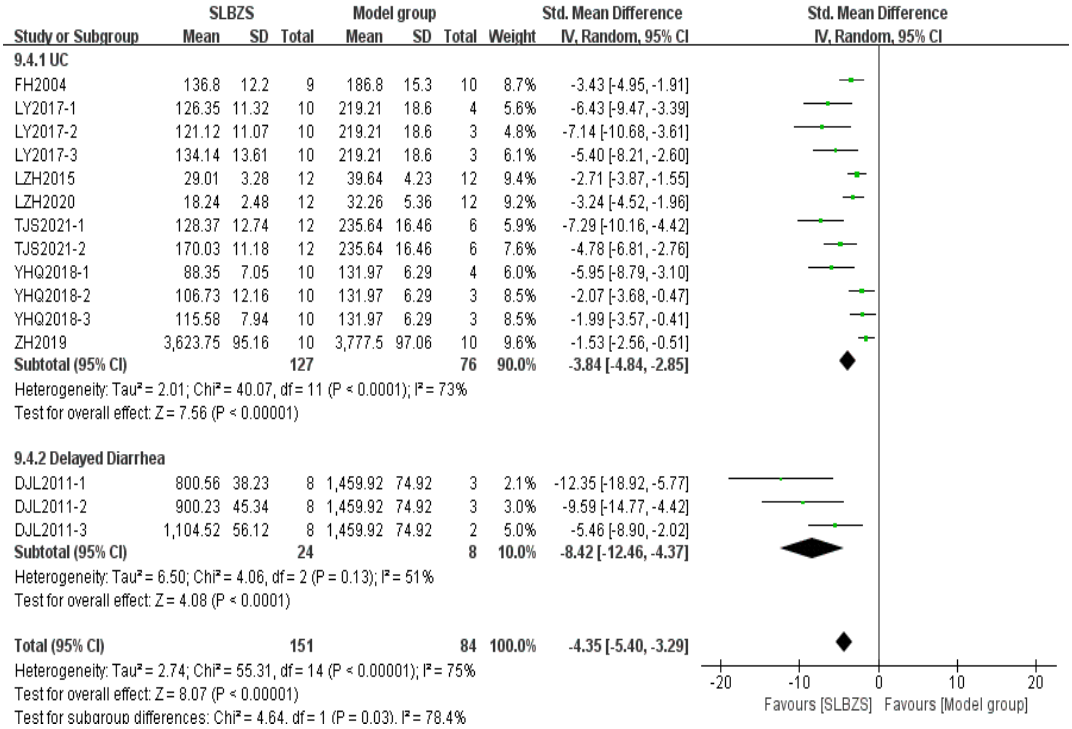

Supplementary figure S10 Subgroup analysis of IL-6 by different duration

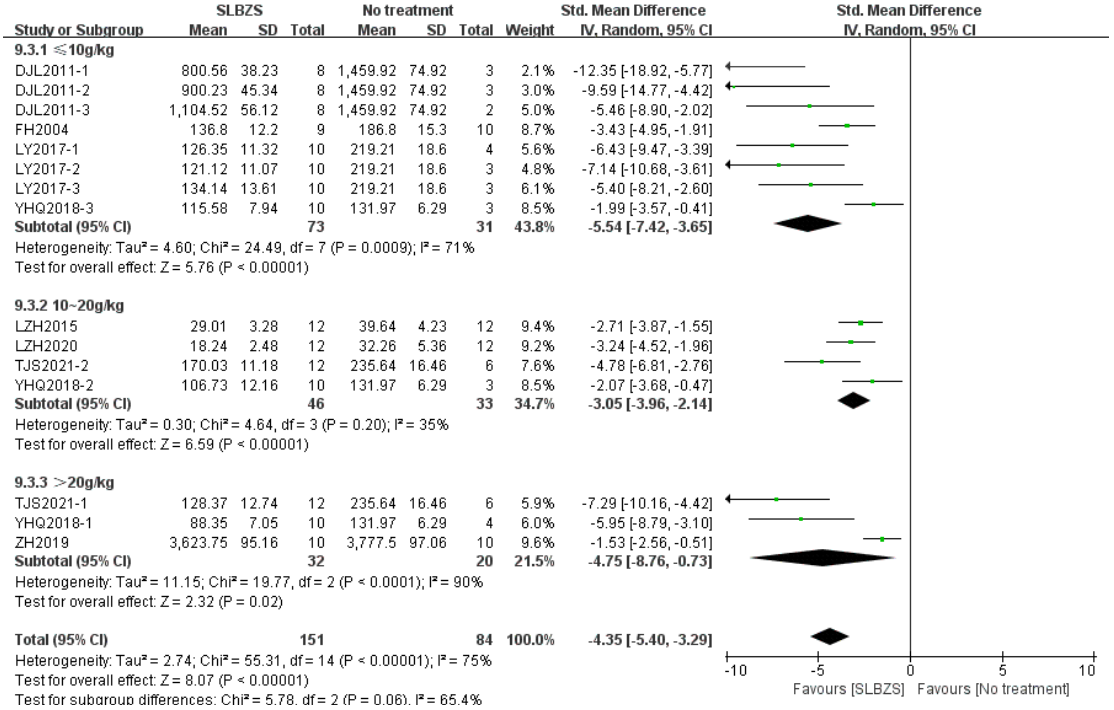

Supplementary figure S11 Subgroup analysis of IL-6 by different doses

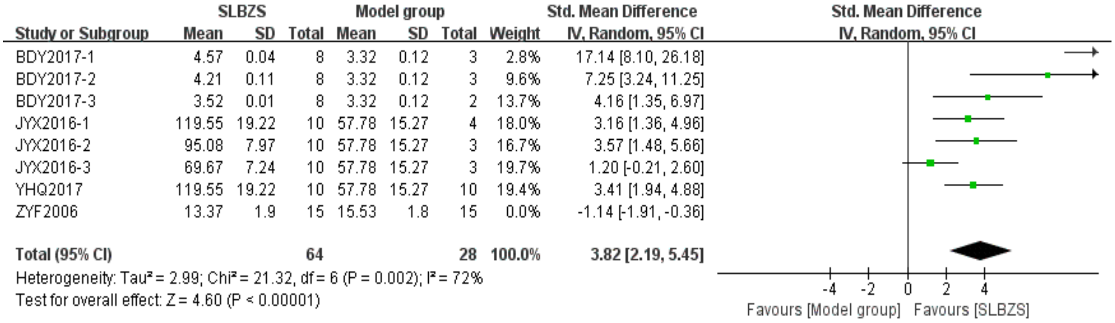

Supplementary figure S12 The results of IL-4.

Study or Subgroup	SLBZS Mean	SLBZS SD	SLBZS Total	Model group Mean	Model group SD	Model group Total	Weight	Std. Mean Difference IV, Random, 95% CI
**8.1.1 Rat**								
DJL2011-1	346.09	21.31	8	124.12	11.23	3	1.6%	10.39 [4.81, 15.98]
DJL2011-2	308.25	19.47	8	124.12	11.23	3	1.8%	9.37 [4.31, 14.43]
DJL2011-3	204.38	15.62	8	124.12	11.23	2	3.4%	4.79 [1.68, 7.90]
FH2004	60.2	4.8	9	40.7	7.1	10	6.0%	3.04 [1.63, 4.45]
LY2017-1	88.76	8.73	10	51.62	9.62	4	4.9%	3.88 [1.83, 5.93]
LY2017-2	93.07	9.04	10	51.62	9.62	3	4.5%	4.21 [1.88, 6.55]
LY2017-3	85.06	8.62	10	51.62	9.62	3	4.9%	3.53 [1.46, 5.61]
TJS2021-1	178.29	13.25	12	93.28	10.83	6	4.1%	6.45 [3.88, 9.03]
TJS2021-2	148.63	16.58	12	93.28	10.83	6	5.7%	3.51 [1.88, 5.13]
YHQ2017	112.48	11.99	10	83.35	5.15	10	6.1%	3.02 [1.66, 4.39]
YHQ2018-1	127.72	10.07	10	69.75	8.53	4	4.0%	5.59 [2.89, 8.29]
YHQ2018-2	95.41	5.41	10	69.75	8.53	3	4.7%	3.91 [1.70, 6.13]
YHQ2018-3	82.83	5.54	10	69.75	8.53	3	5.7%	1.97 [0.39, 3.54]
ZH2019	425	10.69	10	343.75	5.82	10	3.3%	9.04 [5.79, 12.29]
**Subtotal (95% CI)**			**137**			**70**	**60.8%**	**4.41 [3.44, 5.38]**
Heterogeneity: Tau² = 1.89; Chi² = 34.04, df = 13 (P = 0.001); I² = 62%								
Test for overall effect: Z = 8.92 (P < 0.00001)								
**8.1.2 Mice**								
LXB2014-1	187.74	36.94	10	75.61	12.34	4	5.3%	3.22 [1.40, 5.05]
LXB2014-2	158.42	15.75	10	75.61	12.34	3	4.0%	5.07 [2.40, 7.74]
LXB2014-3	91.28	24.89	10	75.61	12.34	3	6.2%	0.63 [-0.69, 1.95]
YY2019-1	353.66	40.103	12	196.63	36.3	4	5.2%	3.78 [1.89, 5.66]
YY2019-2	315.37	67.52	12	196.63	36.3	4	6.1%	1.81 [0.46, 3.15]
YY2019-3	293.3	67.31	12	196.63	36.3	4	6.2%	1.47 [0.20, 2.75]
ZYF2006	34.72	3.1	15	22.36	2.7	15	6.1%	4.14 [2.81, 5.47]
**Subtotal (95% CI)**			**81**			**37**	**39.2%**	**2.70 [1.54, 3.86]**
Heterogeneity: Tau² = 1.74; Chi² = 22.86, df = 6 (P = 0.0008); I² = 74%								
Test for overall effect: Z = 4.57 (P < 0.00001)								
**Total (95% CI)**			**218**			**107**	**100.0%**	**3.77 [2.98, 4.56]**
Heterogeneity: Tau² = 2.17; Chi² = 68.66, df = 20 (P < 0.00001); I² = 71%								
Test for overall effect: Z = 9.38 (P < 0.00001)								
Test for subgroup differences: Chi² = 4.91, df = 1 (P = 0.03), I² = 79.6%								

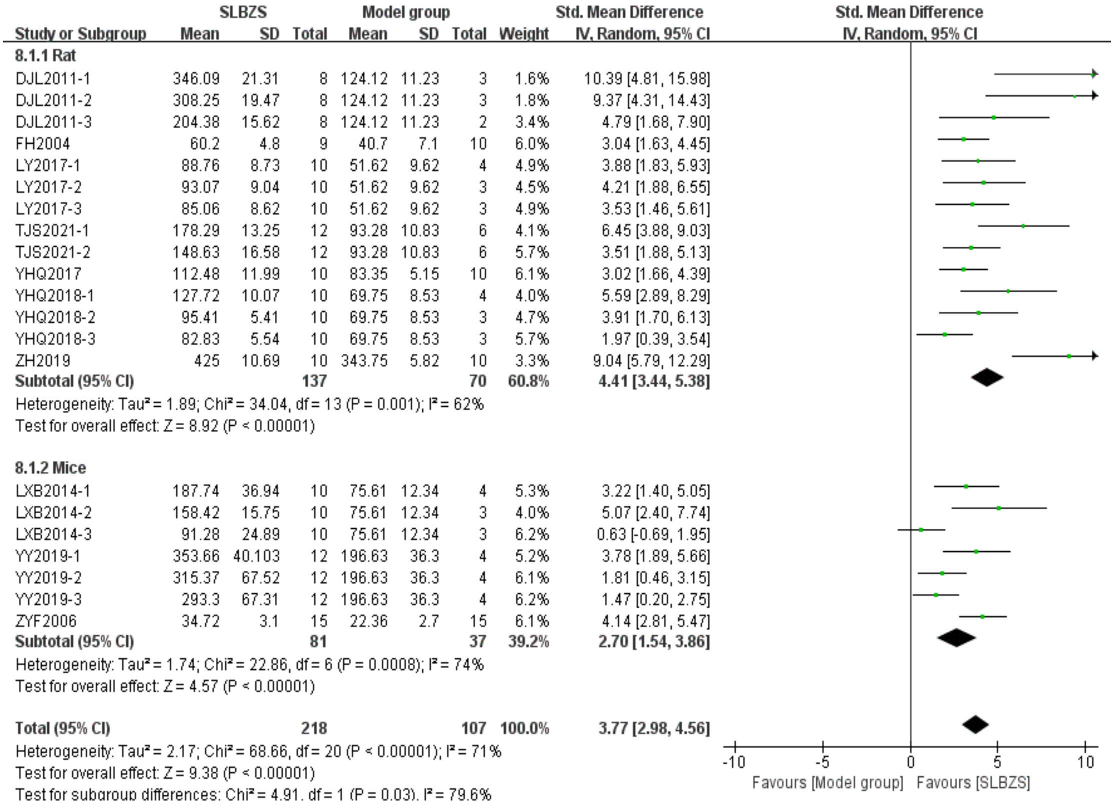

Supplementary figure S13 Subgroup analysis of IL-10 by animal species.

Study or Subgroup	SLBZS Mean	SLBZS SD	SLBZS Total	Model group Mean	Model group SD	Model group Total	Weight	Std. Mean Difference IV, Random, 95% CI
**8.4.1 UC**								
FH2004	60.2	4.8	9	40.7	7.1	10	6.0%	3.04 [1.63, 4.45]
LXB2014-1	187.74	36.94	10	75.61	12.34	4	5.3%	3.22 [1.40, 5.05]
LXB2014-2	158.42	15.75	10	75.61	12.34	3	4.0%	5.07 [2.40, 7.74]
LXB2014-3	91.28	24.89	10	75.61	12.34	3	6.2%	0.63 [-0.69, 1.95]
LY2017-1	88.76	8.73	10	51.62	9.62	4	4.9%	3.88 [1.83, 5.93]
LY2017-2	93.07	9.04	10	51.62	9.62	3	4.5%	4.21 [1.88, 6.55]
LY2017-3	85.06	8.62	10	51.62	9.62	3	4.9%	3.53 [1.46, 5.61]
TJS2021-1	178.29	13.25	12	93.28	10.83	6	4.1%	6.45 [3.88, 9.03]
TJS2021-2	148.63	16.58	12	93.28	10.83	6	5.7%	3.51 [1.88, 5.13]
YHQ2018-1	127.72	10.07	10	69.75	8.53	4	4.0%	5.59 [2.89, 8.29]
YHQ2018-2	95.41	5.41	10	69.75	8.53	3	4.7%	3.91 [1.70, 6.13]
YHQ2018-3	82.83	5.54	10	69.75	8.53	3	5.7%	1.97 [0.39, 3.54]
ZH2019	425	10.69	10	343.75	5.82	10	3.3%	9.04 [5.79, 12.29]
**Subtotal (95% CI)**			**133**			**62**	**63.3%**	**3.86 [2.83, 4.90]**
Heterogeneity: Tau² = 2.45; Chi² = 42.05, df = 12 (P < 0.0001); I² = 71%								
Test for overall effect: Z = 7.33 (P < 0.00001)								
**8.4.2 IBD**								
YY2019-1	353.66	40.103	12	196.63	36.3	4	5.2%	3.78 [1.89, 5.66]
YY2019-2	315.37	67.52	12	196.63	36.3	4	6.1%	1.81 [0.46, 3.15]
YY2019-3	293.3	67.31	12	196.63	36.3	4	6.2%	1.47 [0.20, 2.75]
**Subtotal (95% CI)**			**36**			**12**	**17.6%**	**2.18 [0.96, 3.41]**
Heterogeneity: Tau² = 0.60; Chi² = 4.13, df = 2 (P = 0.13); I² = 52%								
Test for overall effect: Z = 3.50 (P = 0.0005)								
**8.4.3 CD**								
YHQ2017	112.48	11.99	10	83.35	5.15	10	6.1%	3.02 [1.66, 4.39]
**Subtotal (95% CI)**			**10**			**10**	**6.1%**	**3.02 [1.66, 4.39]**
Heterogeneity: Not applicable								
Test for overall effect: Z = 4.34 (P < 0.0001)								
**8.4.4 Delayed diarrhea**								
DJL2011-1	346.09	21.31	8	124.12	11.23	3	1.6%	10.39 [4.81, 15.98]
DJL2011-2	308.25	19.47	8	124.12	11.23	3	1.8%	9.37 [4.31, 14.43]
DJL2011-3	204.38	15.62	8	124.12	11.23	2	3.4%	4.79 [1.68, 7.90]
**Subtotal (95% CI)**			**24**			**8**	**6.8%**	**7.62 [3.86, 11.37]**
Heterogeneity: Tau² = 5.78; Chi² = 4.19, df = 2 (P = 0.12); I² = 52%								
Test for overall effect: Z = 3.98 (P < 0.0001)								
**8.4.5 Colitis**								
ZYF2006	34.72	3.1	15	22.36	2.7	15	6.1%	4.14 [2.81, 5.47]
**Subtotal (95% CI)**			**15**			**15**	**6.1%**	**4.14 [2.81, 5.47]**
Heterogeneity: Not applicable								
Test for overall effect: Z = 6.09 (P < 0.00001)								
**Total (95% CI)**			**218**			**107**	**100.0%**	**3.77 [2.98, 4.56]**
Heterogeneity: Tau² = 2.17; Chi² = 68.66, df = 20 (P < 0.00001); I² = 71%								
Test for overall effect: Z = 9.38 (P < 0.00001)								
Test for subgroup differences: Chi² = 10.87, df = 4 (P = 0.03), I² = 63.2%								

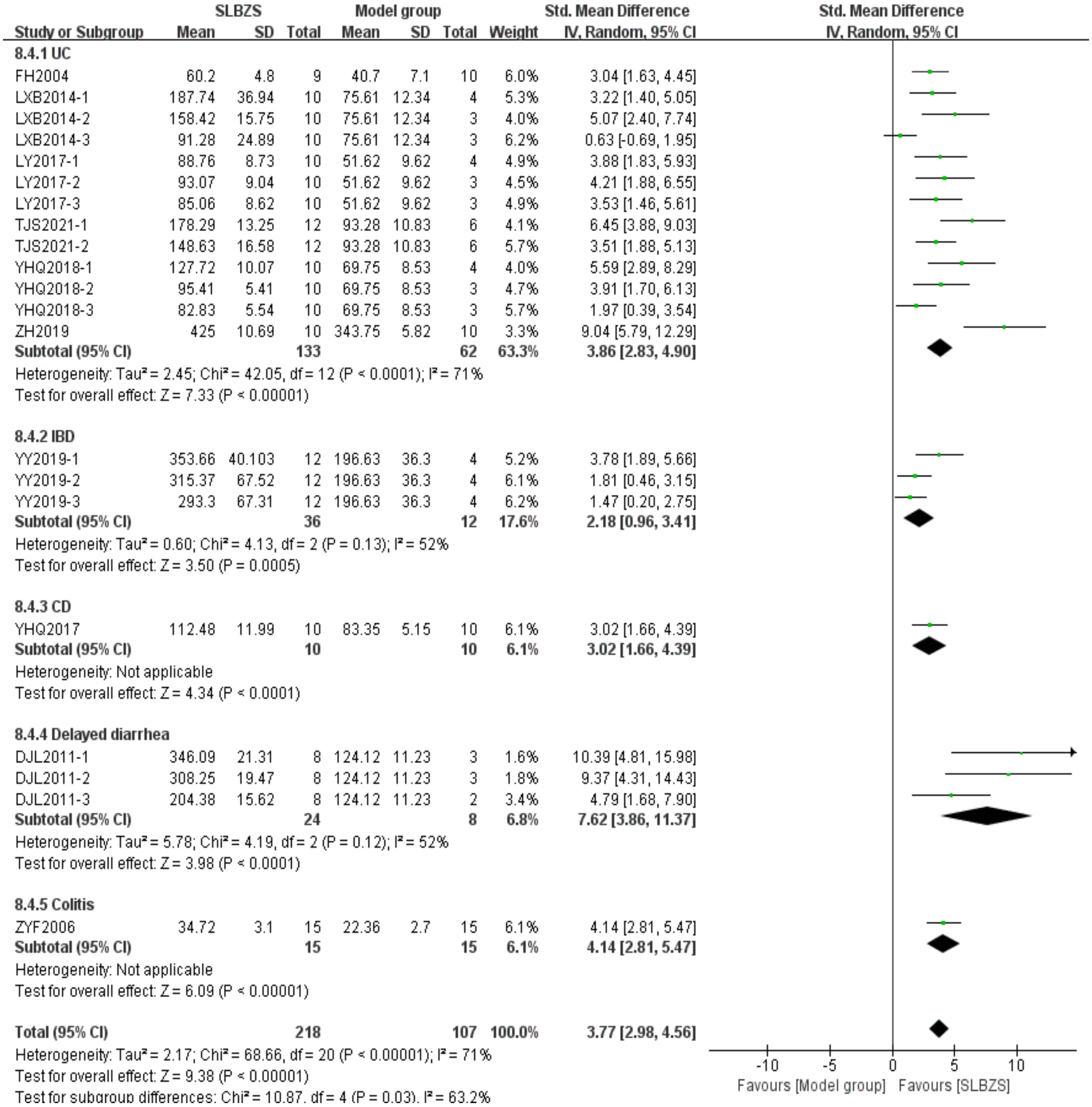

Supplementary figure S14 Subgroup analysis of IL-10 by disease model.

Study or Subgroup	SLBZS Mean	SLBZS SD	SLBZS Total	No treatment Mean	No treatment SD	No treatment Total	Weight	Std. Mean Difference IV, Random, 95% CI
**8.2.1 <14d**								
DJL2011-1	346.09	21.31	8	124.12	11.23	3	1.6%	10.39 [4.81, 15.98]
DJL2011-2	308.25	19.47	8	124.12	11.23	3	1.8%	9.37 [4.31, 14.43]
DJL2011-3	204.38	15.62	8	124.12	11.23	2	3.4%	4.79 [1.68, 7.90]
LXB2014-1	187.74	36.94	10	75.61	12.34	4	5.3%	3.22 [1.40, 5.05]
LXB2014-2	158.42	15.75	10	75.61	12.34	3	4.0%	5.07 [2.40, 7.74]
LXB2014-3	91.28	24.89	10	75.61	12.34	3	6.2%	0.63 [-0.69, 1.95]
ZYF2006	34.72	3.1	15	22.36	2.7	15	6.1%	4.14 [2.81, 5.47]
**Subtotal (95% CI)**			**69**			**33**	**28.5%**	**4.50 [2.51, 6.48]**
Heterogeneity: Tau² = 5.03; Chi² = 31.38, df = 6 (P < 0.0001); I² = 81%								
Test for overall effect: Z = 4.44 (P < 0.00001)								
**8.2.2 ≥14d**								
FH2004	60.2	4.8	9	40.7	7.1	10	6.0%	3.04 [1.63, 4.45]
LY2017-1	88.76	8.73	10	51.62	9.62	4	4.9%	3.88 [1.83, 5.93]
LY2017-2	93.07	9.04	10	51.62	9.62	3	4.5%	4.21 [1.88, 6.55]
LY2017-3	85.06	8.62	10	51.62	9.62	3	4.9%	3.53 [1.46, 5.61]
TJS2021-1	178.29	13.25	12	93.28	10.83	6	4.1%	6.45 [3.88, 9.03]
TJS2021-2	148.63	16.58	12	93.28	10.83	6	5.7%	3.51 [1.88, 5.13]
YHQ2017	112.48	11.99	10	83.35	5.15	10	6.1%	3.02 [1.66, 4.39]
YHQ2018-1	127.72	10.07	10	69.75	8.53	4	4.0%	5.59 [2.89, 8.29]
YHQ2018-2	95.41	5.41	10	69.75	8.53	3	4.7%	3.91 [1.70, 6.13]
YHQ2018-3	82.83	5.54	10	69.75	8.53	3	5.7%	1.97 [0.39, 3.54]
YY2019-1	353.66	40.103	12	196.63	36.3	4	5.2%	3.78 [1.89, 5.66]
YY2019-2	315.37	67.52	12	196.63	36.3	4	6.1%	1.81 [0.46, 3.15]
YY2019-3	293.3	67.31	12	196.63	36.3	4	6.2%	1.47 [0.20, 2.75]
ZH2019	425	10.69	10	343.75	5.82	10	3.3%	9.04 [5.79, 12.29]
**Subtotal (95% CI)**			**149**			**74**	**71.5%**	**3.58 [2.75, 4.41]**
Heterogeneity: Tau² = 1.54; Chi² = 37.27, df = 13 (P = 0.0004); I² = 65%								
Test for overall effect: Z = 8.45 (P < 0.00001)								
**Total (95% CI)**			**218**			**107**	**100.0%**	**3.77 [2.98, 4.56]**
Heterogeneity: Tau² = 2.17; Chi² = 68.66, df = 20 (P < 0.00001); I² = 71%								
Test for overall effect: Z = 9.38 (P < 0.00001)								
Test for subgroup differences: Chi² = 0.70, df = 1 (P = 0.40), I² = 0%								

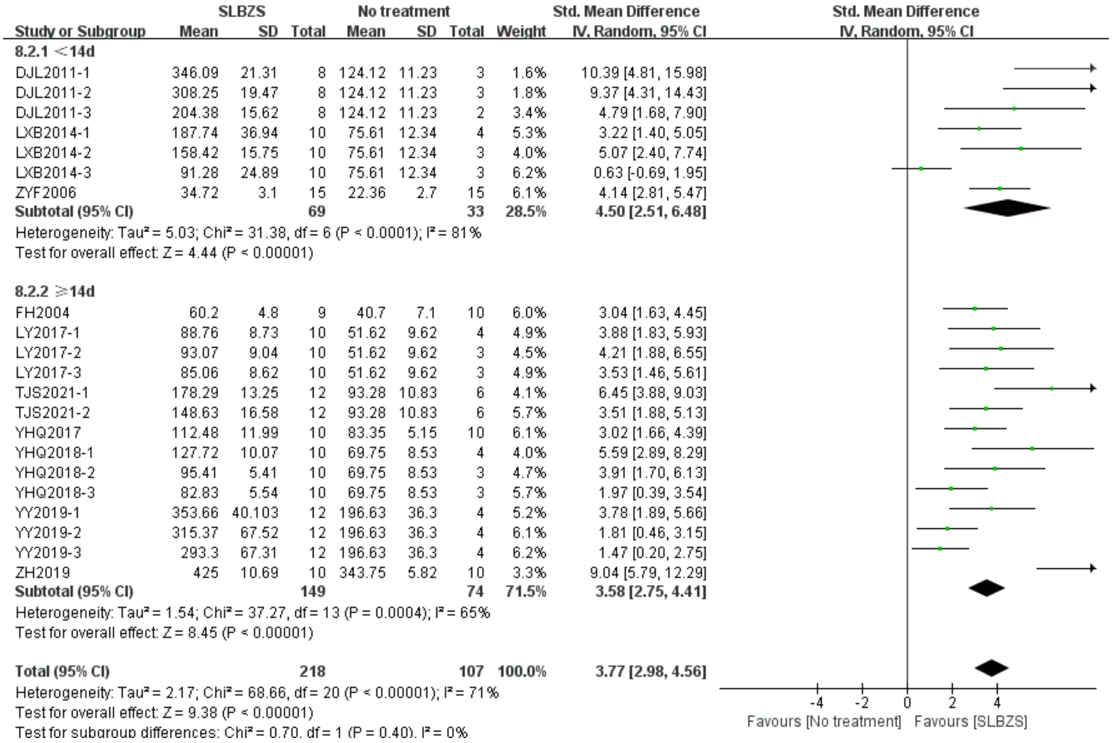

Supplementary figure S15 Subgroup analysis of IL-10 b y different duration

Study or Subgroup	SLBZS Mean	SLBZS SD	SLBZS Total	No treatment Mean	No treatment SD	No treatment Total	Weight	Std. Mean Difference IV, Random, 95% CI
**8.3.1 ≤10g/kg**								
DJL2011-1	346.09	21.31	8	124.12	11.23	3	1.6%	10.39 [4.81, 15.98]
DJL2011-2	308.25	19.47	8	124.12	11.23	3	1.8%	9.37 [4.31, 14.43]
DJL2011-3	204.38	15.62	8	124.12	11.23	2	3.4%	4.79 [1.68, 7.90]
FH2004	60.2	4.8	9	40.7	7.1	10	6.0%	3.04 [1.63, 4.45]
LY2017-1	88.76	8.73	10	51.62	9.62	4	4.9%	3.88 [1.83, 5.93]
LY2017-2	93.07	9.04	10	51.62	9.62	3	4.5%	4.21 [1.88, 6.55]
LY2017-3	85.06	8.62	10	51.62	9.62	3	4.9%	3.53 [1.46, 5.61]
YHQ2018-3	82.83	5.54	10	69.75	8.53	3	5.7%	1.97 [0.39, 3.54]
YY2019-2	315.37	67.52	12	196.63	36.3	4	6.1%	1.81 [0.46, 3.15]
YY2019-3	293.3	67.31	12	196.63	36.3	4	6.2%	1.47 [0.20, 2.75]
**Subtotal (95% CI)**			**97**			**39**	**45.3%**	**3.35 [2.27, 4.44]**
Heterogeneity: Tau² = 1.74; Chi² = 25.50, df = 9 (P = 0.002); I² = 65%								
Test for overall effect: Z = 6.05 (P < 0.00001)								
**8.3.2 10~20g/kg**								
LXB2014-3	91.28	24.89	10	75.61	12.34	3	6.2%	0.63 [-0.69, 1.95]
TJS2021-2	148.63	16.58	12	93.28	10.83	6	5.7%	3.51 [1.88, 5.13]
YHQ2017	112.48	11.99	10	83.35	5.15	10	6.1%	3.02 [1.66, 4.39]
YHQ2018-2	95.41	5.41	10	69.75	8.53	3	4.7%	3.91 [1.70, 6.13]
YY2019-1	353.66	40.103	12	196.63	36.3	4	5.2%	3.78 [1.89, 5.66]
**Subtotal (95% CI)**			**54**			**26**	**27.8%**	**2.86 [1.54, 4.17]**
Heterogeneity: Tau² = 1.52; Chi² = 12.94, df = 4 (P = 0.01); I² = 69%								
Test for overall effect: Z = 4.26 (P < 0.0001)								
**8.3.3 >20g/kg**								
LXB2014-1	187.74	36.94	10	75.61	12.34	4	5.3%	3.22 [1.40, 5.05]
LXB2014-2	158.42	15.75	10	75.61	12.34	3	4.0%	5.07 [2.40, 7.74]
TJS2021-1	178.29	13.25	12	93.28	10.83	6	4.1%	6.45 [3.88, 9.03]
YHQ2018-1	127.72	10.07	10	69.75	8.53	4	4.0%	5.59 [2.89, 8.29]
ZH2019	425	10.69	10	343.75	5.82	10	3.3%	9.04 [5.79, 12.29]
ZYF2006	34.72	3.1	15	22.36	2.7	15	6.1%	4.14 [2.81, 5.47]
**Subtotal (95% CI)**			**67**			**42**	**26.9%**	**5.23 [3.79, 6.68]**
Heterogeneity: Tau² = 1.83; Chi² = 12.32, df = 5 (P = 0.03); I² = 59%								
Test for overall effect: Z = 7.11 (P < 0.00001)								
**Total (95% CI)**			**218**			**107**	**100.0%**	**3.77 [2.98, 4.56]**
Heterogeneity: Tau² = 2.17; Chi² = 68.66, df = 20 (P < 0.00001); I² = 71%								
Test for overall effect: Z = 9.38 (P < 0.00001)								
Test for subgroup differences: Chi² = 6.31, df = 2 (P = 0.04), I² = 68.3%								

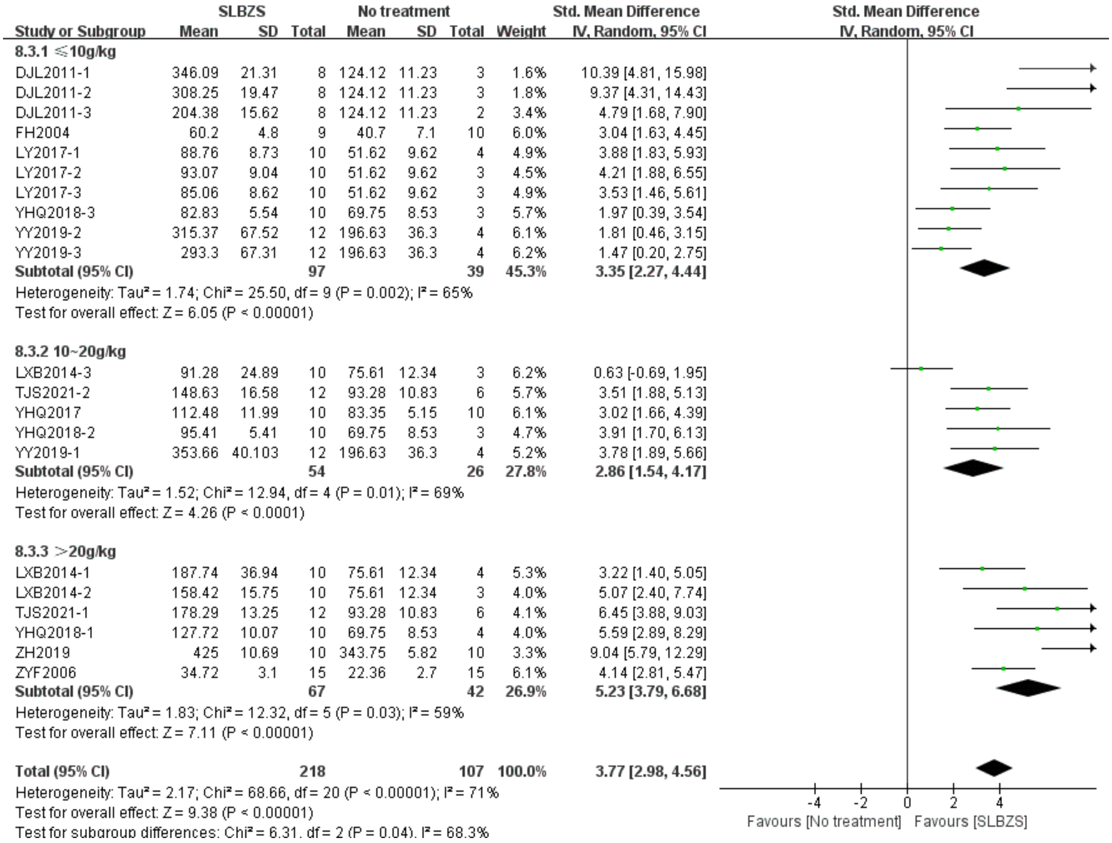

Supplementary figure S16 Subgroup analysis of IL-10 b y different doses

```
Meta-analysis

       |  Pooled      95% CI         Asymptotic      No. of
Method |     Est   Lower   Upper  z_value  p_value   studies
-------+----------------------------------------------------
Fixed  |   3.364   2.972   3.757   16.798    0.000     21
Random |   4.080   3.249   4.912    9.618    0.000

Test for heterogeneity: Q= 80.191 on 20 degrees of freedom (p= 0.000)
Moment-based estimate of between studies variance =  2.583

Trimming estimator: Linear
Meta-analysis type: Random-effects model

iteration |  estimate    Tn    # to trim     diff
----------+--------------------------------------
    1     |    4.080    128        1         231
    2     |    3.921    139        2          22
    3     |    3.773    148        3          18
    4     |    3.533    166        5          36
    5     |    3.249    177        6          22
    6     |    3.149    183        7          12
    7     |    3.070    185        7           4
    8     |    3.070    185        7           0

Filled
Meta-analysis (exponential form)

       |  Pooled      95% CI         Asymptotic      No. of
Method |     Est   Lower   Upper  z_value  p_value   studies
-------+----------------------------------------------------
Fixed  |  19.106  13.167  27.725   15.530    0.000     28
Random |  22.591   9.383  54.391    6.954    0.000

Test for heterogeneity: Q= 132.316 on 27 degrees of freedom (p= 0.000)
Moment-based estimate of between studies variance =  4.019
```

Supplementary figure S17 The trim and fill Analysis results
