# Supplementary material for: Effect of Shenling Baizhu powder on immunity to diarrheal disease: A systematic review and meta-analysis
Source: Front Pharmacol. 2022 Sep 14;13:938932. doi: 10.3389/fphar.2022.938932 (PMC9516002; doi:10.3389/fphar.2022.938932)
Supplement: Supplementary file 3 [file DataSheet3.pdf]

Supplementary Table

Supplementary Table S1： Literature search strategy	
**The database**	**Retrieval type**
**PUBMED**	((((Shenlingbaizhu powder[MeSH Terms]) OR (Shenling baizhu San[MeSH Terms])) OR (slbzs[MeSH Terms])) OR (Shenling baizhu powder[MeSH Terms])) OR (SLBZP[MeSH Terms])
**Cochrane library**	(Shenling baizhu San) OR (slbzs) OR (Shenling baizhu powder) OR (SLBZP) OR (Shenling baizhu)
**Embase**	(Shenling baizhu San) OR (slbzs) OR (Shenling baizhu powder) OR (SLBZP) OR (Shenling baizhu)
**CNKI**	SU=(参苓白术散+参苓白术)*(腹泻+泄泻+肠易激综合征+溃疡性结肠炎+功能性胃肠病+功能性腹泻+炎性肠病+克罗恩病+炎症性肠病+IBD)*(动物+鼠+大鼠+小鼠+犬+狗+猴+兔+蛙)
**WANFANG DATA**	题名或关键词:(参苓白术散 or 参苓白术) and (腹泻 or 泄泻 or 肠易激综合征 or 溃疡性结肠炎 or 功能性胃肠病 or 功能性腹泻 or 炎性肠病 or 克罗恩病 or 炎症性肠病 or IBD) and (动物 or 鼠 or 大鼠 or 小鼠 or 犬 or 狗 or 猴 or 兔 or 蛙)

**CQYIP**	M=(参苓白术散 or 参苓白术) and (腹泻 or 泄泻 or 肠易激综合征 or 溃疡性结肠炎 or 功能性胃肠病 or 功能性腹泻 or 炎性肠病 or 克罗恩病 or 炎症性肠病 or IBD) and (动物 or 鼠 or 大鼠 or 小鼠 or 犬 or 狗 or 猴 or 兔 or 蛙)
**Chinese Medicine Database:**	(("参苓白术 OR 参苓白术散"[主题词]) AND ("腹泻 OR 泄泻 OR 肠易激综合征 OR 溃疡性结肠炎 OR 功能性胃肠病 OR 功能性腹泻 OR 炎性肠病 OR 克罗恩病 OR 炎症性肠病 OR IBD"[主题词]) AND ("动物 OR 鼠 OR 大鼠 OR 小鼠 OR 犬 OR 狗 OR 猴 OR 兔 OR 蛙"[主题词]))

Supplementary Table S2: Preparation composition summary table					
**Study**	**Formulation**	**Source**	**Species, Proportion**	**Quality control reported? (Y/N)**	**Chemical analysis reported? (Y/N)**
**BDY 2017**	SLBZS	The Affiliated Hospital of Gansu University of Chinese Medicine Chinese Medicine Department	Panax ginseng C.A.Mey [Araliaceae; ginseng radix] 10g: Atractylodes macrocephala Koidz [Asteraceae; atractylodis macrocephalae rhizoma]10g: Poria cocos [Polyporaceae;. Poria] 10g:Glycyrrhiza glabra L [Fabaceae; radix et rhizoma glycyrrhizae] 6g: Coicis Semen6g: Nelumbo nucifera Gaertn [Nelumbonaceae; lotus seed]6g: Wurfbainia villosa (Lour.) Skornick. & A.D.Poulsen [Zingiberaceae; amomi fructus] 4g: Lablab purpureus subsp. purpureus [Fabaceae; semen lablab album] 8g: Platycodon grandiflorus (Jacq.) A.DC [Campanulaceae; platycodonis radix] 4g: Dioscorea polystachya Turcz [Dioscoreaceae; dioscoreae rhizoma] 10g: Citrus ×aurantium L [Rutaceae; citri reticulatae pericarpium] 4g, (5:5:5:3:3:3:2:4:2:5:2)	NR	NR
**CH 2007**	SLBZS	Shaanxi Lijun Hengxin Tang Pharmaceutical Co.	Panax ginseng C.A.Mey [Araliaceae; ginseng radix] 8g: Atractylodes macrocephala Koidz [Asteraceae; atractylodis macrocephalae rhizoma]8g: Poria cocos (Polyporaceae;. Poria) 8g:Glycyrrhiza glabra L	Y- Prepared according to Pharmacopoeia of the People's	NR

			[Fabaceae; radix et rhizoma glycyrrhizae]8g : Coix lacryma-jobi var. ma-yuen (Rom.Caill.) Stapf [Poaceae; coicis semen]4g: Nelumbo nucifera Gaertn [Nelumbonaceae; lotus seed]4g: Wurfbainia villosa (Lour.) Skornick. & A.D.Poulsen [Zingiberaceae; amomi fructus] 4g: Lablab purpureus subsp. purpureus [Fabaceae; semen lablab album] 6g: Platycodon grandiflorus (Jacq.) A.DC [Campanulaceae; platycodonis radix] 4g: Dioscorea polystachya Turcz [Dioscoreaceae; dioscoreae rhizoma] 8g, (4:4:4:4:2:2:2:3:2:4)	Republic of China	
**CCH 2004**	SLBZS	Gansu Pharmaceutical Group Gannan Pharmaceutical Factory	Panax ginseng C.A.Mey [Araliaceae; ginseng radix] 8g: Atractylodes macrocephala Koidz [Asteraceae; atractylodis macrocephalae rhizoma]8g: Poria cocos (Polyporaceae;. Poria) 8g:Glycyrrhiza glabra L [Fabaceae; radix et rhizoma glycyrrhizae]8g : Coix lacryma-jobi var. ma-yuen (Rom.Caill.) Stapf [Poaceae; coicis semen]4g: Nelumbo nucifera Gaertn [Nelumbonaceae; lotus seed]4g: Wurfbainia villosa (Lour.) Skornick. & A.D.Poulsen [Zingiberaceae; amomi fructus] 4g: Lablab purpureus subsp. purpureus [Fabaceae; semen lablab album] 6g: Platycodon grandiflorus (Jacq.) A.DC [Campanulaceae; platycodonis radix] 4g: Dioscorea polystachya Turcz [Dioscoreaceae; dioscoreae rhizoma] 8g, (4:4:4:4:2:2:2:3:2:4)	Y- Prepared according to Pharmacopoeia of the People's Republic of China	NR

**DJL 2011**	SLBZS	Wuxi Hospital of Traditional Chinese Medicine	Panax ginseng C.A.Mey [Araliaceae; ginseng radix] 15g: Atractylodes macrocephala Koidz [Asteraceae; atractylodis macrocephalae rhizoma]30g: Poria cocos (Polyporaceae;. Poria) 15g: Coix lacryma-jobi var. ma-yuen (Rom.Caill.) Stapf [Poaceae; coicis semen]30g: Wurfbainia villosa (Lour.) Skornick. & A.D.Poulsen [Zingiberaceae; amomi fructus] 10g: Lablab purpureus subsp. purpureus [Fabaceae; semen lablab album] 10g: Dioscorea polystachya Turcz [Dioscoreaceae; dioscoreae rhizoma] 30g : Citrus × aurantium L [Rutaceae; citri reticulatae pericarpium] 10g (3:6:3:6:2:2:6:2)	NR	NR
**DYW 2007**	SLBZS	Shanxi Huakang Pharmaceutical Co.	Panax ginseng C.A.Mey [Araliaceae; ginseng radix] 8g: Atractylodes macrocephala Koidz [Asteraceae; atractylodis macrocephalae rhizoma]8g: Poria cocos (Polyporaceae;. Poria) 8g:Glycyrrhiza glabra L [Fabaceae; radix et rhizoma glycyrrhizae]8g : Coix lacryma-jobi var. ma-yuen (Rom.Caill.) Stapf [Poaceae; coicis semen]4g: Nelumbo nucifera Gaertn [Nelumbonaceae; lotus seed]4g: Wurfbainia villosa (Lour.) Skornick. & A.D.Poulsen [Zingiberaceae; amomi fructus] 4g: Lablab purpureus subsp. purpureus [Fabaceae; semen lablab album] 6g: Platycodon grandiflorus (Jacq.) A.DC [Campanulaceae; platycodonis radix] 4g: Dioscorea polystachya Turcz [Dioscoreaceae; dioscoreae	Y- Prepared according to Pharmacopoeia of the People's Republic of China	NR

			rhizoma] 8g , (4:4:4:4:2:2:2:3:2:4)		
**FH 2004**	SLBZS	NR	Panax ginseng C.A.Mey [Araliaceae; ginseng radix] 20g: Atractylodes macrocephala Koidz [Asteraceae; atractylodis macrocephalae rhizoma]20g: Poria cocos [Polyporaceae;. Poria] 20g:Glycyrrhiza glabra L [Fabaceae; radix et rhizoma glycyrrhizae] 20g : Coix lacryma-jobi var. ma-yuen (Rom.Caill.) Stapf [Poaceae; coicis semen]10g: Nelumbo nucifera Gaertn [Nelumbonaceae; lotus seed]10g: Wurfbainia villosa (Lour.) Skornick. & A.D.Poulsen [Zingiberaceae; amomi fructus] 10g: Lablab purpureus subsp. purpureus [Fabaceae; semen lablab album] 15g: Platycodon grandiflorus (Jacq.) A.DC [Campanulaceae; platycodonis radix] 10g: Dioscorea polystachya Turcz [Dioscoreaceae; dioscoreae rhizoma] 40g, (4:4:4:4:2:2:2:3:2:4)	NR	NR
**HHR 2008**	SLBZS	NR	Panax ginseng C.A.Mey [Araliaceae; ginseng radix] 8g: Atractylodes macrocephala Koidz [Asteraceae; atractylodis macrocephalae rhizoma]8g: Poria cocos [Polyporaceae;. Poria] 8g:Glycyrrhiza glabra L [Fabaceae; radix et rhizoma glycyrrhizae]8g : Coix lacryma-jobi var. ma-yuen (Rom.Caill.) Stapf [Poaceae; coicis semen]4g: Nelumbo nucifera Gaertn [Nelumbonaceae; lotus seed]4g: Wurfbainia villosa (Lour.) Skornick. & A.D.Poulsen [Zingiberaceae; amomi fructus] 4g: Lablab purpureus subsp.	NR	NR

			purpureus [Fabaceae; semen lablab album] 6g: Platycodon grandiflorus (Jacq.) A.DC [Campanulaceae; platycodonis radix] 4g: Dioscorea polystachya Turcz [Dioscoreaceae; dioscoreae rhizoma] 8g, (4:4:4:4:2:2:2:3:2:4)		
**JYX 2016**	SLBZS	The Affiliated Hospital of Gansu University of Chinese Medicine Chinese Medicine Department	Panax ginseng C.A.Mey [Araliaceae; ginseng radix] 10g: Atractylodes macrocephala Koidz [Asteraceae; atractylodis macrocephalae rhizoma]10g: Poria cocos [Polyporaceae;. Poria] 10g:Glycyrrhiza glabra L [Fabaceae; radix et rhizoma glycyrrhizae] 6g: Coix lacryma-jobi var. ma-yuen (Rom.Caill.) Stapf [Poaceae; coicis semen]6g: Nelumbo nucifera Gaertn [Nelumbonaceae; lotus seed]6g: Wurfbainia villosa (Lour.) Skornick. & A.D.Poulsen [Zingiberaceae; amomi fructus] 4g: Lablab purpureus subsp. purpureus [Fabaceae; semen lablab album] 8g: Platycodon grandiflorus (Jacq.) A.DC [Campanulaceae; platycodonis radix] 4g: Dioscorea polystachya Turcz [Dioscoreaceae; dioscoreae rhizoma] 10g: Citrus ×aurantium L [Rutaceae; citri reticulatae pericarpium] 4g,(5:5:5:3:3:3:2:4:2:5:2)	NR	NR
**JMR 2010**	SLBZS	Shaanxi College of Traditional Chinese Medicine - Golden Autumn Outpatient Chinese Medicine	Panax ginseng C.A.Mey [Araliaceae; ginseng radix] 8g: Atractylodes macrocephala Koidz [Asteraceae; atractylodis macrocephalae rhizoma]8g: Poria cocos [Polyporaceae;. Poria] 8g:Glycyrrhiza glabra L [Fabaceae; radix et rhizoma glycyrrhizae]8g : Coix	Y- Prepared according to Pharmacopoeia of the People's Republic of China	NR

		Clinic	lacryma-jobi var. ma-yuen (Rom.Caill.) Stapf [Poaceae; coicis semen]4g: Nelumbo nucifera Gaertn [Nelumbonaceae; lotus seed]4g: Wurfbainia villosa (Lour.) Skornick. & A.D.Poulsen [Zingiberaceae; amomi fructus] 4g: Lablab purpureus subsp. purpureus [Fabaceae; semen lablab album] 6g: Platycodon grandiflorus (Jacq.) A.DC [Campanulaceae; platycodonis radix] 4g: Dioscorea polystachya Turcz [Dioscoreaceae; dioscoreae rhizoma] 8g (4:4:4:4:2:2:2:3:2:4)		
**LJ 2016**	SLBZS	Kangmei Pharmaceutical Co.	Panax ginseng C.A.Mey [Araliaceae; ginseng radix] 15g: Atractylodes macrocephala Koidz [Asteraceae; atractylodis macrocephalae rhizoma]15g: Poria cocos [Polyporaceae;. Poria] 15g:Glycyrrhiza glabra L [Fabaceae; radix et rhizoma glycyrrhizae] 10g: Coix lacryma-jobi var. ma-yuen (Rom.Caill.) Stapf [Poaceae; coicis semen]10g: Nelumbo nucifera Gaertn [Nelumbonaceae; lotus seed]10g: Wurfbainia villosa (Lour.) Skornick. & A.D.Poulsen [Zingiberaceae; amomi fructus] 6g: Lablab purpureus subsp. purpureus [Fabaceae; semen lablab album] 12g: Platycodon grandiflorus (Jacq.) A.DC [Campanulaceae; platycodonis radix] 6g: Dioscorea polystachya Turcz [Dioscoreaceae; dioscoreae rhizoma] 15g , (15:15:15:10:10:10:6:12:6:15)	NR	NR
**LWX**	SLBZS	The First affiliated	Panax ginseng C.A.Mey [Araliaceae; ginseng radix]	Y- Prepared	NR

**2015**		Hospital of Hunan Chinese Medicine College	10g: Atractylodes macrocephala Koidz [Asteraceae; atractylodis macrocephalae rhizoma]10g: Poria cocos [Polyporaceae;. Poria] 10g:Glycyrrhiza glabra L [Fabaceae; radix et rhizoma glycyrrhizae] 6g: Coix lacryma-jobi var. ma-yuen (Rom.Caill.) Stapf [Poaceae; coicis semen]6g: Nelumbo nucifera Gaertn [Nelumbonaceae; lotus seed]6g: Wurfbainia villosa (Lour.) Skornick. & A.D.Poulsen [Zingiberaceae; amomi fructus] 4g: Lablab purpureus subsp. purpureus [Fabaceae; semen lablab album] 8g: Platycodon grandiflorus (Jacq.) A.DC [Campanulaceae; platycodonis radix] 4g: Dioscorea polystachya Turcz [Dioscoreaceae; dioscoreae rhizoma] 10g, (5:5:5:3:3:3:2:4:2:5)	according to Pharmacopoeia of the People's Republic of China	
**LXB 2014**	SLBZS	Shanxi Huakang Pharmaceutical Co.	Panax ginseng C.A.Mey [Araliaceae; ginseng radix] 8g: Atractylodes macrocephala Koidz [Asteraceae; atractylodis macrocephalae rhizoma]8g: Poria cocos [Polyporaceae;. Poria] 8g:Glycyrrhiza glabra L [Fabaceae; radix et rhizoma glycyrrhizae]8g : Coix lacryma-jobi var. ma-yuen (Rom.Caill.) Stapf [Poaceae; coicis semen]4g: Nelumbo nucifera Gaertn [Nelumbonaceae; lotus seed]4g: Wurfbainia villosa (Lour.) Skornick. & A.D.Poulsen [Zingiberaceae; amomi fructus] 4g: Lablab purpureus subsp. purpureus [Fabaceae; semen lablab album] 6g: Platycodon grandiflorus (Jacq.) A.DC	Y- Prepared according to Pharmacopoeia of the People's Republic of China	NR

			[Campanulaceae; platycodonis radix] 4g: Dioscorea polystachya Turcz [Dioscoreaceae; dioscoreae rhizoma] 8g, (4:4:4:4:2:2:2:3:2:4)		
**LY 2017**	SLBZS	Shaanxi University of Traditional Chinese Medicine Hospital Chinese Medicine Department	Panax ginseng C.A.Mey [Araliaceae; ginseng radix] 8g: Atractylodes macrocephala Koidz [Asteraceae; atractylodis macrocephalae rhizoma]8g: Poria cocos [Polyporaceae;. Poria] 8g:Glycyrrhiza glabra L [Fabaceae; radix et rhizoma glycyrrhizae]8g : Coix lacryma-jobi var. ma-yuen (Rom.Caill.) Stapf [Poaceae; coicis semen]4g: Nelumbo nucifera Gaertn [Nelumbonaceae; lotus seed]4g: Wurfbainia villosa (Lour.) Skornick. & A.D.Poulsen [Zingiberaceae; amomi fructus] 4g: Lablab purpureus subsp. purpureus [Fabaceae; semen lablab album] 6g: Platycodon grandiflorus (Jacq.) A.DC [Campanulaceae; platycodonis radix] 4g: Dioscorea polystachya Turcz [Dioscoreaceae; dioscoreae rhizoma] 8g, (4:4:4:4:2:2:2:3:2:4)	Y- Prepared according to Pharmacopoeia of the People's Republic of China	NR
**LZH 2015**	SLBZS	Anhui University of Chinese Medicine Chinese Medicine Preparation Room	Panax ginseng C.A.Mey [Araliaceae; ginseng radix] 10g: Atractylodes macrocephala Koidz [Asteraceae; atractylodis macrocephalae rhizoma]10g: Poria cocos [Polyporaceae;. Poria] 10g:Glycyrrhiza glabra L [Fabaceae; radix et rhizoma glycyrrhizae] 6g: Coix lacryma-jobi var. ma-yuen (Rom.Caill.) Stapf [Poaceae; coicis semen]6g: Nelumbo nucifera Gaertn [Nelumbonaceae; lotus seed]6g: Wurfbainia villosa	NR	NR

			(Lour.) Skornick. & A.D.Poulsen [Zingiberaceae; amomi fructus] 4g: Lablab purpureus subsp. purpureus [Fabaceae; semen lablab album] 8g: Platycodon grandiflorus (Jacq.) A.DC [Campanulaceae; platycodonis radix] 4g: Dioscorea polystachya Turcz [Dioscoreaceae; dioscoreae rhizoma] 10g, (5:5:5:3:3:3:2:4:2:5)		
**LZH 2020**	SLBZS	Anhui University of Chinese Medicine Chinese Medicine Preparation Room	Panax ginseng C.A.Mey [Araliaceae; ginseng radix] 10g: Atractylodes macrocephala Koidz [Asteraceae; atractylodis macrocephalae rhizoma]10g: Poria cocos [Polyporaceae;. Poria] 10g:Glycyrrhiza glabra L [Fabaceae; radix et rhizoma glycyrrhizae] 6g: Coix lacryma-jobi var. ma-yuen (Rom.Caill.) Stapf [Poaceae; coicis semen]6g: Nelumbo nucifera Gaertn [Nelumbonaceae; lotus seed]6g: Wurfbainia villosa (Lour.) Skornick. & A.D.Poulsen [Zingiberaceae; amomi fructus] 4g: Lablab purpureus subsp. purpureus [Fabaceae; semen lablab album] 8g: Platycodon grandiflorus (Jacq.) A.DC [Campanulaceae; platycodonis radix] 4g: Dioscorea polystachya Turcz [Dioscoreaceae; dioscoreae rhizoma] 10g, (5:5:5:3:3:3:2:4:2:5)	NR	NR
**LHW 2010**	SLBZS	Shaanxi College of Traditional Chinese Medicine - Golden Autumn Outpatient	Panax ginseng C.A.Mey [Araliaceae; ginseng radix] 8g: Atractylodes macrocephala Koidz [Asteraceae; atractylodis macrocephalae rhizoma]8g: Poria cocos [Polyporaceae;. Poria] 8g:Glycyrrhiza glabra L	Y- Prepared according to Pharmacopoeia of the People's	NR

		Chinese Medicine Clinic	[Fabaceae; radix et rhizoma glycyrrhizae]8g : Coix lacryma-jobi var. ma-yuen (Rom.Caill.) Stapf [Poaceae; coicis semen]4g: Nelumbo nucifera Gaertn [Nelumbonaceae; lotus seed]4g: Wurfbainia villosa (Lour.) Skornick. & A.D.Poulsen [Zingiberaceae; amomi fructus] 4g: Lablab purpureus subsp. purpureus [Fabaceae; semen lablab album] 6g: Platycodon grandiflorus (Jacq.) A.DC [Campanulaceae; platycodonis radix] 4g: Dioscorea polystachya Turcz [Dioscoreaceae; dioscoreae rhizoma] 8g, (4:4:4:4:2:2:2:3:2:4)	Republic of China	
**SY 2018**	SLBZS	Yingpan Pharmacy	Panax ginseng C.A.Mey [Araliaceae; ginseng radix] 8g: Atractylodes macrocephala Koidz [Asteraceae; atractylodis macrocephalae rhizoma]8g: Poria cocos [Polyporaceae;. Poria] 8g:Glycyrrhiza glabra L [Fabaceae; radix et rhizoma glycyrrhizae]8g : Coix lacryma-jobi var. ma-yuen (Rom.Caill.) Stapf [Poaceae; coicis semen]4g: Nelumbo nucifera Gaertn [Nelumbonaceae; lotus seed]4g: Wurfbainia villosa (Lour.) Skornick. & A.D.Poulsen [Zingiberaceae; amomi fructus] 4g: Lablab purpureus subsp. purpureus [Fabaceae; semen lablab album] 6g: Platycodon grandiflorus (Jacq.) A.DC [Campanulaceae; platycodonis radix] 4g: Dioscorea polystachya Turcz [Dioscoreaceae; dioscoreae rhizoma] 8g, (4:4:4:4:2:2:2:3:2:4)	NR	NR

**SSZ 2020**	SLBZS	Beijing Tongrentang Company Limited	Panax ginseng C.A.Mey [Araliaceae; ginseng radix] 8g: Atractylodes macrocephala Koidz [Asteraceae; atractylodis macrocephalae rhizoma]8g: Poria cocos [Polyporaceae;. Poria] 8g:Glycyrrhiza glabra L [Fabaceae; radix et rhizoma glycyrrhizae]8g : Coix lacryma-jobi var. ma-yuen (Rom.Caill.) Stapf [Poaceae; coicis semen]4g: Nelumbo nucifera Gaertn [Nelumbonaceae; lotus seed]4g: Wurfbainia villosa (Lour.) Skornick. & A.D.Poulsen [Zingiberaceae; amomi fructus] 4g: Lablab purpureus subsp. purpureus [Fabaceae; semen lablab album] 6g: Platycodon grandiflorus (Jacq.) A.DC [Campanulaceae; platycodonis radix] 4g: Dioscorea polystachya Turcz [Dioscoreaceae; dioscoreae rhizoma] 8g, (4:4:4:4:2:2:2:3:2:4)	Y- Prepared according to Pharmacopoeia of the People's Republic of China	NR
**TJS 2021**	SLBZS	Pharmacy of the Affiliated Hospital of Heilongjiang University of Chinese Medicine	Panax ginseng C.A.Mey [Araliaceae; ginseng radix] 15g: Atractylodes macrocephala Koidz [Asteraceae; atractylodis macrocephalae rhizoma]15g: Poria cocos [Polyporaceae;. Poria] 15g:Glycyrrhiza glabra L [Fabaceae; radix et rhizoma glycyrrhizae] 9g: Coix lacryma-jobi var. ma-yuen (Rom.Caill.) Stapf [Poaceae; coicis semen]9g: Nelumbo nucifera Gaertn [Nelumbonaceae; lotus seed]9g: Wurfbainia villosa (Lour.) Skornick. & A.D.Poulsen [Zingiberaceae; amomi fructus] 6g: Lablab purpureus subsp. purpureus [Fabaceae; semen lablab album] 12g:	NR	NR

			Platycodon grandiflorus (Jacq.) A.DC [Campanulaceae; platycodonis radix] 6g: Dioscorea polystachya Turcz [Dioscoreaceae; dioscoreae rhizoma] 15g: Citrus ×aurantium L [Rutaceae; citri reticulatae pericarpium] 6g, (5:5:5:3:3:3:2:4:2:5:2)		
**YY 2019**	SLBZS	Yunnan Baiyao Group Co.	Panax ginseng C.A.Mey [Araliaceae; ginseng radix] 8g: Atractylodes macrocephala Koidz [Asteraceae; atractylodis macrocephalae rhizoma]8g: Poria cocos [Polyporaceae;. Poria] 8g:Glycyrrhiza glabra L [Fabaceae; radix et rhizoma glycyrrhizae]8g : Coix lacryma-jobi var. ma-yuen (Rom.Caill.) Stapf [Poaceae; coicis semen]4g: Nelumbo nucifera Gaertn [Nelumbonaceae; lotus seed]4g: Wurfbainia villosa (Lour.) Skornick. & A.D.Poulsen [Zingiberaceae; amomi fructus] 4g: Lablab purpureus subsp. purpureus [Fabaceae; semen lablab album] 6g: Platycodon grandiflorus (Jacq.) A.DC [Campanulaceae; platycodonis radix] 4g: Dioscorea polystachya Turcz [Dioscoreaceae; dioscoreae rhizoma] 8g, (4:4:4:4:2:2:2:3:2:4)	Y- Prepared according to Pharmacopoeia of the People's Republic of China	NR
**YHQ 2017**	SLBZS	Affiliated Hospital of Gansu College of Traditional Chinese Medicine	Panax ginseng C.A.Mey [Araliaceae; ginseng radix] 10g: Atractylodes macrocephala Koidz [Asteraceae; atractylodis macrocephalae rhizoma]10g: Poria cocos [Polyporaceae;. Poria] 10g:Glycyrrhiza glabra L [Fabaceae; radix et rhizoma glycyrrhizae] 6g: Coix lacryma-jobi var. ma-yuen (Rom.Caill.) Stapf	NR	NR

			[Poaceae; coicis semen]6g: Nelumbo nucifera Gaertn [Nelumbonaceae; lotus seed]6g: Wurfbainia villosa (Lour.) Skornick. & A.D.Poulsen [Zingiberaceae; amomi fructus] 4g: Lablab purpureus subsp. purpureus [Fabaceae; semen lablab album] 8g: Platycodon grandiflorus (Jacq.) A.DC [Campanulaceae; platycodonis radix] 4g: Dioscorea polystachya Turcz [Dioscoreaceae; dioscoreae rhizoma] 10g: Citrus ×aurantium L [Rutaceae; citri reticulatae pericarpium] 4g, (5:5:5:3:3:3:2:4:2:5:2)		
**YHQ 2018**	SLBZS	Affiliated Hospital of Gansu College of Traditional Chinese Medicine	Panax ginseng C.A.Mey [Araliaceae; ginseng radix] 10g: Atractylodes macrocephala Koidz [Asteraceae; atractylodis macrocephalae rhizoma]10g: Poria cocos [Polyporaceae;. Poria] 10g:Glycyrrhiza glabra L [Fabaceae; radix et rhizoma glycyrrhizae] 6g: Coix lacryma-jobi var. ma-yuen (Rom.Caill.) Stapf [Poaceae; coicis semen]6g: Nelumbo nucifera Gaertn [Nelumbonaceae; lotus seed]6g: Wurfbainia villosa (Lour.) Skornick. & A.D.Poulsen [Zingiberaceae; amomi fructus] 4g: Lablab purpureus subsp. purpureus [Fabaceae; semen lablab album] 8g: Platycodon grandiflorus (Jacq.) A.DC [Campanulaceae; platycodonis radix] 4g: Dioscorea polystachya Turcz [Dioscoreaceae; dioscoreae rhizoma] 10g: Citrus ×aurantium L [Rutaceae; citri reticulatae pericarpium] 4g, (5:5:5:3:3:3:2:4:2:5:2)	NR	NR

**ZJJ 2019**	SLBZS	The First Affiliated Hospital of Guangzhou University of Chinese Medicine	Panax ginseng C.A.Mey [Araliaceae; ginseng radix] 15g: Atractylodes macrocephala Koidz [Asteraceae; atractylodis macrocephalae rhizoma]15g: Poria cocos [Polyporaceae;. Poria] 15g:Glycyrrhiza glabra L [Fabaceae; radix et rhizoma glycyrrhizae] 9g: Nelumbo nucifera Gaertn [Nelumbonaceae; lotus seed]9g: Dioscorea polystachya Turcz [Dioscoreaceae; dioscoreae rhizoma] 9g: Platycodon grandiflorus (Jacq.) A.DC [Campanulaceae; platycodonis radix] 15g: Wurfbainia villosa (Lour.) Skornick. & A.D.Poulsen [Zingiberaceae; amomi fructus] 9g, (5:5:5:3:3:5:3:2)	Y- Prepared according to Pharmacopoeia of the People's Republic of China	NR
**ZJL 2010**	SLBZS	Shaanxi College of Traditional Chinese Medicine - Golden Autumn Outpatient Chinese Medicine Clinic	Panax ginseng C.A.Mey [Araliaceae; ginseng radix] 8g: Atractylodes macrocephala Koidz [Asteraceae; atractylodis macrocephalae rhizoma]8g: Poria cocos [Polyporaceae;. Poria] 8g:Glycyrrhiza glabra L [Fabaceae; radix et rhizoma glycyrrhizae]8g : Coix lacryma-jobi var. ma-yuen (Rom.Caill.) Stapf [Poaceae; coicis semen]4g: Nelumbo nucifera Gaertn [Nelumbonaceae; lotus seed]4g: Wurfbainia villosa (Lour.) Skornick. & A.D.Poulsen [Zingiberaceae; amomi fructus] 4g: Lablab purpureus subsp. purpureus [Fabaceae; semen lablab album] 6g: Platycodon grandiflorus (Jacq.) A.DC [Campanulaceae; platycodonis radix] 4g: Dioscorea polystachya Turcz [Dioscoreaceae; dioscoreae	Y- Prepared according to Pharmacopoeia of the People's Republic of China	NR

			rhizoma] 8g, (4:4:4:4:2:2:2:3:2:4)		
**ZYF 2006**	SLBZS	NR	Panax ginseng C.A.Mey [Araliaceae; ginseng radix] 20g: Atractylodes macrocephala Koidz [Asteraceae; atractylodis macrocephalae rhizoma]20g: Poria cocos [Polyporaceae;. Poria] 20g:Glycyrrhiza glabra L [Fabaceae; radix et rhizoma glycyrrhizae] 20g : Coix lacryma-jobi var. ma-yuen (Rom.Caill.) Stapf [Poaceae; coicis semen]10g: Nelumbo nucifera Gaertn [Nelumbonaceae; lotus seed]10g: Wurfbainia villosa (Lour.) Skornick. & A.D.Poulsen [Zingiberaceae; amomi fructus] 10g: Lablab purpureus subsp. purpureus [Fabaceae; semen lablab album] 15g: Platycodon grandiflorus (Jacq.) A.DC [Campanulaceae; platycodonis radix] 10g: Dioscorea polystachya Turcz [Dioscoreaceae; dioscoreae rhizoma] 20g, (4:4:4:4:2:2:2:3:2:4)	Y- Y- Prepared according to formulary of peaceful benevolent dispensary	NR
**ZH 2019**	SLBZS	The First Affiliated Hospital of Anhui University of Chinese Medicine, Chinese Medicine Preparation Room	Panax ginseng C.A.Mey [Araliaceae; ginseng radix] 8g: Atractylodes macrocephala Koidz [Asteraceae; atractylodis macrocephalae rhizoma]8g: Poria cocos [Polyporaceae;. Poria] 8g:Glycyrrhiza glabra L [Fabaceae; radix et rhizoma glycyrrhizae]8g : Coix lacryma-jobi var. ma-yuen (Rom.Caill.) Stapf [Poaceae; coicis semen]4g: Nelumbo nucifera Gaertn [Nelumbonaceae; lotus seed]4g: Wurfbainia villosa (Lour.) Skornick. & A.D.Poulsen [Zingiberaceae; amomi fructus] 4g: Lablab purpureus subsp.	NR	NR

purpureus [Fabaceae; semen lablab album] 6g: Platycodon grandiflorus (Jacq.) A.DC [Campanulaceae; platycodonis radix] 4g: Dioscorea polystachya Turcz [Dioscoreaceae; dioscoreae rhizoma] 8g, (4:4:4:4:2:2:2:3:2:4)

Note: Shenling Baizhu Powder, SLBZS; NR, No report

Supplementary Table S3: GRADE evidence profile and summary of findings table for rodent studies.

Outcome	No of Studies	Risk of Bias	Inconsistency	Indirectness	Imprecision	Publication Bias	Sample size		Effect Size [95% CI]	Certainty
							SLBZS	No treatment		
Body weight	9	Serious[1]	Serious[2]	Serious[4]	Not Serious	Serious[6]	93	93	SMD 2.28 (1.4 to 3.16)	⊕Very Low
Diarrhea score	4	Serious[1]	Not Serious	Serious[4]	Serious[5]	Undetected	39	23	SMD 1.4 (2.03 to 0.78)	⊕Very Low
Spleen weight	5	Serious[1]	Not Serious	Serious[4]	Serious[5]	Serious[6]	53	53	SMD 1.42 (0.98 to 1.87)	⊕Very Low
Thymus weight	5	Serious[1]	Not Serious	Serious[4]	Serious[5]	Undetected	53	53	SMD 1.11 (0.69 to 1.53)	⊕Very Low
Macrophage phagocytosis	3	Serious[1]	Not Serious	Serious[4]	Serious[5]	Undetected	40	40	SMD 1.07 (0.59 to 1.54)	⊕Very Low
RBC-IC-RR	3	Serious[1]	Not Serious	Serious[4]	Serious[5]	Undetected	35	35	SMD 1.4 (1.94 to 0.87)	⊕Very Low
RBC-C3b-R R	3	Serious[1]	Not Serious	Serious[4]	Serious[5]	Undetected	35	35	SMD 1.16 (0.65 to 1.67)	⊕Very Low
sIgA	3	Serious[1]	Not Serious	Serious[4]	Serious[5]	Serious[6]	28	28	SMD 0.36 (0.2 to 0.92)	⊕Very Low
IL-8	4	Serious[1]	Not Serious	Serious[4]	Serious[5]	Serious[6]	48	24	SMD 2.8 (3.54 to 2.07)	⊕Very Low
IL-2	3	Serious[1]	Not Serious	Serious[4]	Serious[5]	Undetected	28	28	SMD 1.52 (0.89 to 2.14)	⊕Very Low

Note: [1] The includedstudies were significantly biased with respect to randomization methods, allocation concealment, and blind method.[2] Study

heterogeneity was large($50\% < I2 < 75\%$).[3] Inclusion of studies was highly heterogeneous ($I^2 > 75\%$).[4] All outcomes due to the indirectness inherent in non-primate animal models.[5] The sample size was small.[6]The results of the funnel plot or Egger's test indicated the presence of publication bias.

Supplementary Table S4: The composition of Shenling Baizhu Powder

Name	Composition	Function
Panax ginseng C.A.Mey [Araliaceae; ginseng radix] (Park et al., 2018)	ginsenosides Re, Rg2, Rh1, phytosterol, stigmasterol, and β-sitosterol, β-enamine and β-Se, kaempferol, alkaloids, elemicin,dauricine, etc.	Metabolic processes, signal transduction, nitrogen compound metabolic processes, blood circulation, immune system processes, cell-cell signaling, biosynthesis processes and nervous system processes are related, etc.
Poria cocos (Polyporaceae;. Poria) (Zou et al., 2021)	poricoic acid， linoleic acid, (R)-3-hydroxybutyric acid, D-ribose, thiamine monophosphate, indolelactic acid and plamitic acid, etc.	Regulation of intestinal flora, immune regulation, etc.
Atractylodes macrocephala Koidz [Asteraceae; atractylodis macrocephalae rhizoma] (Ruqiao et al., 2020)	volatile oil, lactones, polysaccharides, amino acids, vitamins and resins, etc.	immune and anti-inflammatory activity, antitumor activities, neuroprotective effect, anti-hepatotoxicity, etc.
Lablab purpureus subsp. purpureus [Fabaceae;	α -linalool, isonolenone, Methyl palmitate, methyl linoleate, 2-methyleicosane	Antibacterial and antiviral, improve immunity, detoxify, reduce blood sugar and cholesterol, antioxidant activity, etc.

semen lablab album] (Zhang et al., 2020)		
Nelumbo nucifera Gaertn [Nelumbonaceae; lotus seed] (Shi et al., 2018)	Alkaloids, flavonoids and polysaccharides,etc.	Anti-inflammatory, antioxidant, hypoglycemic, anti-tumor, etc.
Glycyrrhiza glabra L [Fabaceae; radix et rhizoma glycyrrhizae] (Jiang et al., 2020; Cai et al., 2021)	Liquiritin, isoliquiritin apioside, isoliquiritin, liquiritigenin, glycyrrhizic acid, etc.	liver protection, digestive system and nervous system protection, anticancer, anti-inflammatory, anti-allergy, anti-AIDS, etc.
Dioscorea polystachya Turcz [Dioscoreaceae; dioscoreae rhizoma] (Pan et al.)	Dioscorea saponins, furanostanol saponins, 3,5-Dimethoxyquercetin, Catechin, Quercetin, Kaempferol, 2,4,3',5'-Tetrahydroxybibenzyl, myricetin, diospersimilosides A,B, diosbiphenanthrene, linoleic acid, linolenic acid, etc.	Immune regulation, anti-tumor, anti-oxidation, anti-aging, hypoglycemic, hypolipidemic, etc.
Wurfbainia	volatile oils, saponins, flavonoids, organic acids,	Gastrointestinal protection, anti-inflammatory activity,

villosa (Lour.) Skornick. & A.D.Poulsen [Zingiberaceae; amomi fructus] (Suo et al., 2018)	inorganic ingredients, and polysaccharides	analgesic activity, antidiarrheal activity, antibacterial activity, etc.
Coix lacryma-jobi var. ma-yuen (Rom.Caill.) Stapf [Poaceae; coicis semen] (Zhu et al., 2020)	Glycerol monoester, diglycerol, triglyceride, stigmasterol, etc.	Anti-inflammatory, antioxidant, analgesic and sedative effects, etc.
Platycodon grandiflorus (Jacq.) A.DC [Campanulaceae; platycodonis radix] (Chen et al., 2020)	Apigenin, luteolin, tangerine, quercetin - 7-O-glucoside, quercetin - 7-O-rutin, etc.	Anti - tumor, liver protection, anti - inflammation, anti - oxidation, etc.
SLBZS (Lu et al.)	ginsenosides, quercetin, lignan, β-sitosterol, β-carotene, kaempferol, origanoside D, rutin, atractylenolide III, poric acid, psoralen, iso-psoralen, iso-prenyl, and lobsteryl acetate, etc.	Regulate the immune system, improve lung function, improve gastrointestinal function, antioxidant, anti-tumor, anti-inflammatory, etc

References：

Cai, S.H., Zhao, H.C., Jia, M., Zhao, X.L., Chi, Y.M., Zhang, W., et al. (2021). [Quality evaluation of fried Glycyrrhizae Radix et Rhizoma pieces by HPLC fingerprint and multicomponent quantitative analysis]. *Zhongguo Zhong Yao Za Zhi* 46(1), 118-124. doi: 10.19540/j.cnki.cjcmm.20201022.306.

Chen, D.D., Hong, T., Wang, D., and Yang, Y.S. (2020). A Review of the Research on the Chemical Constituents and Pharmacological Effects of Platycodon Grandiflorum. *Drug Evaluation* 17(15), 9-11.

Jiang, M., Zhao, S., Yang, S., Lin, X., He, X., Wei, X., et al. (2020). An "essential herbal medicine"-licorice: A review of phytochemicals and its effects in combination preparations. *J Ethnopharmacol* 249, 112439. doi: 10.1016/j.jep.2019.112439.

Lu, G.Y., Xin, X.Y., Wang, J.J., Wang, Y., Ma, K., and Wang, S.J. Research progress of Shenling Baizhu San and predictive analysis on guality markers. *China Journal of Chinese Materia Medica*, 1-13. doi: 10.19540/j.cnki.cjcmm.20220421.201.

Pan, J.Z., Meng, Q.L., Cui, W.Y., and Zhu, S.J. Advances in studies on functional componets and pharmacological effects of Dioscorea opposita Thunb. *Science and Technology of Food Industry*, 1-15. doi: 10.13386/j.issn1002-0306.2022010212.

Park, S.Y., Park, J.H., Kim, H.S., Lee, C.Y., Lee, H.J., Kang, K.S., et al. (2018). Systems-level mechanisms of action of Panax ginseng: a network pharmacological approach. *J Ginseng Res* 42(1), 98-106. doi: 10.1016/j.jgr.2017.09.001.

Ruqiao, L., Yueli, C., Xuelan, Z., Huifen, L., Xin, Z., Danjie, Z., et al. (2020). Rhizoma Atractylodis macrocephalae: a review of photochemistry, pharmacokinetics and pharmacology. *Pharmazie* 75(2), 42-55. doi: 10.1691/ph.2020.9738.

Shi, J.Y., Wang, M., and Wu, T. (2018). Research Progress of Chemical Constituents and Biological Activities of Nelumbinis Plumula *Guiding Journal of Traditional Chinese Medicine and PharmacY* 24(21), 105-108. doi: 10.13862/j.cnki.cn43-1446/r.2018.21.035.

Suo, S., Lai, Y., Li, M., Song, Q., Cai, J., Zhao, J., et al. (2018). Phytochemicals, pharmacology, clinical application, patents, and products of Amomi fructus. *Food ChemToxicol* 119, 31-36. doi: 10.1016/j.fct.2018.05.051.

Zhang, X.X., Yang, X.J., Peng, X.Y., Wang, Y.C., Zhou, L., Liu, X.C., et al. (2020). Analysis of Volatile Components in Different Parts of Dolichos Lablab by GC-MS. *China Pharmaceuticals* 29(21), 12-14.

Zhu, R., Xu, X., Shan, Q., Wang, K., Cao, G., and Wu, X. (2020). Determination of Differentiating Markers in Coicis Semen From Multi-Sources Based on Structural Similarity Classification Coupled With UPCC-Xevo G2-XS QTOF. *Front Pharmacol* 11, 549181. doi: 10.3389/fphar.2020.549181.

Zou, Y.T., Zhou, J., Wu, C.Y., Zhang, W., Shen, H., Xu, J.D., et al. (2021). Protective effects of Poria cocos and its components against cisplatin-induced intestinal injury. *J Ethnopharmacol* 269, 113722. doi: 10.1016/j.jep.2020.113722.
