# Supplementary material for: Effect of Shenling Baizhu powder on immunity to diarrheal disease: A systematic review and meta-analysis
Source: Front Pharmacol. 2022 Sep 14;13:938932. doi: 10.3389/fphar.2022.938932 (PMC9516002; doi:10.3389/fphar.2022.938932)
Supplement: Supplementary file 4 [file DataSheet1.pdf]

Supplementary Material

The product information of Shenling Baizhu Power(SLBZS)

Regarding the composition of SLBZS and how these were reported in the original study, they were produced by different manufacturers or hospitals according to Chinese Pharmacopoeia specifications, therefore, we list one of them here.

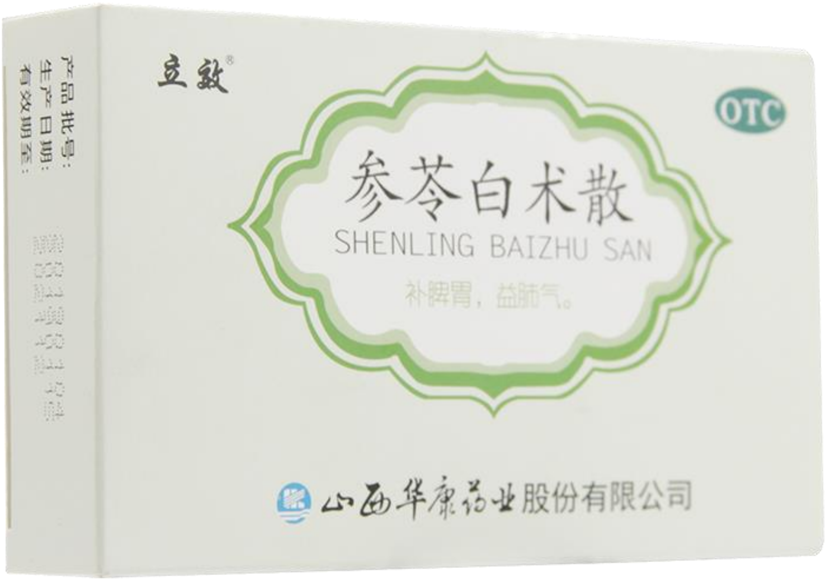

In the China Medical Information Platform (https://www.dayi.org.cn/), SLBZS is classified as a strengthening agent.

From the point of view of modern medicine, SLBZS is more likely to enhance the body's immunity and increase energy.

More details about the product information of SLBZS

Supplementary Table: Shenling Baizhu Powder Information Summary Table					
**Study**	**Formulation**	**Source**	**Species, Proportion**	**Quality control reported? (Y/N)**	**Chemical analysis reported? (Y/N)**
**BDY 2017**	SLBZS	The Affiliated Hospital of Gansu University of Chinese Medicine Chinese Medicine Department	Panax ginseng C.A.Mey [Araliaceae; ginseng radix] 10g: Atractylodes macrocephala Koidz [Asteraceae; atractylodis macrocephalae rhizoma]10g: Poria cocos [Polyporaceae;. Poria] 10g:Glycyrrhiza glabra L [Fabaceae; radix et rhizoma glycyrrhizae] 6g: Coicis Semen6g: Nelumbo nucifera Gaertn [Nelumbonaceae; lotus seed]6g: Wurfbainia villosa (Lour.) Skornick. & A.D.Poulsen [Zingiberaceae; amomi fructus] 4g: Lablab purpureus subsp. purpureus [Fabaceae; semen lablab album] 8g: Platycodon grandiflorus (Jacq.) A.DC [Campanulaceae; platycodonis radix] 4g: Dioscorea polystachya Turcz [Dioscoreaceae; dioscoreae rhizoma] 10g: Citrus ×aurantium L [Rutaceae; citri	NR	NR

			reticulatae pericarpium] 4g, (5:5:5:3:3:3:2:4:2:5:2)		
**CH 2007**	SLBZS	Shaanxi Lijun Hengxin Tang Pharmaceutical Co.	Panax ginseng C.A.Mey [Araliaceae; ginseng radix] 8g: Atractylodes macrocephala Koidz [Asteraceae; atractylodis macrocephalae rhizoma]8g: Poria cocos (Polyporaceae;. Poria) 8g:Glycyrrhiza glabra L [Fabaceae; radix et rhizoma glycyrrhizae]8g : Coix lacryma-jobi var. ma-yuen (Rom.Caill.) Stapf [Poaceae; coicis semen]4g: Nelumbo nucifera Gaertn [Nelumbonaceae; lotus seed]4g: Wurfbainia villosa (Lour.) Skornick. & A.D.Poulsen [Zingiberaceae; amomi fructus] 4g: Lablab purpureus subsp. purpureus [Fabaceae; semen lablab album] 6g: Platycodon grandiflorus (Jacq.) A.DC [Campanulaceae; platycodonis radix] 4g: Dioscorea polystachya Turcz [Dioscoreaceae; dioscoreae rhizoma] 8g, (4:4:4:4:2:2:2:3:2:4)	Y- Prepared according to Pharmacopoeia of the People's Republic of China	NR
**CCH 2004**	SLBZS	Gansu Pharmaceutical Group Gannan Pharmaceutical Factory	Panax ginseng C.A.Mey [Araliaceae; ginseng radix] 8g: Atractylodes macrocephala Koidz [Asteraceae; atractylodis macrocephalae rhizoma]8g: Poria cocos (Polyporaceae;. Poria) 8g:Glycyrrhiza glabra L [Fabaceae; radix et rhizoma glycyrrhizae]8g : Coix lacryma-jobi var. ma-yuen (Rom.Caill.) Stapf	Y- Prepared according to Pharmacopoeia of the People's Republic of China	NR

			[Poaceae; coicis semen]4g: Nelumbo nucifera Gaertn [Nelumbonaceae; lotus seed]4g: Wurfbainia villosa (Lour.) Skornick. & A.D.Poulsen [Zingiberaceae; amomi fructus] 4g: Lablab purpureus subsp. purpureus [Fabaceae; semen lablab album] 6g: Platycodon grandiflorus (Jacq.) A.DC [Campanulaceae; platycodonis radix] 4g: Dioscorea polystachya Turcz [Dioscoreaceae; dioscoreae rhizoma] 8g, (4:4:4:4:2:2:2:3:2:4)		
**DJL 2011**	SLBZS	Wuxi Hospital of Traditional Chinese Medicine	Panax ginseng C.A.Mey [Araliaceae; ginseng radix] 15g: Atractylodes macrocephala Koidz [Asteraceae; atractylodis macrocephalae rhizoma]30g: Poria cocos (Polyporaceae;. Poria) 15g: Coix lacryma-jobi var. ma-yuen (Rom.Caill.) Stapf [Poaceae; coicis semen]30g: Wurfbainia villosa (Lour.) Skornick. & A.D.Poulsen [Zingiberaceae; amomi fructus] 10g: Lablab purpureus subsp. purpureus [Fabaceae; semen lablab album] 10g: Dioscorea polystachya Turcz [Dioscoreaceae; dioscoreae rhizoma] 30g : Citrus × aurantium L [Rutaceae; citri reticulatae pericarpium] 10g (3:6:3:6:2:2:6:2)	NR	NR

**DYW 2007**	SLBZS	Shanxi Huakang Pharmaceutical Co.	Panax ginseng C.A.Mey [Araliaceae; ginseng radix] 8g: Atractylodes macrocephala Koidz [Asteraceae; atractylodis macrocephalae rhizoma]8g: Poria cocos (Polyporaceae;. Poria) 8g:Glycyrrhiza glabra L [Fabaceae; radix et rhizoma glycyrrhizae]8g : Coix lacryma-jobi var. ma-yuen (Rom.Caill.) Stapf [Poaceae; coicis semen]4g: Nelumbo nucifera Gaertn [Nelumbonaceae; lotus seed]4g: Wurfbainia villosa (Lour.) Skornick. & A.D.Poulsen [Zingiberaceae; amomi fructus] 4g: Lablab purpureus subsp. purpureus [Fabaceae; semen lablab album] 6g: Platycodon grandiflorus (Jacq.) A.DC [Campanulaceae; platycodonis radix] 4g: Dioscorea polystachya Turcz [Dioscoreaceae; dioscoreae rhizoma] 8g , (4:4:4:4:2:2:2:3:2:4)	Y- Prepared according to Pharmacopoeia of the People's Republic of China	NR
**FH 2004**	SLBZS	NR	Panax ginseng C.A.Mey [Araliaceae; ginseng radix] 20g: Atractylodes macrocephala Koidz [Asteraceae; atractylodis macrocephalae rhizoma]20g: Poria cocos [Polyporaceae;. Poria] 20g:Glycyrrhiza glabra L [Fabaceae; radix et rhizoma glycyrrhizae] 20g : Coix lacryma-jobi var. ma-yuen (Rom.Caill.) Stapf [Poaceae; coicis semen]10g: Nelumbo nucifera Gaertn [Nelumbonaceae; lotus seed]10g: Wurfbainia	NR	NR

			villosa (Lour.) Skornick. & A.D.Poulsen [Zingiberaceae; amomi fructus] 10g: Lablab purpureus subsp. purpureus [Fabaceae; semen lablab album] 15g: Platycodon grandiflorus (Jacq.) A.DC [Campanulaceae; platycodonis radix] 10g: Dioscorea polystachya Turcz [Dioscoreaceae; dioscoreae rhizoma] 40g, (4:4:4:4:2:2:2:3:2:4)		
**HHR 2008**	SLBZS	NR	Panax ginseng C.A.Mey [Araliaceae; ginseng radix] 8g: Atractylodes macrocephala Koidz [Asteraceae; atractylodis macrocephalae rhizoma]8g: Poria cocos [Polyporaceae;. Poria] 8g:Glycyrrhiza glabra L [Fabaceae; radix et rhizoma glycyrrhizae]8g : Coix lacryma-jobi var. ma-yuen (Rom.Caill.) Stapf [Poaceae; coicis semen]4g: Nelumbo nucifera Gaertn [Nelumbonaceae; lotus seed]4g: Wurfbainia villosa (Lour.) Skornick. & A.D.Poulsen [Zingiberaceae; amomi fructus] 4g: Lablab purpureus subsp. purpureus [Fabaceae; semen lablab album] 6g: Platycodon grandiflorus (Jacq.) A.DC [Campanulaceae; platycodonis radix] 4g: Dioscorea polystachya Turcz [Dioscoreaceae; dioscoreae rhizoma] 8g, (4:4:4:4:2:2:2:3:2:4)	NR	NR

**JYX 2016**	SLBZS	The Affiliated Hospital of Gansu University of Chinese Medicine Chinese Medicine Department	Panax ginseng C.A.Mey [Araliaceae; ginseng radix] 10g: Atractylodes macrocephala Koidz [Asteraceae; atractylodis macrocephalae rhizoma]10g: Poria cocos [Polyporaceae;. Poria] 10g:Glycyrrhiza glabra L [Fabaceae; radix et rhizoma glycyrrhizae] 6g: Coix lacryma-jobi var. ma-yuen (Rom.Caill.) Stapf [Poaceae; coicis semen]6g: Nelumbo nucifera Gaertn [Nelumbonaceae; lotus seed]6g: Wurfbainia villosa (Lour.) Skornick. & A.D.Poulsen [Zingiberaceae; amomi fructus] 4g: Lablab purpureus subsp. purpureus [Fabaceae; semen lablab album] 8g: Platycodon grandiflorus (Jacq.) A.DC [Campanulaceae; platycodonis radix] 4g: Dioscorea polystachya Turcz [Dioscoreaceae; dioscoreae rhizoma] 10g: Citrus ×aurantium L [Rutaceae; citri reticulatae pericarpium] 4g,(5:5:5:3:3:3:2:4:2:5:2)	NR	NR
**JMR 2010**	SLBZS	Shaanxi College of Traditional Chinese Medicine - Golden Autumn Outpatient Chinese Medicine Clinic	Panax ginseng C.A.Mey [Araliaceae; ginseng radix] 8g: Atractylodes macrocephala Koidz [Asteraceae; atractylodis macrocephalae rhizoma]8g: Poria cocos [Polyporaceae;. Poria] 8g:Glycyrrhiza glabra L [Fabaceae; radix et rhizoma glycyrrhizae]8g : Coix lacryma-jobi var. ma-yuen (Rom.Caill.) Stapf [Poaceae; coicis semen]4g: Nelumbo nucifera Gaertn	Y- Prepared according to Pharmacopoeia of the People's Republic of China	NR

			[Nelumbonaceae; lotus seed]4g: Wurfbainia villosa (Lour.) Skornick. & A.D.Poulsen [Zingiberaceae; amomi fructus] 4g: Lablab purpureus subsp. purpureus [Fabaceae; semen lablab album] 6g: Platycodon grandiflorus (Jacq.) A.DC [Campanulaceae; platycodonis radix] 4g: Dioscorea polystachya Turcz [Dioscoreaceae; dioscoreae rhizoma] 8g (4:4:4:4:2:2:2:3:2:4)		
**LJ 2016**	SLBZS	Kangmei Pharmaceutical Co.	Panax ginseng C.A.Mey [Araliaceae; ginseng radix] 15g: Atractylodes macrocephala Koidz [Asteraceae; atractylodis macrocephalae rhizoma]15g: Poria cocos [Polyporaceae;. Poria] 15g:Glycyrrhiza glabra L [Fabaceae; radix et rhizoma glycyrrhizae] 10g: Coix lacryma-jobi var. ma-yuen (Rom.Caill.) Stapf [Poaceae; coicis semen]10g: Nelumbo nucifera Gaertn [Nelumbonaceae; lotus seed]10g: Wurfbainia villosa (Lour.) Skornick. & A.D.Poulsen [Zingiberaceae; amomi fructus] 6g: Lablab purpureus subsp. purpureus [Fabaceae; semen lablab album] 12g: Platycodon grandiflorus (Jacq.) A.DC [Campanulaceae; platycodonis radix] 6g: Dioscorea polystachya Turcz [Dioscoreaceae; dioscoreae	NR	NR

			rhizoma] 15g , (15:15:15:10:10:10:6:12:6:15)		
**LWX 2015**	SLBZS	The First affiliated Hospital of Hunan Chinese Medicine College	Panax ginseng C.A.Mey [Araliaceae; ginseng radix] 10g: Atractylodes macrocephala Koidz [Asteraceae; atractylodis macrocephalae rhizoma]10g: Poria cocos [Polyporaceae;. Poria] 10g:Glycyrrhiza glabra L [Fabaceae; radix et rhizoma glycyrrhizae] 6g: Coix lacryma-jobi var. ma-yuen (Rom.Caill.) Stapf [Poaceae; coicis semen]6g: Nelumbo nucifera Gaertn [Nelumbonaceae; lotus seed]6g: Wurfbainia villosa (Lour.) Skornick. & A.D.Poulsen [Zingiberaceae; amomi fructus] 4g: Lablab purpureus subsp. purpureus [Fabaceae; semen lablab album] 8g: Platycodon grandiflorus (Jacq.) A.DC [Campanulaceae; platycodonis radix] 4g: Dioscorea polystachya Turcz [Dioscoreaceae; dioscoreae rhizoma] 10g, (5:5:5:3:3:3:2:4:2:5)	Y- Prepared according to Pharmacopoeia of the People's Republic of China	NR
**LXB 2014**	SLBZS	Shanxi Huakang Pharmaceutical Co.	Panax ginseng C.A.Mey [Araliaceae; ginseng radix] 8g: Atractylodes macrocephala Koidz [Asteraceae; atractylodis macrocephalae rhizoma]8g: Poria cocos [Polyporaceae;. Poria] 8g:Glycyrrhiza glabra L [Fabaceae; radix et rhizoma glycyrrhizae]8g : Coix lacryma-jobi var. ma-yuen (Rom.Caill.) Stapf	Y- Prepared according to Pharmacopoeia of the People's Republic of China	NR

			[Poaceae; coicis semen]4g: Nelumbo nucifera Gaertn [Nelumbonaceae; lotus seed]4g: Wurfbainia villosa (Lour.) Skornick. & A.D.Poulsen [Zingiberaceae; amomi fructus] 4g: Lablab purpureus subsp. purpureus [Fabaceae; semen lablab album] 6g: Platycodon grandiflorus (Jacq.) A.DC [Campanulaceae; platycodonis radix] 4g: Dioscorea polystachya Turcz [Dioscoreaceae; dioscoreae rhizoma] 8g, (4:4:4:4:2:2:2:3:2:4)		
**LY 2017**	SLBZS	Shaanxi University of Traditional Chinese Medicine Hospital Chinese Medicine Department	Panax ginseng C.A.Mey [Araliaceae; ginseng radix] 8g: Atractylodes macrocephala Koidz [Asteraceae; atractylodis macrocephalae rhizoma]8g: Poria cocos [Polyporaceae;. Poria] 8g:Glycyrrhiza glabra L [Fabaceae; radix et rhizoma glycyrrhizae]8g : Coix lacryma-jobi var. ma-yuen (Rom.Caill.) Stapf [Poaceae; coicis semen]4g: Nelumbo nucifera Gaertn [Nelumbonaceae; lotus seed]4g: Wurfbainia villosa (Lour.) Skornick. & A.D.Poulsen [Zingiberaceae; amomi fructus] 4g: Lablab purpureus subsp. purpureus [Fabaceae; semen lablab album] 6g: Platycodon grandiflorus (Jacq.) A.DC [Campanulaceae; platycodonis radix] 4g: Dioscorea polystachya Turcz [Dioscoreaceae; dioscoreae	Y- Prepared according to Pharmacopoeia of the People's Republic of China	NR

			rhizoma] 8g, (4:4:4:4:2:2:2:3:2:4)		
**LZH 2015**	SLBZS	Anhui University of Chinese Medicine Chinese Medicine Preparation Room	Panax ginseng C.A.Mey [Araliaceae; ginseng radix] 10g: Atractylodes macrocephala Koidz [Asteraceae; atractylodis macrocephalae rhizoma]10g: Poria cocos [Polyporaceae;. Poria] 10g:Glycyrrhiza glabra L [Fabaceae; radix et rhizoma glycyrrhizae] 6g: Coix lacryma-jobi var. ma-yuen (Rom.Caill.) Stapf [Poaceae; coicis semen]6g: Nelumbo nucifera Gaertn [Nelumbonaceae; lotus seed]6g: Wurfbainia villosa (Lour.) Skornick. & A.D.Poulsen [Zingiberaceae; amomi fructus] 4g: Lablab purpureus subsp. purpureus [Fabaceae; semen lablab album] 8g: Platycodon grandiflorus (Jacq.) A.DC [Campanulaceae; platycodonis radix] 4g: Dioscorea polystachya Turcz [Dioscoreaceae; dioscoreae rhizoma] 10g, (5:5:5:3:3:3:2:4:2:5)	NR	NR
**LZH 2020**	SLBZS	Anhui University of Chinese Medicine Chinese Medicine Preparation Room	Panax ginseng C.A.Mey [Araliaceae; ginseng radix] 10g: Atractylodes macrocephala Koidz [Asteraceae; atractylodis macrocephalae rhizoma]10g: Poria cocos [Polyporaceae;. Poria] 10g:Glycyrrhiza glabra L [Fabaceae; radix et rhizoma glycyrrhizae] 6g: Coix lacryma-jobi var. ma-yuen (Rom.Caill.) Stapf	NR	NR

			[Poaceae; coicis semen]6g: Nelumbo nucifera Gaertn [Nelumbonaceae; lotus seed]6g: Wurfbainia villosa (Lour.) Skornick. & A.D.Poulsen [Zingiberaceae; amomi fructus] 4g: Lablab purpureus subsp. purpureus [Fabaceae; semen lablab album] 8g: Platycodon grandiflorus (Jacq.) A.DC [Campanulaceae; platycodonis radix] 4g: Dioscorea polystachya Turcz [Dioscoreaceae; dioscoreae rhizoma] 10g, (5:5:5:3:3:3:2:4:2:5)		
**LHW 2010**	SLBZS	Shaanxi College of Traditional Chinese Medicine - Golden Autumn Outpatient Chinese Medicine Clinic	Panax ginseng C.A.Mey [Araliaceae; ginseng radix] 8g: Atractylodes macrocephala Koidz [Asteraceae; atractylodis macrocephalae rhizoma]8g: Poria cocos [Polyporaceae;. Poria] 8g:Glycyrrhiza glabra L [Fabaceae; radix et rhizoma glycyrrhizae]8g : Coix lacryma-jobi var. ma-yuen (Rom.Caill.) Stapf [Poaceae; coicis semen]4g: Nelumbo nucifera Gaertn [Nelumbonaceae; lotus seed]4g: Wurfbainia villosa (Lour.) Skornick. & A.D.Poulsen [Zingiberaceae; amomi fructus] 4g: Lablab purpureus subsp. purpureus [Fabaceae; semen lablab album] 6g: Platycodon grandiflorus (Jacq.) A.DC [Campanulaceae; platycodonis radix] 4g: Dioscorea polystachya Turcz [Dioscoreaceae; dioscoreae	Y- Prepared according to Pharmacopoeia of the People's Republic of China	NR

			rhizoma] 8g, (4:4:4:4:2:2:2:3:2:4)		
**SY 2018**	SLBZS	Yingpan Pharmacy	Panax ginseng C.A.Mey [Araliaceae; ginseng radix] 8g: Atractylodes macrocephala Koidz [Asteraceae; atractylodis macrocephalae rhizoma]8g: Poria cocos [Polyporaceae;. Poria] 8g:Glycyrrhiza glabra L [Fabaceae; radix et rhizoma glycyrrhizae]8g : Coix lacryma-jobi var. ma-yuen (Rom.Caill.) Stapf [Poaceae; coicis semen]4g: Nelumbo nucifera Gaertn [Nelumbonaceae; lotus seed]4g: Wurfbainia villosa (Lour.) Skornick. & A.D.Poulsen [Zingiberaceae; amomi fructus] 4g: Lablab purpureus subsp. purpureus [Fabaceae; semen lablab album] 6g: Platycodon grandiflorus (Jacq.) A.DC [Campanulaceae; platycodonis radix] 4g: Dioscorea polystachya Turcz [Dioscoreaceae; dioscoreae rhizoma] 8g, (4:4:4:4:2:2:2:3:2:4)	NR	NR
**SSZ 2020**	SLBZS	Beijing Tongrentang Company Limited	Panax ginseng C.A.Mey [Araliaceae; ginseng radix] 8g: Atractylodes macrocephala Koidz [Asteraceae; atractylodis macrocephalae rhizoma]8g: Poria cocos [Polyporaceae;. Poria] 8g:Glycyrrhiza glabra L [Fabaceae; radix et rhizoma glycyrrhizae]8g : Coix lacryma-jobi var. ma-yuen (Rom.Caill.) Stapf	Y- Prepared according to Pharmacopoeia of the People's Republic of China	NR

			[Poaceae; coicis semen]4g: Nelumbo nucifera Gaertn [Nelumbonaceae; lotus seed]4g: Wurfbainia villosa (Lour.) Skornick. & A.D.Poulsen [Zingiberaceae; amomi fructus] 4g: Lablab purpureus subsp. purpureus [Fabaceae; semen lablab album] 6g: Platycodon grandiflorus (Jacq.) A.DC [Campanulaceae; platycodonis radix] 4g: Dioscorea polystachya Turcz [Dioscoreaceae; dioscoreae rhizoma] 8g, (4:4:4:4:2:2:2:3:2:4)		
**TJS 2021**	SLBZS	Pharmacy of the Affiliated Hospital of Heilongjiang University of Chinese Medicine	Panax ginseng C.A.Mey [Araliaceae; ginseng radix] 15g: Atractylodes macrocephala Koidz [Asteraceae; atractylodis macrocephalae rhizoma]15g: Poria cocos [Polyporaceae;. Poria] 15g:Glycyrrhiza glabra L [Fabaceae; radix et rhizoma glycyrrhizae] 9g: Coix lacryma-jobi var. ma-yuen (Rom.Caill.) Stapf [Poaceae; coicis semen]9g: Nelumbo nucifera Gaertn [Nelumbonaceae; lotus seed]9g: Wurfbainia villosa (Lour.) Skornick. & A.D.Poulsen [Zingiberaceae; amomi fructus] 6g: Lablab purpureus subsp. purpureus [Fabaceae; semen lablab album] 12g: Platycodon grandiflorus (Jacq.) A.DC [Campanulaceae; platycodonis radix] 6g: Dioscorea polystachya Turcz [Dioscoreaceae; dioscoreae	NR	NR

			rhizoma] 15g: Citrus ×aurantium L [Rutaceae; citri reticulatae pericarpium] 6g, (5:5:5:3:3:3:2:4:2:5:2)		
**YY 2019**	SLBZS	Yunnan Baiyao Group Co.	Panax ginseng C.A.Mey [Araliaceae; ginseng radix] 8g: Atractylodes macrocephala Koidz [Asteraceae; atractylodis macrocephalae rhizoma]8g: Poria cocos [Polyporaceae;. Poria] 8g:Glycyrrhiza glabra L [Fabaceae; radix et rhizoma glycyrrhizae]8g : Coix lacryma-jobi var. ma-yuen (Rom.Caill.) Stapf [Poaceae; coicis semen]4g: Nelumbo nucifera Gaertn [Nelumbonaceae; lotus seed]4g: Wurfbainia villosa (Lour.) Skornick. & A.D.Poulsen [Zingiberaceae; amomi fructus] 4g: Lablab purpureus subsp. purpureus [Fabaceae; semen lablab album] 6g: Platycodon grandiflorus (Jacq.) A.DC [Campanulaceae; platycodonis radix] 4g: Dioscorea polystachya Turcz [Dioscoreaceae; dioscoreae rhizoma] 8g, (4:4:4:4:2:2:2:3:2:4)	Y- Prepared according to Pharmacopoeia of the People's Republic of China	NR
**YHQ 2017**	SLBZS	Affiliated Hospital of Gansu College of Traditional Chinese Medicine	Panax ginseng C.A.Mey [Araliaceae; ginseng radix] 10g: Atractylodes macrocephala Koidz [Asteraceae; atractylodis macrocephalae rhizoma]10g: Poria cocos [Polyporaceae;. Poria] 10g:Glycyrrhiza glabra L [Fabaceae; radix et rhizoma glycyrrhizae] 6g: Coix	NR	NR

			lacryma-jobi var. ma-yuen (Rom.Caill.) Stapf [Poaceae; coicis semen]6g: Nelumbo nucifera Gaertn [Nelumbonaceae; lotus seed]6g: Wurfbainia villosa (Lour.) Skornick. & A.D.Poulsen [Zingiberaceae; amomi fructus] 4g: Lablab purpureus subsp. purpureus [Fabaceae; semen lablab album] 8g: Platycodon grandiflorus (Jacq.) A.DC [Campanulaceae; platycodonis radix] 4g: Dioscorea polystachya Turcz [Dioscoreaceae; dioscoreae rhizoma] 10g: Citrus ×aurantium L [Rutaceae; citri reticulatae pericarpium] 4g, (5:5:5:3:3:3:2:4:2:5:2)		
**YHQ 2018**	SLBZS	Affiliated Hospital of Gansu College of Traditional Chinese Medicine	Panax ginseng C.A.Mey [Araliaceae; ginseng radix] 10g: Atractylodes macrocephala Koidz [Asteraceae; atractylodis macrocephalae rhizoma]10g: Poria cocos [Polyporaceae;. Poria] 10g:Glycyrrhiza glabra L [Fabaceae; radix et rhizoma glycyrrhizae] 6g: Coix lacryma-jobi var. ma-yuen (Rom.Caill.) Stapf [Poaceae; coicis semen]6g: Nelumbo nucifera Gaertn [Nelumbonaceae; lotus seed]6g: Wurfbainia villosa (Lour.) Skornick. & A.D.Poulsen [Zingiberaceae; amomi fructus] 4g: Lablab purpureus subsp. purpureus [Fabaceae; semen lablab album] 8g: Platycodon grandiflorus (Jacq.) A.DC	NR	NR

			[Campanulaceae; platycodonis radix] 4g: Dioscorea polystachya Turcz [Dioscoreaceae; dioscoreae rhizoma] 10g: Citrus ×aurantium L [Rutaceae; citri reticulatae pericarpium] 4g, (5:5:5:3:3:3:2:4:2:5:2)		
**ZJJ 2019**	SLBZS	The First Affiliated Hospital of Guangzhou University of Chinese Medicine	Panax ginseng C.A.Mey [Araliaceae; ginseng radix] 15g: Atractylodes macrocephala Koidz [Asteraceae; atractylodis macrocephalae rhizoma]15g: Poria cocos [Polyporaceae;. Poria] 15g:Glycyrrhiza glabra L [Fabaceae; radix et rhizoma glycyrrhizae] 9g: Nelumbo nucifera Gaertn [Nelumbonaceae; lotus seed]9g: Dioscorea polystachya Turcz [Dioscoreaceae; dioscoreae rhizoma] 9g: Platycodon grandiflorus (Jacq.) A.DC [Campanulaceae; platycodonis radix] 15g: Wurfbainia villosa (Lour.) Skornick. & A.D.Poulsen [Zingiberaceae; amomi fructus] 9g, (5:5:5:3:3:5:3:2)	Y- Prepared according to Pharmacopoeia of the People's Republic of China	NR
**ZJL 2010**	SLBZS	Shaanxi College of Traditional Chinese Medicine - Golden Autumn Outpatient Chinese Medicine	Panax ginseng C.A.Mey [Araliaceae; ginseng radix] 8g: Atractylodes macrocephala Koidz [Asteraceae; atractylodis macrocephalae rhizoma]8g: Poria cocos [Polyporaceae;. Poria] 8g:Glycyrrhiza glabra L [Fabaceae; radix et rhizoma glycyrrhizae]8g : Coix lacryma-jobi var. ma-yuen (Rom.Caill.) Stapf	Y- Prepared according to Pharmacopoeia of the People's Republic of China	NR

		Clinic	[Poaceae; coicis semen]4g: Nelumbo nucifera Gaertn [Nelumbonaceae; lotus seed]4g: Wurfbainia villosa (Lour.) Skornick. & A.D.Poulsen [Zingiberaceae; amomi fructus] 4g: Lablab purpureus subsp. purpureus [Fabaceae; semen lablab album] 6g: Platycodon grandiflorus (Jacq.) A.DC [Campanulaceae; platycodonis radix] 4g: Dioscorea polystachya Turcz [Dioscoreaceae; dioscoreae rhizoma] 8g, (4:4:4:4:2:2:2:3:2:4)		
**ZYF 2006**	SLBZS	NR	Panax ginseng C.A.Mey [Araliaceae; ginseng radix] 20g: Atractylodes macrocephala Koidz [Asteraceae; atractylodis macrocephalae rhizoma]20g: Poria cocos [Polyporaceae;. Poria] 20g:Glycyrrhiza glabra L [Fabaceae; radix et rhizoma glycyrrhizae] 20g : Coix lacryma-jobi var. ma-yuen (Rom.Caill.) Stapf [Poaceae; coicis semen]10g: Nelumbo nucifera Gaertn [Nelumbonaceae; lotus seed]10g: Wurfbainia villosa (Lour.) Skornick. & A.D.Poulsen [Zingiberaceae; amomi fructus] 10g: Lablab purpureus subsp. purpureus [Fabaceae; semen lablab album] 15g: Platycodon grandiflorus (Jacq.) A.DC [Campanulaceae; platycodonis radix] 10g: Dioscorea polystachya Turcz [Dioscoreaceae;	Y- Y- Prepared according to formulary of peaceful benevolent dispensary	NR

			dioscoreae rhizoma] 20g, (4:4:4:4:2:2:2:3:2:4)		
**ZH 2019**	SLBZS	The First Affiliated Hospital of Anhui University of Chinese Medicine, Chinese Medicine Preparation Room	Panax ginseng C.A.Mey [Araliaceae; ginseng radix] 8g: Atractylodes macrocephala Koidz [Asteraceae; atractylodis macrocephalae rhizoma]8g: Poria cocos [Polyporaceae;. Poria] 8g:Glycyrrhiza glabra L [Fabaceae; radix et rhizoma glycyrrhizae]8g : Coix lacryma-jobi var. ma-yuen (Rom.Caill.) Stapf [Poaceae; coicis semen]4g: Nelumbo nucifera Gaertn [Nelumbonaceae; lotus seed]4g: Wurfbainia villosa (Lour.) Skornick. & A.D.Poulsen [Zingiberaceae; amomi fructus] 4g: Lablab purpureus subsp. purpureus [Fabaceae; semen lablab album] 6g: Platycodon grandiflorus (Jacq.) A.DC [Campanulaceae; platycodonis radix] 4g: Dioscorea polystachya Turcz [Dioscoreaceae; dioscoreae rhizoma] 8g, (4:4:4:4:2:2:2:3:2:4)	NR	NR

Note: Shenling Baizhu Powder, SLBZS; NR, No report

The chemical characterisation of Shenling Baizhu power

The information comes from the Pharmacopoeia of the People's Republic of China (available at: https://db.ouryao.com/yd2020/) and Drug Standards Database (available at: https://www.drugfuture.com/standard/).

Chinese phonetic alphabet: Shenling Baizhu San

Drug Composition: Panax ginseng C.A.Mey [Araliaceae; ginseng radix]100g, Poria cocos (Polyporaceae;. Poria)100g, Atractylodes macrocephala Koidz [Asteraceae; atractylodis macrocephalae rhizoma]100g, Dioscorea polystachya Turcz [Dioscoreaceae; dioscoreae rhizoma]100g, Lablab purpureus subsp. purpureus [Fabaceae; semen lablab album]75g, Nelumbo nucifera Gaertn [Nelumbonaceae; lotus seed] 50g, Coix lacryma-jobi var. ma-yuen (Rom.Caill.) Stapf [Poaceae; coicis semen]50g, Wurfbainia villosa (Lour.) Skornick. & A.D.Poulsen [Zingiberaceae; amomi fructus]50g, Platycodon grandiflorus (Jacq.) A.DC [Campanulaceae; platycodonis radix]50g, Glycyrrhiza glabra L [Fabaceae; radix et rhizoma glycyrrhizae]100g

Drug making method: The above ten drugs, crushed into fine powder, sieve, mix.

Properties: Yellow to greyish-yellow powder; The air is sweet and the taste is sweet.

Identification:

(1) Take this product and observe it under a microscope: the irregular branched clumps are colorless and dissolve in the chloral hydrate test solution; the hyphae are colorless or light brown, with a diameter of 4-6 μm (Poria).Calcium oxalate cluster crystals are 20-68 μm in diameter, with sharp edges and corners (ginseng). Calcium oxalate needle crystals are small, 10-32 μm long, irregularly packed in parenchyma cells (Atractylodes). Calcium oxalate needle crystal bundles exist in mucous cells, with a length of 80-240 μm and a needle crystal diameter of 2-8 μm (yam). The parenchyma cells around the fiber bundles contain cubes of calcium oxalate, forming crystal fibers (licorice). Pigment cells are yellow-brown or reddish-brown, and the surface view is rectangular, polygonal or circular (lotus seed). Seed coat palisade cells are 80-150 μm long (white lentil). Endothelial thick-walled cells are yellow-brown or

brown-red, polygonal in surface view, with thick walls, and the cell cavity contains siliceous blocks (Amomum). The connecting milk ducts are 14-25 μm in diameter, and contain pale yellow particles (platycodon).

(2) Take 4.5g of this product, add 40ml of chloroform, heat under reflux for 1 hour, filter, evaporate the chloroform from the residue, add 50ml of methanol, heat under reflux for 1 hour, filter, evaporate the filtrate to dryness, and use methanol for the residue Dissolve in 5ml, add it to a neutral alumina column (100-120 mesh, 15g, inner diameter 1-1.5cm), elute with 150ml of 40% methanol, collect the eluate, evaporate to dryness, dissolve the residue in 30ml of water, and dissolve it with water. Saturated n-butanol was shaken and extracted twice, each 25ml, the extracts were combined, washed three times with water, each 20ml, the n-butanol was evaporated to dryness, the residue was dissolved in 0.5ml methanol, and used as the test solution. In addition, 1 g of ginseng reference medicinal material and licorice reference medicinal material were taken, respectively, and the reference medicinal material solution was prepared in the same way. According to the thin-layer chromatography (general rule 0502) test, draw 1 μl of each of the above three solutions, and spot them on the same silica gel G thin-layer plate respectively.) The lower layer solution placed below 10 ℃ is the developing agent, unfold, take out, air dry, spray with sulfuric acid ethanol solution (1→10), heat at 105°C for 5-10 minutes, and inspect under ultraviolet light (365nm). In the chromatogram of the test substance, fluorescent spots of the same color were displayed on the corresponding positions of the chromatograms of the two reference medicinal materials.

Check: It should comply with the relevant regulations under the powder (Pharmacopoeia of the People's Republic of China; General rules for dispersions 0115; available at: https://db.ouryao.com/yd2020/view.php?id=f0ed7309b9).

Powder refers to the raw drug or the dry powder preparation prepared by pulverizing and homogeneously mixing it with suitable excipients. Powder can be divided into oral powder and topical powder. Oral powders are generally dissolved or dispersed in water, diluent or other liquids to be taken, or can be taken directly with water. Topical powders can be applied to the skin, mouth, throat, cavities, etc. Powders designed for treatment, prevention and lubrication of the skin can also be

referred to as sprinkles or dusting powders. Powders should comply with the following relevant regulations during production and storage.

1. The raw materials for the preparation of powders should be crushed. Unless otherwise specified, powders for oral use are fine powders, and powders for pediatric and topical use should be the finest powders.

2. The powder may contain or not contain auxiliary materials. Oral powders can also be added with flavorings, fragrances, coloring agents, etc., if necessary.

3. In order to prevent gastric acid from destroying the active ingredients in the biological product powder, the powder diluent can be formulated with an ingredient that neutralizes gastric acid.

4.The powder should be dry, loose, evenly mixed, and consistent in color. When preparing powders containing toxic drugs, precious drugs or small doses of drugs, the compounding method should be used to mix and sieve.

5. Powder can be packed in single-dose packages (sub-packages), and multi-dose packages should be accompanied by sub-dose utensils. Oral powders containing toxic drugs should be packaged in single doses.

6. Unless otherwise specified, powders should be stored in airtight areas, and powders containing volatile APIs or easily moisture-absorbing APIs should be sealed and stored. Biological products should be packaged in moisture-proof materials.

7. If the powder is used for the treatment of burns, if it is a non-sterile preparation, it should be marked with "non-sterile preparation" on the label; "this product is a non-sterile preparation" should be indicated in the product manual, and it should be clearly stated under the indication. "Used for minor burns (I °or shallow II °)"; "should be used as directed by a doctor" under the precautions.

Unless otherwise specified, powders should be checked accordingly as follows.

[Particle size] Unless otherwise specified, the topical powder for chemical medicines, the topical powder for traditional Chinese medicine for burns or severe

trauma and the powder for pediatric use shall be inspected according to the following methods and shall comply with the regulations. Inspection method Unless otherwise specified, take 10g of the test sample, accurately weigh it, and measure it according to the particle size and particle size distribution determination method (general rule 0982 single sieving method). The weight of the chemical powder that passes through the No. 7 sieve (the traditional Chinese medicine passes through the No. 6 sieve) shall not be less than 95%.

[Appearance uniformity] Take an appropriate amount of the test sample, put it on smooth paper, lay it flat for about 5cm2, flatten its surface, and observe it in a bright place. The color should be uniform, and there should be no patterns and stains.

[Moisture] The traditional Chinese medicine powder shall be determined according to the moisture determination method (General Rule 0832), and shall not exceed 9.0% unless otherwise specified.

[Loss on drying] For chemical drugs and biological powders, unless otherwise specified, take the test sample, measure it according to the method of loss on drying (General Rule 0831), and dry it at 105 ℃ to constant weight, and the weight loss shall not exceed 2.0%.

[Differences in loading volume] Powders in single-dose packages shall be inspected according to the following methods and shall comply with the regulations.

Investigating method Unless otherwise specified, take 10 bags (bottles) of the test sample, accurately weigh the contents of each bag (bottle), and obtain the contents and average contents. Comparing the filling volume of each bag (bottle) with the average filling volume [for powders with a marked filling volume, the filling volume of each bag (bottle) should be compared with the marked filling volume], according to the provisions in the table, if the powder exceeds the limit of the filling volume difference Powders shall not be more than 2 bags (bottles), and one bag (bottle) shall not exceed 1 times the limit of the difference in filling amount.

Average load or	Loading difference limit	Filling Variation Limits

marked load	(Chinese medicine, chemical medicine)	(Biological Products)
0.1g and below	±15%	±15%
Above 0.1g to 0.5g	±10%	±10%
Above 0.5g to 1.5g	±8%	±7.5%
Above 1.5g to 6.0g	±7%	±5%
Above 6.0g	±5%	±3%

For chemical drugs and biological product powders that require inspection of content uniformity, the inspection of the difference in loading is generally no longer carried out.

[Loading capacity] Unless otherwise specified, powders in multi-dose packages shall be inspected according to the minimum filling capacity inspection method (General Rule 0942) and shall comply with the regulations.

[Sterile] Unless otherwise specified, for burns [except for minor burns (I °or superficial II °)], severe trauma or clinically necessary sterile topical powders, according to the sterility test method (General Principles) 1101) inspection shall comply with the regulations.

[Microbial limit] Unless otherwise specified, the inspection shall be conducted according to the microbial limit of non-sterile products: microbial count method (general rule 1105) and control bacteria inspection method (general rule 1106) and non-sterile drug microbial limit standard (general rule 1107) inspection, should be Compliance. For powders of biological products that are required to be inspected for miscellaneous bacteria, microbial limit inspection is not required.

Functions and Indications: Invigorating the spleen and stomach, benefiting lung qi. For weak spleen and stomach, decreased appetite, diarrhea, shortness of breath, cough, drowsiness and weakness of limbs.

Usage and dosage: Oral. 6-9g once, 2-3 times a day.

Adverse reactions: Not yet clear.

Contraindications: Not yet clear.

Precautions:

1. Avoid indigestible food.

2. Patients with cold and fever should not take it.

3. Those with severe chronic diseases such as high blood pressure, heart disease, liver disease, diabetes, and kidney disease should take it under the guidance of a physician.

4. Children, pregnant women and lactating women should take it under the guidance of a physician.

5. If the symptoms are not relieved after taking the medicine for 4 weeks, you should go to the hospital for treatment.

6. Those who are allergic to this product are forbidden to use it, and those with allergies should use it with caution.

7. It is forbidden to use when the properties of this product are changed.

8. Children must be used under adult supervision.

9. Please keep this product out of the reach of children.

10. If you are using other medicines, please consult your doctor or pharmacist before using this product

Storage: Sealed.
